# ﻿Annotated checklist of the operculated land snails from Thailand (Mollusca, Gastropoda, Caenogastropoda): the family Pupinidae, with descriptions of several new species and subspecies, and notes on classification of *Pupina* Vignard, 1829 and *Pupinella* Gray, 1850 from mainland Southeast Asia

**DOI:** 10.3897/zookeys.1119.85400

**Published:** 2022-08-25

**Authors:** Parin Jirapatrasilp, Chirasak Sutcharit, Somsak Panha

**Affiliations:** 1 Animal Systematics Research Unit, Department of Biology, Faculty of Science, Chulalongkorn University, Bangkok 10330, Thailand Chulalongkorn University Bangkok Thailand; 2 Academy of Science, The Royal Society of Thailand, Bangkok 10300, Thailand Academy of Science, The Royal Society of Thailand Bangkok Thailand

**Keywords:** Biodiversity, malacofauna, “prosobranch”, systematics, taxonomy

## Abstract

Thailand is located at the crossroads of several biogeographical regions, and boasts a high level of biodiversity, especially among the malacofauna. The most recent checklist of land snail species in Thailand was compiled more than twenty years ago, and so this checklist needs revision and the addition of newly discovered taxa. This study updates the taxonomy and species list of the operculated land snail family Pupinidae from Thailand. This snail family is diverse and abundant, and can be found in various natural habitats in Southeast Asia. Although the taxonomy of some Southeast Asian pupinid genera has been reviewed, studies of *Pupina* Vignard, 1829, which contains the highest number of species, and a lesser-known genus *Pupinella* Gray, 1850 are still lacking. Herein we present an annotated checklist with an up-to-date systematic framework of the Pupinidae in Thailand based on both field investigations and literature surveys, and include the taxonomic treatment of all *Pupina* and *Pupinella* species from mainland Southeast Asia.

This annotated checklist contains 30 nominal species and two subspecies from seven genera currently known to occur in Thailand. We describe two species of *Pseudopomatias* (*P.doiangkhangensis* Jirapatrasilp, **sp. nov.** and *P.pallgergelyi* Jirapatrasilp, **sp. nov.**), five species and one subspecies of *Pupina* (*P.bensoni* Jirapatrasilp, **sp. nov.**, *P.bilabiata* Jirapatrasilp, **sp. nov.**, *P.godwinausteni* Jirapatrasilp, **sp. nov.**, *P.latisulci* Jirapatrasilp, **sp. nov.**, *P.stoliczkai* Jirapatrasilp, **sp. nov.**, and *P.dorriisanensis* Jirapatrasilp, **ssp. nov.**) as new to science. New records of *Coptocheilussumatranus*, *Pupinellamansuyi*, and *Rhaphaulustonkinensis* are also reported from Thailand. The mainland Southeast Asian *Pupina* species are classified into three species groups (*Pupinaartata* group, *Pupinaarula* group, and *Pupinaaureola* group) based on the distinction of shell teeth and canals, and operculum. Three species formerly in *Pupina* from Vietnam are allocated to *Pupinella* (*P.illustris***comb. nov.**, *P.sonlaensis***comb. nov.**, and *P.thaitranbaii***comb. nov.**) due to the presence of a funnel-like anterior canal.

## ﻿Introduction

Thailand boasts a high diversity of both flora and fauna, as the country is located within the Indo-Burma biodiversity hotspot, which is deemed the “crossroads” of three biogeographical regions: southern China in the north, the Indian subcontinent and the Himalayas in the west, and Sundaland in the south (Ashton 1990; Myers et al. 2000; Tordoff et al. 2012). Thailand’s geography can be divided into (i) the hill ranges in the north, (ii) the central plain, (iii) the Khorat Plateau, and (iv) the coastal plains of southeastern Thailand, Kra Isthmus and the Malay Peninsula (Gupta 2005). Each distinct geographical area has unique climatic, geological, and vegetational conditions that provide highly diverse habitats, such as limestone karsts that house several endemic species (e.g., Latinne et al. 2013; Suwannapoom et al. 2018). However, various groups of terrestrial invertebrates have still received less attention compared to their vertebrate counterparts, which have been more frequently and comprehensively inventoried (e.g., amphibians: Chan-ard 2003; Chuaynkern and Chuaynkern 2012; Niyomwan et al. 2019).

Although the terrestrial malacofauna exhibits a particularly high diversity, studies on species diversity in Thailand have only been sporadically published in the past (Suvatti 1938, 1950; Solem 1966; Panha 1996; Hemmen and Hemmen 2001). In the mid-nineteenth century, the earliest study of Thai land snails was done by William A. Haines, who had retrieved specimens from Dr. Samuel R. House, an American missionary (Haines 1855). As Thailand (formerly known as Siam) was never colonised by any Western countries like its neighbours were, there were no prominent naturalists who extensively collected and studied land snails in the country, as Henri Mouhot and Auguste Pavie did in French Indochina (present-day Cambodia, Laos, and Vietnam; Inkhavilay et al. 2019), and Henry H. Godwin-Austen and several other British naturalists did in Myanmar and Malaysia (Godwin-Austen 1882–1920). However, since the expeditions led by H. Mouhot and A. Pavie surveyed parts of present-day Thailand (Inkhavilay et al. 2019), some Thai land snails were described from the Mouhot collections under Hugh Cuming’s legacy, primarily by Louis Pfeiffer (1856a, 1860), and from the Pavie collections by several French and Belgian malacologists (Fischer and Dautzenberg 1904). Later, L. Pfeiffer (1862) also described more new species from Siam. Another important study was done by Eduard von Martens (1867), who worked on the collections from the Prussian Expedition to East Asia during 1859–1862.

Thereafter, and until the twentieth century, studies on Thai land snails were fragmentary and occasionally done by western malacologists who obtained specimens from merchants, naturalists and missionaries visiting Thailand. For example, Otto F. von Möllendorff studied land snails and described new species based on Carl Roebelen’s collections from the Samui Islands and based on Hans Fruhstorfer’s collections from several localities (von Möllendorff 1894, 1902b). William T. Blanford studied and described two new species from specimens collected by William M. Daly in Lamphun and Phitsanulok (Blanford 1902, 1903). John R. le B. Tomlin studied and described new species from specimens collected by Dr. Arthur Kerr from various parts of Thailand (Tomlin 1929, 1931, 1932a, b), and later Albert E. Salisbury described one new species based on Tomlin’s collection (Salisbury 1949). Paul Bartsch described one new species from Kao Sabab, and Fredrik E. Loosjes described one new subspecies from Doi Ang Ka, based on specimens collected by Hugh M. Smith, the Fishery Advisor to the Government (Bartsch 1932; Loosjes 1950). Fritz Haas reported some land snail species collected during the Rush Watkins Zoological Expedition to Siam in 1949 (Haas 1952). Alan Solem studied and described new species and genera based on collections from several Danish expeditions in northern, eastern and western Thailand during 1958–1964 (Solem 1966).

More recently, land snail research in Thailand was boosted after SP began studying Thai land malacofauna in the 1990s (Panha 1996). A number of operculated land snails from the families Alycaeidae, Cyclophoridae and Diplommatinidae were described (Panha and Burch 1998, 2005; Panha and Patamakanthin 2001; Nantarat et al. 2014, 2019; Sutcharit et al. 2014; Jirapatrasilp et al 2021). However, most malacological studies focused on pulmonate land snails, e.g., the families Ariophantidae (Pholyotha et al. 2020; Sutcharit and Panha 2021), Camaenidae (Sutcharit and Panha 2006), Gastrocoptidae (Panha and Burch 2005), and Streptaxidae (Siriboon et al. 2014a, b). The 20-year work of SP and his colleagues has culminated in a recent inventory and book on Thai land snails (BEDO 2017; Sutcharit et al. 2018).

The family Pupinidae Pfeiffer, 1853 belongs to the group of operculated land snails in the superfamily Cyclophoroidea, subclass Caenogastropoda (Bouchet et al. 2017). Although Tielecke (1940) characterised this family by its pupoid shell shape and long bursa copulatrix, several pupinid groups have no pupoid shells, e.g., *Pseudopomatias* and its relatives (Páll-Gergely et al. 2015), and the entire subfamily Liareinae (Powell 1979; Marshall and Barker 2007). The shell shape alone is thus not diagnostic and anatomical information in several groups is still lacking. Approximately 30 extant and ten extinct genera are recognised within this family, the distribution of which ranges from South and East Asia to Southeast Asia, Melanesia, Micronesia and part of Australia (MolluscaBase 2022; see also literature cited in Kongim et al. 2013). Ten pupinid genera have been recorded from mainland Southeast Asia (Kobelt 1902; Páll-Gergely et al. 2015; Thach 2017), where they can be found in various natural habitats and are abundant in limestone areas.

Recently, the taxonomy of some genera has been reviewed; i.e., *Coptocheilus* Gould, 1862 (Páll-Gergely et al. 2019; Bui and Páll-Gergely 2020), *Pollicaria* Gould, 1856 (Kongim et al. 2013), *Rhaphaulus* Pfeiffer, 1856 and *Streptaulus* Benson, 1857 (Páll-Gergely et al. 2014, 2017), and *Pseudopomatias* Möllendorff, 1885 and *Vargapupa* Páll-Gergely, 2015 (Páll-Gergely et al. 2015; Páll-Gergely and Grego 2019). Another land snail genus, *Notharinia* Vermeulen, Phung & Truong, 2007 was originally classified in the Pupinidae based on a set of shell characters shared with *Pseudopomatias*. *Notharinia* also lacks a circular constriction inside the ultimate or penultimate whorl, the presence of which is typical in the Diplommatinidae (Vermeulen et al. 2007; Marzuki and Foon 2016). However, *Notharinia* was later transferred to the Diplommatinidae, due to a similar shell size and shape to *Arinia* H. Adams & A. Adams, 1856, a possession of a distinctly oblique apex which commonly occurs in diplommatinids, and the discovery of *Notharinia* species with a constriction in the spire (Marzuki and Foon 2016; Vermeulen et al. 2019). The studies on *Pupina* Vignard, 1829, which contains the highest number of species, have been restricted to particular geographical areas (Do 2017; Tripathy and Sajan 2019), whereas other, less speciose genera, including *Barnaia* Thach, 2017, *Pupinella* Grey, 1850, and *Tortulosa* Gray, 1847 still remain unexamined.

This study is the first comprehensive work to update the taxonomy and species list of operculated land snails in the family Pupinidae in Thailand, several species of which are recognised as new to science. We also revise the genera *Pupina* and *Pupinella* from mainland Southeast Asia. This paper provides a checklist of species compiled from the literature and based on specimens collected during field surveys throughout the country over the past 28 years (1995–2022). It includes taxonomic updates, illustrations of type specimens (when possible), and photos of newly collected specimens. We hope that this paper will contribute to a better understanding of the operculated land snail biodiversity in Thailand, the knowledge of which can be applied in ecological, agricultural, and pharmaceutical research, and hope to inspire future generations to learn and conserve the country’s land snail heritages.

## ﻿Materials and methods

### ﻿Sources

**Figure 1.**
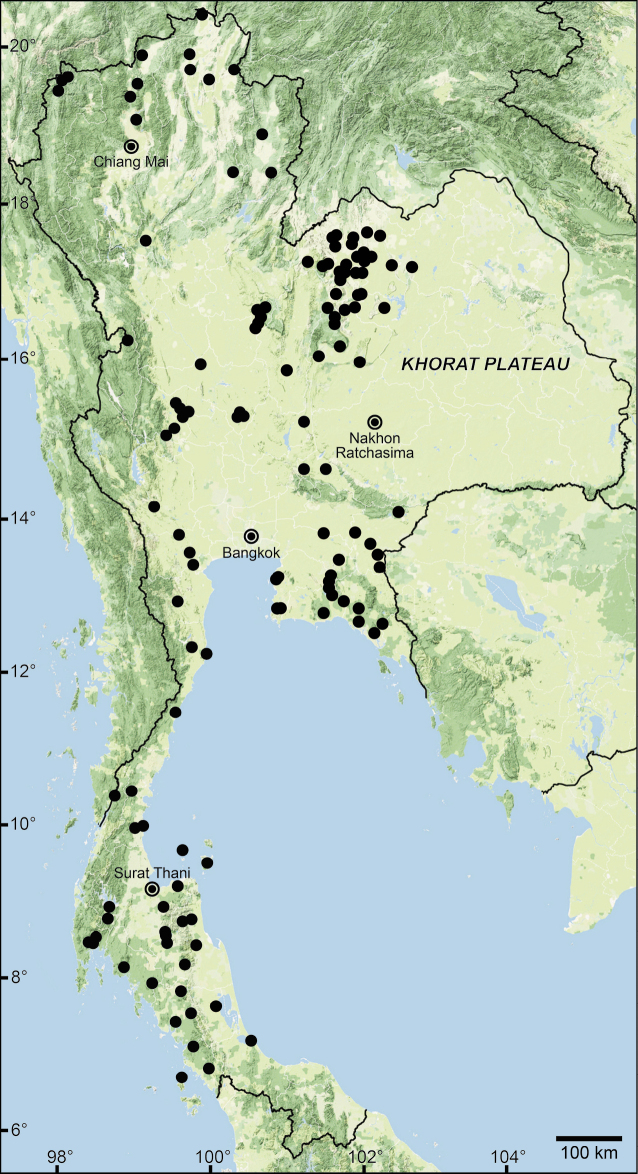
Sampling localities of the Pupinidae in Thailand from field surveys during 1995–2022.

**Figure 2.**
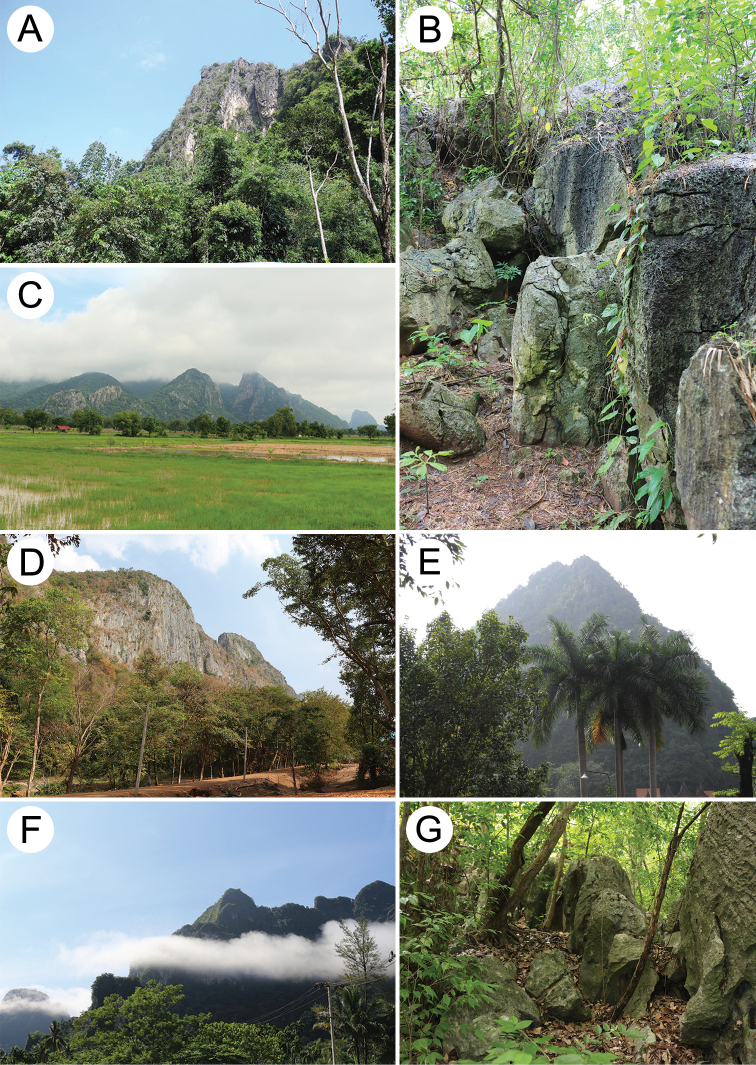
Habitat and vegetation around **A** Luang Cave, Chiang Rai, northern Thailand **B** Wang Daeng Cave, Phitsanulok, central Thailand **C** Tak Fa, Nakhon Sawan, central Thailand **D** Klong Had, Sra Keo, eastern Thailand **E** Khao Wong Cave, Uthai Thani, central Thailand **F** Phanom, Surat Thani, southern Thailand, and **G** Tham Khiriwong Temple, Prachub Kirikhan, western Thailand.

The data compiled in this checklist are from two main sources. The first source is the published malacological literature ranging from the nineteenth century until the present (February 2022). These historical works, i.e., the “Proceedings of the Zoological Society of London”, are available online at www.biodiversitylibrary.org and www.archive.org. This list includes all taxa in the family Pupinidae that have their type locality or subsequent localities reported from the area of “Siam” or present-day Thailand. The list also includes all *Pupina* and *Pupinella* species from mainland Southeast Asia, covering Cambodia, Laos, Myanmar, peninsular Malaysia, and Vietnam. The second source of information are field surveys conducted during 1995–2022 (Fig. 1). Land snails in Thailand were collected using direct search techniques throughout the country, including the northern mountainous forests, deciduous forests in the northeast, evergreen forests in the south, limestone areas throughout the country. Surveys included both anthropogenic and plantation areas (Fig. 2).

The direct searching for snails involved all potential land snail microhabitats that could be accessed, such as deep litter beds, decaying tree trunks, rock surfaces and crevices and, especially, limestone cliffs and caves. All sampled locations were recorded. At each locality, land snails were searched for intensively for ca. 1–2 h by three or four well-trained assistants. All living snails were photographed and killed by the two-step method for euthanasia (AVMA 2020) before being preserved in 70% ethanol for anatomical studies, or preserved in 95% (v/v) ethanol for molecular analyses. The handling of animals in this study was approved by Chulalongkorn University Animal Care and Use Committee (CU-ACUC) under the approval number 1723018. Empty shells were air dried in mesh bags for one to two weeks before being sorted. Intact adult shells were measured for whorl number, shell height, and major diameter or shell width using digital Vernier callipers (Mitutoyo, CD-6 CS). Shell spire angle was measured using a goniometer following Kozuch et al. (2017).

### ﻿Structure of the list

Species identification of specimens is based on the literature and comparisons with the type specimens and/or reference collections from several natural history museums. The classification of the higher taxa in the list is according to Bouchet et al. (2017) and the generic placements mainly follow Kobelt (1902), Clench (1949), Egorov (2013), Kongim et al. (2013), Páll-Gergely et al. (2014, 2015, 2017), Páll-Gergely and Grego (2019), Bui and Páll-Gergely (2020), and MolluscaBase (2022). Under each subfamily, the genera are listed alphabetically whereas the species within each genus are listed chronologically. Within each species or subspecies, the treatment includes the original combination of the taxon name with original spelling, and references to the page(s) and plate and/or figures. The type locality and the localities retrieved from past distribution records that address the occurrences of that particular taxon in Thailand are given verbatim as stated in that respective publication, and when possible, the modern name and/or regional name of those localities is provided in square brackets. In addition, when possible, the type materials with catalogue numbers, the images of the type specimens, and/or the images of newly collected specimens are also provided. Unless specified otherwise, all localities of CUMZ specimen lots are located in Thailand. The species which have an uncertain record from Thailand were not plotted in the distribution maps.

### ﻿Terminology of *Pupina* and *Pupinella* shells

**Figure 3.**
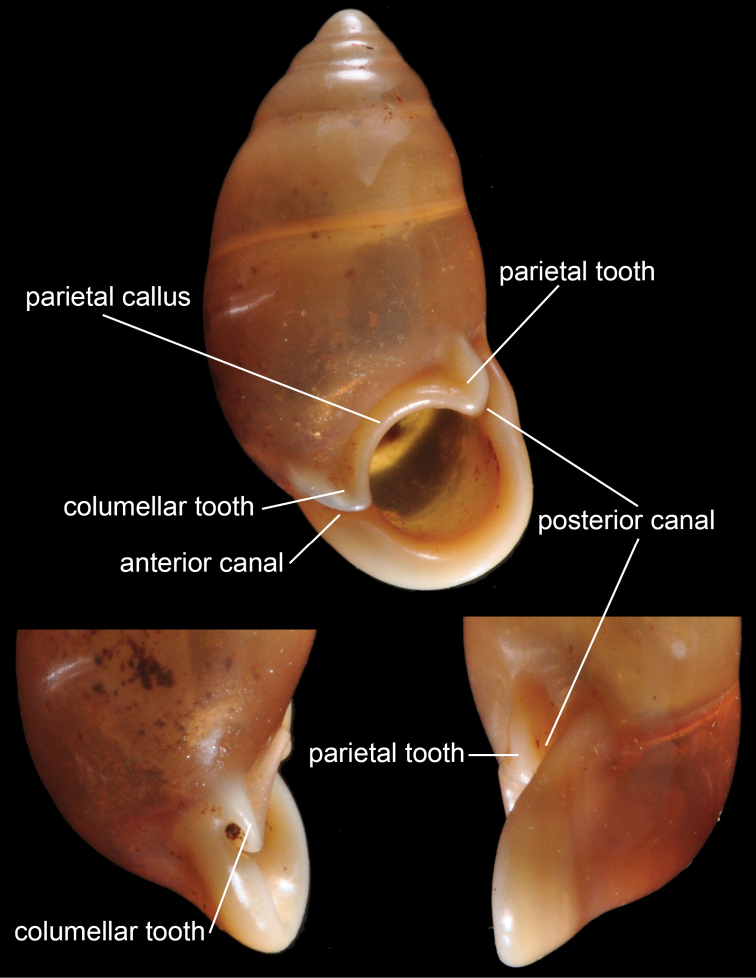
General shell morphology of *Pupina* and its terminology.

The terminology of teeth follows those of pupillid snails in Pilsbry (1918), where the upper tooth is called the parietal tooth and the lower tooth is called the columellar tooth (Fig. 3). For the terminology of canals, Egorov (2013) mentioned both ‘anterior’ and ‘posterior’ canals, and ‘columellar’ and ‘parietal’ canals. The anterior and posterior canals correspond to the columellar and parietal positions, respectively (Fig. 3). Here we adopt the terms ‘anterior’ and ‘posterior’ canals following the usages of Stanisic et al. (2010), Do (2017) and Tripathy and Sajan (2019). The terms ‘inner’ and ‘outer’ peristomes are adopted based on Liew et al. (2014: fig. 10) and Jirapatrasilp et al. (2021).

### ﻿Institutional abbreviations

**CUMZ** Chulalongkorn University Museum of Zoology, Bangkok;

**HNHM** Hungarian Natural History Museum, Budapest;

**HNUE** Museum of Biology of Hanoi National University of Education, Hanoi;

**MCZ** Museum of Comparative Zoology, Harvard University, Massachusetts;

**MNHN** Muséum national ďHistoire naturelle, Paris;

**NHMUK** when citing specimen lots deposited in the Natural History Museum, London (NHM);

**NMW** National Museum of Wales, Cardiff;

**NZSI** The National Zoological Collection of the Zoological Survey of India, Kolkata;

**RBINS** Royal Belgian Institute of Natural Sciences, Brussels;

**SMF** Forschungsinstitut und Naturmuseum Senckenberg, Frankfurt am Main;

**UMZC** Cambridge University Museum of Zoology, Cambridge;

**USNM** National Museum of Natural History, Smithsonian Institution, Washington, D.C.;

**ZRC** Zoological Reference Collection of Lee Kong Chian Natural History Museum, National University of Singapore.

### ﻿Other abbreviation

**amsl** above mean sea level.

### ﻿Photograph credits

Photographs of the type specimens from the Molluscs Collection (IM) of MNHN are credited to the museum taken under project E-RECOLNAT: ANR-11-INBS-0004 unless stated otherwise. Photographs of the type specimens and specimens from the other museum collections are credited to each respective museum.

### ﻿Taxon names

All the nominal species and subspecies names described as new to science in this work are attributed to the first author (Jirapatrasilp). Thus, a complete citation of the authors is “Jirapatrasilp in Jirapatrasilp et al., 2022”

## ﻿Results

A total of 195 voucher specimen lots was collected over the entire survey period and represented in this study. In total, 30 nominal species with two subspecies from seven genera are currently known to occur in Thailand. Two species of *Pseudopomatias*, and five species plus one subspecies of *Pupina* are described herein as new to science (Table 1). The taxonomic treatment of 15 *Pupina* species and three *Pupinella* species from mainland Southeast Asia are also included, together with the images of type specimen(s) where possible.

**Table 1.** List of species of the family Pupinidae recorded from Thailand.

Subfamily	Genus (and species group)	Species with recently collected material	Species not recently collected but based on literature and museum collections	Species with uncertain record from Thailand, based on literature only
Pupinellinae	*Coptocheilus*	*C.sectilabris*	*C.sumatranus*	–
	*Pollicaria*	*P.mouhotimonochroma*	–	*P.myersii*
		*P.mouhotimouhoti*		
	*Pseudopomatias*	*P.caligosus*	–	–
		*P.doiangkhangensis* Jirapatrasilp, sp. nov.		
		*P.pallgergelyi* Jirapatrasilp, sp. nov.		
	*Pupinella*	*P.mansuyi*	–	–
	*Rhaphaulus*	*R.lorraini*	*R.ascendens*	*R.chrysalis*
		*R.tonkinensis*	*R.perakensis*	
	*Tortulosa*	*T.tortuosa*	–	–
Pupininae	*Pupina*			
	*Pupinaartata* species group	*P.artata*	–	–
		*P.limitanea*		
		*P.pallens*		
		*P.bensoni* Jirapatrasilp, sp. nov.		
	*Pupinaarula* species group	*P.crosseana*	–	*P.arula*
		*P.peguensis*		*P.mouhoti*
		*P.siamensis*		
		*P.bilabiata* Jirapatrasilp, sp. nov.		
		*P.godwinausteni* Jirapatrasilp, sp. nov.		
	*Pupinaaureola* species group	*P.aureola*	–	–
		*P.paviei*		
		*P.tchehelensis*		
		*P.dorriisanensis* Jirapatrasilp, ssp. nov.		
		*P.latisulci* Jirapatrasilp, sp. nov.		
		*P.stoliczkai* Jirapatrasilp, sp. nov.		
Total	7	25	3	4

### ﻿Systematics

**Class Gastropoda Cuvier, 1795**

**Subclass Caenogastropoda Cox, 1960**

**Grade Architaenioglossa Haller, 1892**

#### Superfamily Cyclophoroidea Gray, 1847

##### ﻿Family Pupinidae

Taxon classificationAnimaliaArchitaenioglossaPupinidae

Pfeiffer, 1853

C8B50FFF-EB5B-5461-B215-A59710917724

###### Remarks.

Currently, there are three subfamilies within the family Pupinidae: Pupininae, Liareinae Powell, 1946, and Pupinellinae Kobelt, 1902 (Bouchet et al. 2017). The subfamily Liareinae was endemic to New Zealand, originally established as a family (Powell 1946), and this familial assignment was adopted by Egorov (2013). Later, Ponder and Warén (1988) treated this taxon as a subfamily of the Pupinidae; this classification scheme was adopted by Bouchet et al. (2017) and MolluscaBase (2022).

The subfamily Pupinellinae was originally established as a section under the Pupinidae, and the only diagnostic character that distinguished this subfamily from the Pupininae is the shell surface (Kobelt 1902). The Pupininae has a shell surface covered by glaze, which is glossy and completely smooth, whereas the shell surface of the Pupinellinae is without glaze, being either striated, matt or silky-shiny (Kobelt 1902; Egorov 2013). Whether this character is a subfamilial synapomorphy needs further confirmation because at least one *Pupina* species has a matt surface (e.g., *P.arula*) and some *Pupinella* species have a somewhat glossy surface (e.g., *P.mansuyi*, *P.illustris*).

##### ﻿Subfamily Pupinellinae

Taxon classificationAnimaliaArchitaenioglossaPupinidae

Kobelt, 1902

73AA3B96-DC21-546D-B4A3-D8F200329C2E

###### Remarks.

There are a total of six genera with 12 species and one subspecies of pupinellinid known to occur in Thailand, and two additional species have uncertain records.

##### ﻿1. Genus Coptocheilus

Taxon classificationAnimaliaArchitaenioglossaPupinidae

Gould, 1862

59AB69FA-7E4E-58C1-A6E1-4FC0568D7741

Coptocheilus Gould, 1862: 282.
Schistoloma Kobelt, 1902: 278. Egorov 2013: 14.

###### Type species.

*Cyclostomaaltum* Sowerby I, 1842, by original designation.

###### Diagnosis.

Shell high conical to elongate ovate. Sculpture mostly smooth, rarely ribbed. Periumbilical keel either present or absent. Aperture round without any tubes or apparent slits, sometimes with a slight angular indentation at peristome upper junction. Operculum thin, flat, closely coiled.

###### Differential diagnosis.

Shell size and matt surface of *Coptocheilus* are more similar to *Tortulosa* than other genera in this subfamily. However, *Coptocheilus* is different from *Tortulosa* in having a round aperture without any tubes or apparent slits, but sometimes with a slight angular indentation at the upper junction of peristome. In addition, *Coptocheilus* has a thin, flat operculum, and does not have a periumbilical keel (Kobelt 1902).

###### Remarks.

**Figure 4.**
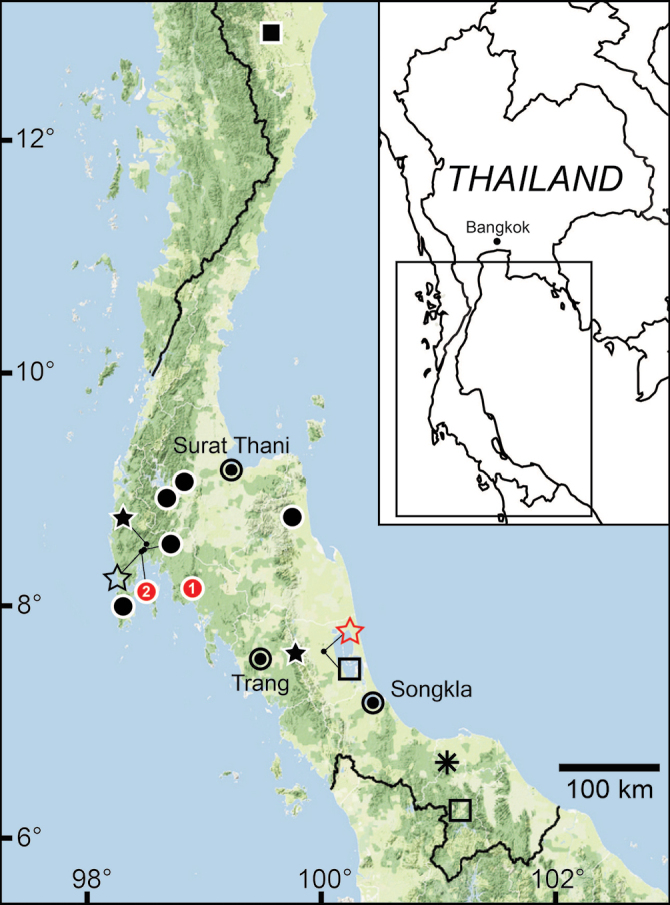
Map of southern Thailand showing the distribution of *Coptocheilussectilabris* (filled square), *Coptocheilussumatranus* (open square), *Rhaphauluslorraini* (filled star), *Rhaphaulusascendens* (open star), *Rhaphaulusperakensis* (asterisk), and *Tortulosatortuosa* (circle). Each red symbol indicates the type locality of its respective taxon. Red circles indicate the type localities of *Tortulosahuberi* (1) and *Tortulosaschileykoi* (2).

For the resurrection of *Coptocheilus* Gould, 1862 over *Schistoloma* Kobelt, 1902 and the list of all *Coptocheilus* species, see Bui and Páll-Gergely (2020). The distribution of *Coptocheilus* species in Thailand is provided in Fig. 4.

##### ﻿ Coptocheilus sectilabris

Taxon classificationAnimaliaArchitaenioglossaPupinidae

(Gould, 1843)

75A322CF-5929-5FB2-B958-38ADA5B9D075

Fig. 5A–C

Cyclostoma sectilabrum Gould, 1843: 140. Type locality: Tavoy [Dawei, Dawei Township, Dawei District, Tanintharyi Region, Myanmar]. Gould 1844: 459, pl. 24, fig. 10. Pfeiffer 1848: pl. 24, figs 17, 18. Pfeiffer 1849: 164, 165. Johnson 1964: 147.
Megalomastoma sectilabre —Theobald 1858[1857]: 247, Yanglaw, on the Tenasserim [Tanintharyi Region, Myanmar].
Megalomastoma sectilabrum —Sowerby I 1866: Pupinidae, pl. 1 (pl. 263), Pollicaria and Megalomastoma, sp. 19, fig. 24. Hanley and Theobald 1870: 4, pl. 7, fig. 3. Reeve 1878: Pupinidae, pl. 10, sp. 88. Crosse 1879: 339. de Morgan 1885: 412, 413.
Megalomastoma (Coptocheilus) sectilabrum —Nevill 1878: 297.
Megalomastoma (Coptochilus) sectilabrum —von Martens 1886: 161, King Island [Kadan Island or Kadan Kyun, Kyunsu Township, Myeik District, Tanintharyi Region, Myanmar]. von Möllendorff 1887[1886]: 314, Tenasserim.
Schistoloma sectilabrum —Kobelt 1902: 280. Gude 1921: 170, 171. Zilch 1957: 42. Maassen 2001: 43. Tumpeesuwan and Panha 2008: 65, 66, fig. 1a–c, Kaeng Krachan National Park, Phetchaburi Province, Thailand. Egorov 2013: 14, fig. 22d–g. BEDO 2017: 97. Sutcharit et al. 2018: 157, figs 5–11e, 5–13m. Páll-Gergely et al. 2019: 325, 326.

###### Type material examined.

**Figure 5.**
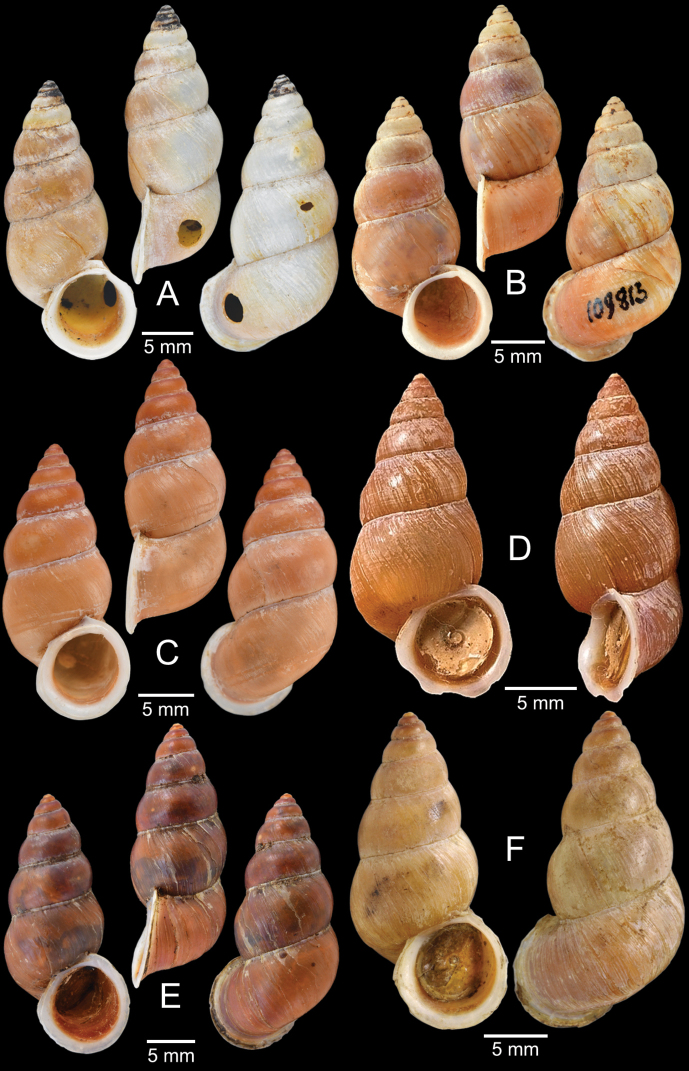
**A–C***Coptocheilussectilabris*: **A** lectotype MCZ 169361 from Tavoy **B** specimen SMF 109813 from Tavoy, and **C** specimen CUMZ OLM-0111 from Kaeng Krachan, Phetchaburi **D–F***Coptocheilussumatranus*: **D** syntype of *Coptocheilusperakensis*NHMUK 1903.11.20.33 from Perak **E** specimen SMF 262529/1 “*Schistolomasiamensis* Brandt” from Thailand: an den Tanto-Fällen bei Ban Nong Star; Yala Provinz, and **F** specimen NHMUK 1986.4.19.14 “*Coptocheilussectilabrum* var.” from Larut near Perak.

***Lectotype***MCZ 169361 (Fig. 5A) from Tavoy. Paralectotype MCZ 87934 (1 shell) from Tavoy.

###### Other material examined.

SMF 109813 (1 shell; Fig. 5B) from Tavoy. CUMZ OLM-0111 (1 shell; Fig. 5C) from Kaeng Krachan District, Phetchaburi Province, 20 Sept. 1998.

###### Diagnosis.

Shell elongate conical without any periumbilical keel. Aperture round with a slight angular indentation at upper junction of peristome.

###### Differential diagnosis.

*Coptocheilussectilabris* is different from *C.sumatranus* in having a slight angular indentation at the upper junction of the peristome.

###### Distribution.

Myanmar and western Thailand (Tumpeesuwan and Panha 2008).

###### Remarks.

As the original description did not explicitly state that the description of this species was based on a single specimen (nor could this be inferred), the designation of a holotype by Johnson (1964) in fact constitutes a lectotype designation (ICZN 1999: Art. 74.6). Several records of *C.sectilabris* from southern Thailand and peninsular Malaysia should be recognised as *C.sumatranus* (see below). The occurrence of *C.sectilabris* in Vietnam (Thach 2016) is dubious and needs further confirmation (Páll-Gergely et al. 2019).

##### ﻿ Coptocheilus sumatranus

Taxon classificationAnimaliaArchitaenioglossaPupinidae

Dohrn, 1881

741DB97B-8656-55FE-AA24-2485FF24F296

Fig. 5D–F

Coptocheilus sumatranus Dohrn, 1881: 65. Type locality: Sumatra, Singalang [Mount Singgalang, West Sumatra].
Megalomastoma sectilabrum [in part]—Stoliczka 1872: 268, pl. 10, fig. 13, Penang hill [Penang Island, Penang State, Malaysia]. Crosse 1879: 339, Perak [Malaysia]. de Morgan 1885: 412, 413.
Megalomastoma (Coptocheilus) sectilabrum [in part]—Nevill 1878: 297.
Megalomastoma (Coptochilus) sectilabrum [in part]—von Martens 1886: 161. von Möllendorff 1887[1886]: 314; Larut [Bukit Larut, Perak State, Malaysia].
Coptochilus sectilabrum [non Gould]— von Möllendorff 1891: 346.
Schistoloma sectilabrum [in part]—Kobelt 1902: 280. Gude 1921: 170, 171.
Coptocheilus perakensis Fulton, 1903: 102, pl. 9, fig. 3. Type locality: Perak.
Schistoloma perakense —Laidlaw 1928: 33.
Schistoloma sectilabrum [non Gould]—Sykes 1903: 197, Ulu Selama, Perak. Laidlaw 1928: 33, Ulu Selama, Perak; Lampan Patalung [Phatthalung Province, Thailand]. Foon et al. 2017: 41, fig. 16b, Perak, forested slope behind the village at Gunung Pondok.
Schistoloma sumatranum —Kobelt 1902: 281. van Benthem Jutting 1949: 55, 56, Kuala Legap, Plus Valley, Perak; Maxwell’s Hill, Perak; Gunong Kledang, Perak; Taiping Perak; Dusun Tua, Selangor [Malaysia]. Davison 1995: 236, Sungai Halong and Sungai Emban, Temengor Forest Reserve, Perak, Malaysia. Chan 1998a: 4, Ipoh, Perak. Maassen 2001: 43, 44. Páll-Gergely et al. 2019: 327.
Schistoloma perakensis —Berry 1963: pl. 6, fig. 29.

###### Type material examined.

***Syntype*** of *Coptocheilusperakensis*NHMUK 1903.11.20.33 (1 shell; Fig. 5D) from Perak.

###### Other material examined.

SMF 262529/1 “*Schistolomasiamensis* Brandt” (1 shell; Fig. 5E) from Thailand: an den Tanto-Fällen bei Ban Nong Star; Yala Provinz [Than To Waterfall Forest Park, Bannang Sata District, Yala Province, Thailand]. NHMUK 1986.4.19.14 “*Coptocheilussectilabrum* var.” (1 shell; Fig. 5F) from Larut near Perak.

###### Diagnosis.

Shell elongate conical without any periumbilical keel. Apertural round without any indentation.

###### Differential diagnosis.

*Coptocheilussumatranus* is different from *C.sectilabris* in having a round aperture without any indentation.

###### Distribution.

Peninsular Malaysia, Sumatra Island, and southern Thailand (Laidlaw 1928; van Benthem Jutting 1949; Foon et al. 2017).

###### Remarks.

No material of this species was found during this survey. Although *C.sumatranus* only differs from *C.sectilabris* by an absence of an indentation in the peristome (van Benthem Jutting 1949), we do not synonymise *C.sumatranus* with *C.sectilabris* because of the lack of DNA data and that there are too few specimens to verify whether specimens collected from the same localities of *C.sectilabris* eventually lack an angular indentation in the peristome. *Coptocheilusperakensis* was retrieved as a junior subjective synonym of *C.sumatranus* because there are no distinct differences in shell form and size between them (*C.sumatranus*: shell height 19–24 mm, diameter 8–9 mm; *C.perakensis*: shell height 23 mm, diameter including peristome 11 mm; van Benthem Jutting 1949).

The name “*Schistolomasiamensis* Brandt” given to two samples (SMF 262529 = holotype” and SMF 262530 = “paratypes”) from Than To Waterfall Forest Park, Bannang Sata District, Yala Province, Thailand was never published and so is not available. These specimens are larger, more elongated, and have a darker shell colour but the other diagnostic characters conform to those found in the syntype of ‘*C.perakensis*’. Thus, Brandt’s specimens are herein identified as *C.sumatranus*.

##### ﻿2. Genus Pollicaria

Taxon classificationAnimaliaArchitaenioglossaPupinidae

Gould, 1856

6C24ED5F-B355-5963-B69C-50715E6F6F48

Pollicaria Gould, 1856: 14. Kobelt 1902: 288, 289. Egorov 2013: 15, 16.

###### Type species.

*Cyclostomapollex* Gould, 1856 (junior synonym of *Megalomastomagravidum* Benson, 1856), by monotypy.

###### Diagnosis.

Shell of great size (up to 50 mm in shell height); pupoid shape with shallow posterior angled groove at palatal edge as breathing device; with or without parietal declining shoulder inside the peristome.

###### Differential diagnosis.

*Pollicaria* can be distinguished from all other genera in this subfamily by a greater shell size, and a shallow posterior angled groove at palatal edge as a breathing device (Kongim et al. 2013; Minton et al. 2017).

###### Remarks.

**Figure 6.**
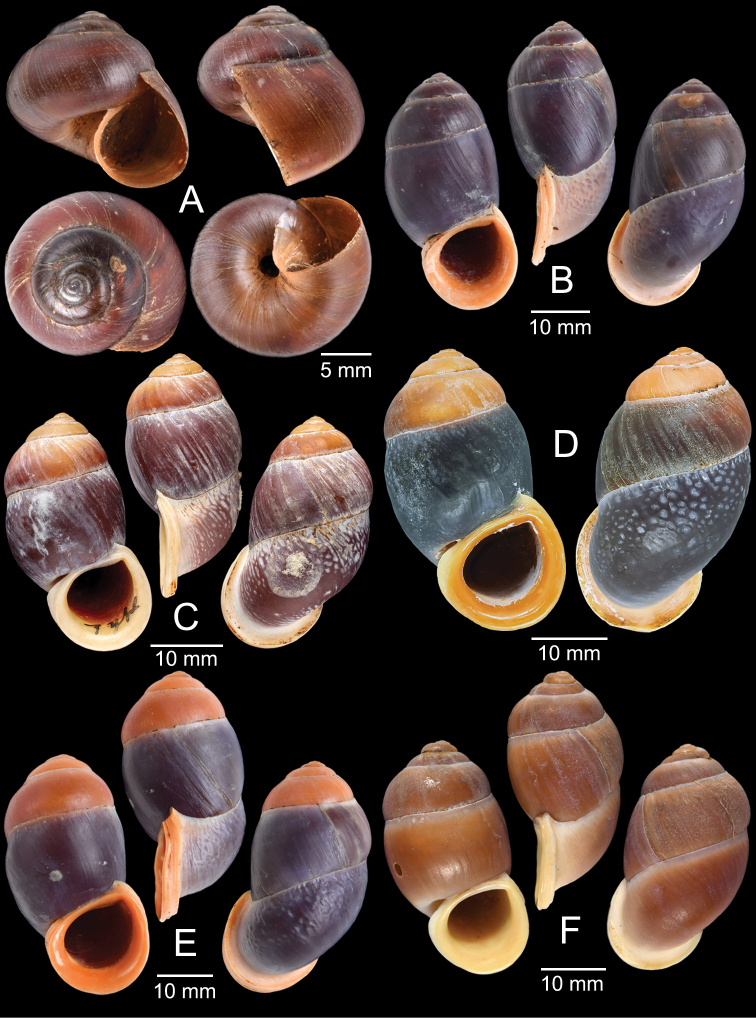
**A, B***Pollicariamouhotimonochroma*: **A** juvenile specimen CUMZ 12182 from Tham Suea Lueang Temple, Loei and **B** paratype CUMZ 1562 from Tam Pha Bing Temple, Loei **C–E***Pollicariamouhotimouhoti***C** lectotype of *Hybocystismouhoti*NHMUK 20130071/1 from Lao Mountains, Camboja **D** holotype of ‘*Pollicarianicoarlingi*’ MNHN-IM-2000-37277, and **E** specimen CUMZ 12166 from Wang Daeng Cave, Phitsanulok **F***Pollicariamyersii*, holotype of ‘*Pollicariahuberi*’ NHMUK 20180253. Photo: F. Prugnaud, MNHN (**D**).

**Figure 7.**
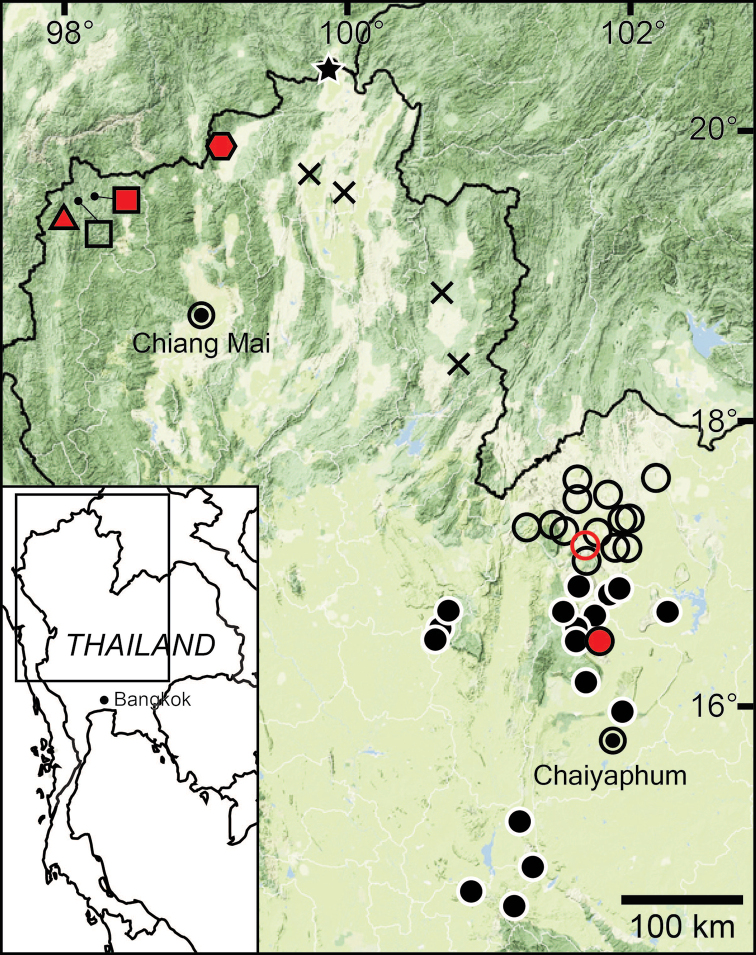
Map of northern Thailand showing the distribution of *Pollicariamouhotimouhoti* (filled circle), *Pollicariamouhotimonochroma* (open circle), *Pseudopomatiascaligosus* (square), *Pseudopomatiasdoiangkhangensis* sp. nov. (hexagon), *Pseudopomatiaspallgergelyi* sp. nov. (triangle), *Pupinellamansuyi* (cross), and *Rhaphaulustonkinensis* (star). Each red symbol indicates the type locality of its respective taxon. The red filled circle denotes the type locality of *Pollicarianicoarlingi*.

The taxonomic history of *Pollicaria* was reviewed in Kongim et al. (2013) and Minton et al. (2017). The juvenile shell of this genus (Fig. 6A) does not develop the large last whorl seen in adults (Fig. 6B), making its shell shape similar to the pulmonated ariophantid snails, which might lead to a misidentification [see the case of *Ariophantahuberi* Thach, 2018 and *P.rochebruni* (Mabille, 1887) in Páll-Gergely and Hunyadi (2018a)]. The distribution of *Pollicaria* species in Thailand from Kongim et al. (2013) and this study is provided in Fig. 7.

##### ﻿ Pollicaria mouhoti mouhoti

Taxon classificationAnimaliaArchitaenioglossaPupinidae

(Pfeiffer, 1863)

54F3738F-70B2-5E87-89E5-F2F4A63DB027

Figs 6C–E , 8A, B

Hybocystis mouhoti Pfeiffer, 1863b [1862]: 276, pl. 36, fig. 13. Type locality: Lao Mountains, Camboja [Cambodia or Laos]. Pfeiffer 1863a: 227, 228, pl. 59, figs 5–8. Nevill 1878: 298, Siam (?). Fischer 1891: 108. Fischer and Dautzenberg 1904: 432.
Pollicaria mouhoti —Sowerby I 1866: Pupinidae, pl. 1 (pl. 263), Pollicaria and Megalomastoma, sp. 3, fig. 9. Reeve 1878: Pupinidae, pl. 8, sp. 67. Sutcharit et al. 2018: 156, figs 5–11c, 5–12a–g, 5–13a. Inkhavilay et al. 2019: 28, fig. 15a, Thailand, probably in both Cambodia and Laos.
Megalomastoma (Hybocystis) mouhoti — von Martens 1867: 67.
Pollicaria myersii [non Haines]—Habe 1965: 114, 115, pl. 2, fig. 3, Phukae Botanical Garden, Sara Buri [Province], Thailand (limestone region).
Pollicaria mouhoti mouhoti —Kongim et al. 2013: 31, 32, figs 2b, 3a–e, 4h, i, 6b. BEDO 2017: 86. Thach 2018: 96 (figure caption), figs 124, 125.
Pollicaria nicoarlingi Thach, 2021: 17, 18, figs 53–55, 57, 58. Type locality: Konsan District, Chaiyaphum Province, Thailand. Syn. nov.

###### Type material examined.

***Lectotype*** of *Hybocystismouhoti*NHMUK 20130071/1 (Fig. 6C) and paralectotypes NHMUK 20130071/2–3 (2 shells) from Lao Mountains, Camboja. ***Holotype*** of *Pollicarianicoarlingi*MNHN-IM-2000-37277 (Fig. 6D) from Konsan District, Chaiyaphum Province, Thailand.

###### Other material examined.

**Figure 8.**
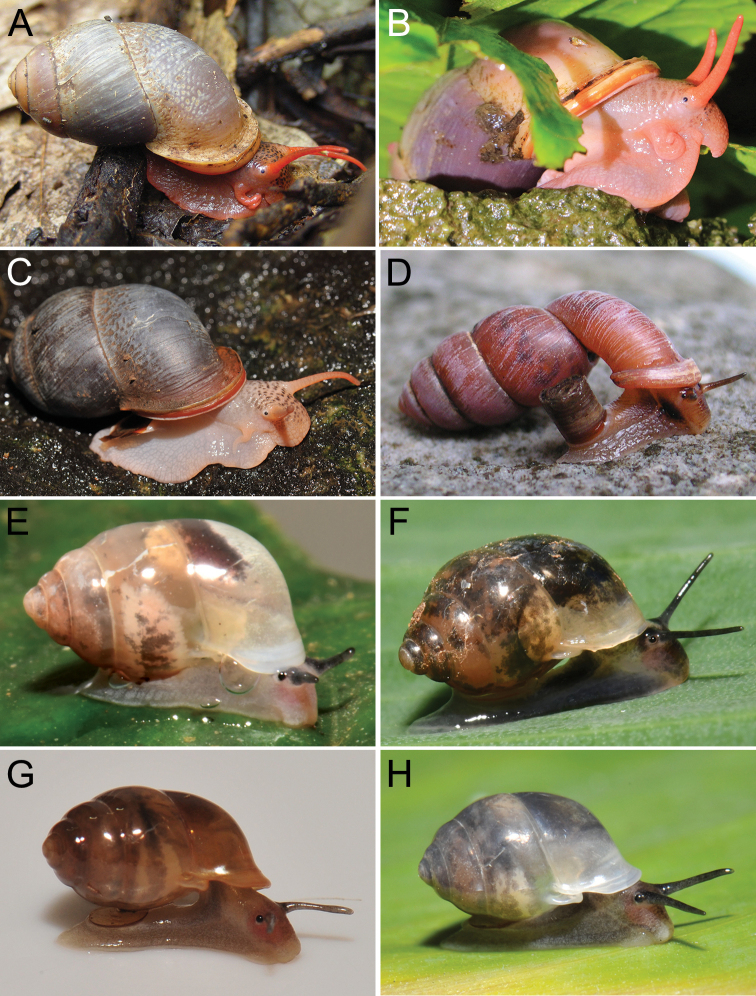
Live specimens of **A, B***Pollicariamouhotimouhoti*: specimens **A**CUMZ 12166 and **B**CUMZ 12175 from Wang Daeng Cave, Phitsanulok **C***Pollicariamouhotimonochroma*, paratype CUMZ 1562 from Tam Pha Bing Temple, Loei **D***Tortulosatortuosa*, specimen CUMZ 12155 from Tham Suea Temple, Krabi **E–H***Pupinaartata*: specimens of **E**CUMZ 12006 from Pha Daeng Cave, Mae Hong Son **F**CUMZ 12008 from Tham Nam Pha Pha Ngam Temple, Lampang, and **G, H**CUMZ 12029 from Khao Tham Raet Temple, Chachoengsao showing the brown (**G**) and grey (**H**) shell morphs; All not to scale.

CUMZ 12166 (5 shells and 5 specimens in ethanol; Figs 6E, 8A) from Wang Daeng Cave, Noen Maprang District, Phitsanulok Province, 17 Mar. 2017. CUMZ 12175 (3 specimens in ethanol; Fig. 8B) from Wang Daeng Cave, Noen Maprang District, Phitsanulok Province, 8 June 2017. CUMZ 12176 (6 adult shells and 1 juvenile shell) from Phu Wiang District, Khon Kaen Province, 8 July 1995. CUMZ 12177 (1 shell) from Phraya Nakkharaj Cave, Chum Phae District, Khon Kaen Province, 21 July 2020. CUMZ 12178 (8 shells and 4 specimens in ethanol) from Tad Tone Waterfall, Mueang Chaiyaphum District, Chaiyaphum Province, 20 July 2020. CUMZ 12179 (9 shells) from Pa Mamuang Bureau of Monks, Noen Maprang District, Phitsanulok Province, 3 Aug. 2020. CUMZ 12180 (1 shell) from Tham Phrommalok Temple, Chai Badan District, Lopburi Province, 24 Aug. 2020. CUMZ 12181 (1 shell) from Tham Badan Temple, Muak Lek District, Saraburi Province, 3 Aug. 2020.

###### Diagnosis.

Shell height 35–40 mm. Last whorl and penultimate whorl purple to black; spire and apex distinct yellow to bright orange. Dorsal side of last whorl with bold wrinkles. Aperture round, without apertural groove; apertural lip expanded, bright orange to red. Umbilicus subumbilicate.

###### Differential diagnosis.

*Pollicariamouhotimouhoti* is similar to *P.myersii* and *P.m.monochroma* in shell shape, but different from *P.myersii* by a smaller shell size with purplish shell colour, bright orange spire, expanded bright orange to red apertural lip and bold wrinkles on the dorsal side of last whorl, and different from *P.m.monochroma* by a larger shell size, yellow to bright orange spire and apex, and a distinct karyotype pattern of (6m+4sm+2st+1t) (Kongim et al. 2009, 2010, 2013).

###### Distribution.

Phetchabun Range in central and northeastern Thailand, and probably in both Cambodia and Laos (Kongim et al. 2013; Inkhavilay et al. 2019).

###### Remarks.

Pain (1974) treated *P.mouhoti* as a subjective synonym of *P.myersii*, whereas Kongim et al. (2013) regarded *P.mouhoti* as valid because these two species are distinct in several shell characters and karyotype pattern. Thus, the distribution range of *P.myersii* is restricted to limestone areas of Vientiane to Luang Prabang, Laos, and probably to the northern part of Thailand, whereas *P.mouhoti* mostly occurs in central and northeastern Thailand (Kongim et al. 2013).

One differential diagnostic character of *P.nicoarlingi* is “special sculpture with many large, broad, and deep holes on dorsal side” (Thach 2021). This character is not unique because all the type specimens and recently collected specimens of *P.m.mouhoti* have this kind of shell sculpture, although to a different degree. The “special colour” of a very red columellar outer lip and parietal wall, and an orange spire and apex of *P.nicoarlingi* conform to the type specimens of *P.m.mouhoti*, although there is variation in the spire and apex colour from dark brown to bright orange. Other differences in shell shape, apertural lip, columella and sculpture of umbilicus between *P.nicoarlingi* and *P.m.mouhoti* as stated by Thach (2021) are possibly due to different shell condition and infraspecific variation. Moreover, *P.nicoarlingi* is described from the same vicinity of *P.m.mouhoti* specimens examined in this study. Therefore, *P.nicoarlingi* is regarded herein as a junior subjective synonym of *P.m.mouhoti*.

##### ﻿ Pollicaria mouhoti monochroma

Taxon classificationAnimaliaArchitaenioglossaPupinidae

Kongim & Panha, 2013

52F187EB-B7B8-5E5E-80BC-4DC4905C5FF4

Figs 6A, B , 8C

Pollicaria myersii [non Haines]—Solem 1966: 13, on limestone outcrops 20 km. east of Wang Sapung [District] near Loei [Province], Thailand.
Pollicaria mouhoti monochroma Kongim & Panha in Kongim et al. 2013: 32, 33, figs 2c, 4j, k, 6c. Type locality: limestone outcrop with dry forest at Wat Tam Pha Bing, Wungsapoong District, Loei Province, Thailand. BEDO 2017: 86. Sutcharit et al. 2018: 156.

###### Type material examined.

***Holotype***CUMZ 1577 and ***paratypes***CUMZ 1548 (9 shells) figured in Kongim et al. (2013: figs 4j, k). ***Paratypes***CUMZ 1562 (85 shells and 10 specimens in ethanol; Figs 6B, 8C) from Tam Pha Bing Temple, Wungsapoong District, Loei Province, 11 June 2013.

###### Other material examined.

CUMZ 12182 (3 juvenile shells; Fig. 6A) from Tham Suea Lueang Temple, Mueang Loei District, Loei Province, 1 Sept. 2020. CUMZ 12183 (4 shells) from Tham Pha Poo, Mueang Loei District, Loei Province, 1 Sept. 2020. CUMZ 12184 (3 adult shells and 2 juvenile shells) from Phu Pha Lom, Mueang Loei District, Loei Province, 1 Sept. 2020. CUMZ 12185 (3 adult shells and 7 juvenile shells) from Tham Pha Phung Temple, Wang Saphung District, Loei Province, 2 Sept. 2020. CUMZ 12186 (3 adult shells and 3 juvenile shells) from Pa Phaya Temple, Suwannakhuha District, Nong Bua Lam Phu Province, 31 Aug. 2020.

###### Diagnosis.

Shell height < 35 mm. Shell entirely black to purple. Dorsal side of last whorl with bold wrinkles. Aperture almost round, shallow posterior angled groove present; apertural lip expanded, yellow to pale orange. Umbilicus narrow.

###### Differential diagnosis.

This subspecies is different from the nominotypical subspecies by a smaller shell size, an entirely black to purple shell, and a distinct karyotype pattern of (7m+3sm+2st+1t) (Kongim et al. 2009, 2013).

###### Distribution.

Loei and Nong Bua Lam Phu provinces, northeastern Thailand (Kongim et al. 2013).

###### Remarks.

DNA data are required to demonstrate whether *P.m.monochroma* is distinct from the nominotypical subspecies and should be elevated to specific status.

#### Species with uncertain record from Thailand

##### ﻿ Pollicaria myersii

Taxon classificationAnimaliaArchitaenioglossaPupinidae

(Haines, 1855)

4A2D0679-3FED-5978-9F9E-EB5FAF9B54EA

Fig. 6F

Cyclostoma myersii Haines, 1855: 157, pl. 5, figs 9–11. Type locality: Siam [Thailand].
Pollicaria myersi [sic]—Sowerby I 1866: Pupinidae, pl. 1 (pl. 263), Pollicaria and Megalomastoma, sp. 2, fig. 11. von Martens 1867: 67. Reeve 1878: Pupinidae, pl. 8, sp. 69.
Hybocystis myersi [sic]—Fischer 1891: 108. Fischer and Dautzenberg 1904: 432.
Pollicaria myersii —Pain 1974: 175, 176, pl. 6, figs 2, 5. Hemmen and Hemmen 2001: 39. Kongim et al. 2013: 30, figs 2a, 4f, g, 6a, limestone areas of Vientiane to Luang Prabang, Laos, and probably the northern part of Thailand. BEDO 2017: 87. Sutcharit et al. 2018: 156, fig. 5–13b. Inkhavilay et al. 2019: 28, figs 15b, 18g, Ban Phone Can village, Yommalath District, Khammouan Province, Laos. Páll-Gergely et al. 2020: 40.
Pollicaria huberi Thach, 2018: 20, 21, figs 116–123. Type locality: Thakhek, Laos.

###### Type material examined.

***Holotype*** of *Pollicariahuberi*NHMUK 20180253 (Fig. 6F) from Thakhek, Laos.

###### Other material examined.

NHMUK 20090242 from Siam figured in Kongim et al. (2013: fig. 4f). CUMZ 1531, 1572 figured in Kongim et al. (2013: fig. 4g), 1591 from Pahom, Vang Vieng, Laos.

###### Diagnosis.

Shell height > 40 mm. Shell elongated, reddish brown to bright orange or red. Dorsal side of last whorl with very fine wrinkles. Aperture round, without apertural groove; apertural lip expanded, yellow to pale orange. Umbilicus narrow.

###### Differential diagnosis.

*Pollicariamyersii* is different from *P.m.mouhoti* by having an elongated purple to pale orange shell with thin periostracum, a rounded aperture, very fine wrinkles on the dorsal part of the last whorl, and a distinct karyotype pattern of (4m+6sm+2st+1t). This species also differs from *P.gravida*, *P.rochebruni* and *P.crossei* by having a larger shell, no apertural groove, and noticeable wrinkles on last whorl (Kongim et al. 2010, 2013).

###### Distribution.

Laos and an uncertain record from northern Thailand (Kongim et al. 2013; Inkhavilay et al. 2019).

###### Remarks.

No material of this species was found during this survey, and the record in Thailand needs further confirmation. The type material of this species was presumably lost (Kongim et al. 2013). Páll-Gergely et al. (2020) treated *P.huberi* as a junior subjective synonym of *P.myersii* because the shell shape and colour, and the aperture shape of *P.huberi* agree with those of *P.myersii*, which also occurs in Laos.

##### ﻿3. Genus Pseudopomatias

Taxon classificationAnimaliaArchitaenioglossaPupinidae

Möllendorff, 1885

3A1F0DD6-16CE-5CE7-83E1-458C0091F975

Pseudopomatias Möllendorff, 1885: 164. Kobelt 1902: 272. Egorov 2013: 12.

###### Type species.

*Pseudopomatiasamoenus* Möllendorff, 1885, by monotypy.

###### Diagnosis.

Shell turriform or spindle-shaped, rather regularly ribbed, without additional groove above the suture, and without basal keel. Aperture rather round with slight columellar-parietal and more angled parietal-palatal transitions.

###### Differential diagnosis.

*Pseudopomatias* is similar to *Hedleya* Cox, 1892, *Nodopomatias* Gude, 1921, *Vargapupa* Páll-Gergely, 2015 and *Csomapupa* Páll-Gergely, 2015 in shell shape and ribbing, but different from *Hedleya* by an absence of two canals in the aperture, different from *Nodopomatias* and *Vargapupa* by an absence of a basal keel, and different from *Csomapupa* by the lack of an additional line (groove) above the suture (Páll-Gergely et al. 2015).

###### Remarks.

The taxonomic history of *Pseudopomatias* was reviewed and its systematic position in the family Pupinidae was confirmed by Páll-Gergely et al. (2015). The distribution of all *Pseudopomatias* species in Thailand is provided in Fig. 7.

##### ﻿ Pseudopomatias caligosus

Taxon classificationAnimaliaArchitaenioglossaPupinidae

Páll-Gergely & Hunyadi, 2018

FEE7EEE3-71A4-59AE-B33C-6CDD2938282C

Fig. 9A, B

Pseudopomatias caligosus Páll-Gergely & Hunyadi, 2018b: 64, fig. 3. Type locality: Mae Hong Son Province, 9.1 km from Ban Soppong towards Mae Hong Son, left side of road # 1095, Thailand. Páll-Gergely and Grego 2019: 588, fig. 2a–h, 169.5 km milestone, 36 km west towards Taungoo, Demoso, Kayah State, Myanmar.

###### Type material examined.

**Figure 9.**
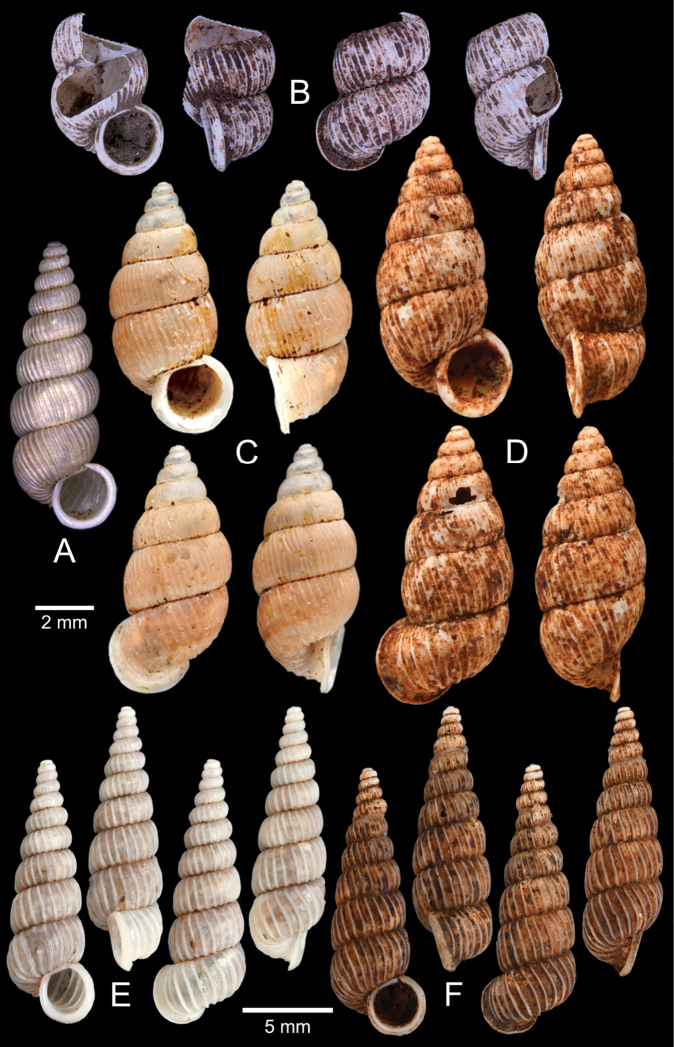
**A, B***Pseudopomatiascaligosus*: **A** holotype HNHM 100176 and **B** specimen CUMZ 12191 from Pa Tham Wua Temple, Mae Hong Son **C, D***Pseudopomatiasdoiangkhangensis* sp. nov. **C** holotype CUMZ 12165/1 and **D** paratype CUMZ 5219 from Doi Ang Khang, Chiang Mai **E, F***Pseudopomatiaspallgergelyi* sp. nov. **E** holotype CUMZ 12167/1 **F** paratype CUMZ 12167/2 from Pha Daeng Cave, Mae Hong Son. Photo: B. Páll-Gergely (**A**).

***Holotype***HNHM 100176 (Fig. 9A) and ***paratypes***HNHM 100442 (17 shells) from the type locality.

###### Other material examined.

CUMZ 12191 (1 shell; Fig. 9B) from Pa Tham Wua Temple, Mueang Mae Hong Son District, Mae Hong Son Province, 18 Jan. 2015.

###### Diagnosis.

Shell slender turriform; ca. 9 whorls, with regular strong ribs. Area between ribs with very fine spiral striation mostly on upper whorls. Peristome reflected.

###### Differential diagnosis.

*Pseudopomatiascaligosus* is most similar to *P.peguensis* (Theobald, 1864) and *P.shanensis* Páll-Gergely, 2015 in shell size and bulging whorls, but different from *P.peguensis* by a less glossy shell, much stronger ribs, and a reflected peristome, and different from *P.shanensis* by more bulging whorls, a less expanded peristome, and less-packed ribs with indistinct spiral striation between them (Páll-Gergely et al. 2015; Páll-Gergely and Hunyadi 2018b).

###### Distribution.

Mae Hong Son Province and Kayah State, Myanmar (Páll-Gergely and Hunyadi 2018b; Páll-Gergely and Grego 2019).

###### Remarks.

Although the apex of the CUMZ specimen is broken, the other remaining characters conform to those of the holotype of *P.caligosus*. The collecting locality is in the same vicinity as the type locality.

##### ﻿ Pseudopomatias doiangkhangensis

Taxon classificationAnimaliaArchitaenioglossaPupinidae

Jirapatrasilp sp. nov.

9145912A-B3D0-52F8-8DE6-A0D08BD4AFBC

https://zoobank.org/C419E00F-438D-4A5C-BC61-8AD63F0828E0

Fig. 9C, D

###### Type material.

***Holotype***CUMZ 12165/1 (Fig. 9C), 24 Oct. 2015, coll. C. Sutcharit, R. Srisonchai, A. Pholyotha, T. Seesamut. Measurement: shell height 8.6 mm, shell width 4.3 mm and 7½ whorls. ***Paratypes***CUMZ 12165/2–6 (5 shells), NHMUK 20210331 (2 shells), same data as holotype; CUMZ 5219, 5221, 16 Mar. 2000, coll. C. Sutcharit, S. Panha (2 shells; Fig. 9D) from the type locality.

###### Type locality.

Doi Ang Khang, Fang District, Chiang Mai Province, Thailand, 19°52'09.6"N, 99°03'17.4"E, 1341 m amsl.

###### Diagnosis.

Shell ovate to ovate conical, widest at penultimate whorl; ca. 7½ whorls, with regular weak ribs. Area between ribs with very fine radial striation. Outer peristome expanded and reflected.

###### Differential diagnosis.

*Pseudopomatiasdoiangkhangensis* sp. nov. is similar to the ovate-shaped *P.harli* Páll-Gergely, 2015 (Páll-Gergely et al. 2015), but differs in having more whorls, weaker ribs, and a wider apertural lip. In addition, the shell is widest at its penultimate whorl, compared to *P.harli* that is widest at its last whorl.

###### Description.

Shell height 8.8–9.2 mm; shell width 4.4–4.6 mm. Shell ovate to ovate conical, widest at penultimate whorl, solid, semi-transparent, pale orange. Whorls ca. 7½ with sutures deep. Protoconch ca. 2 whorls (slightly eroded), first ca. 1½ whorl very finely granulated; remaining whorls and teleoconch very finely, regularly ribbed every 0.2 mm; ribs weak and 0.1 mm wide. Area between ribs with very fine radial lines, visible only under high magnification (> 20×), getting weaker in earlier whorls. Last whorl with 28–30 ribs. Apex obtuse. Spire angle ca. 50°. Aperture rounded with very slightly angled columellar-parietal transition and more sharply angled parietal-palatal transition; outer peristome expanded and reflected (0.4–0.5 mm wide and 0.3 mm thick), white to pale pinkish in colour. Umbilicus closed. Operculum unknown.

###### Etymology.

The specific epithet is named after Doi Ang Khang, the type locality of this species.

###### Distribution.

Known only from the type locality.

###### Remarks.

This species exhibits infraspecific variation in shell shape from ovate to ovate conical (Fig. 9C, D).

##### ﻿ Pseudopomatias pallgergelyi

Taxon classificationAnimaliaArchitaenioglossaPupinidae

Jirapatrasilp sp. nov.

8A725E8E-7C17-5AD6-95AF-9EF4EF6C318F

https://zoobank.org/804C66C4-EA2C-4692-9BFE-3D7E612B9616

Fig. 9E, F

###### Type material.

***Holotype***CUMZ 12167/1 (Fig. 9E), 18 Jan. 2015, coll. C. Sutcharit, P. Jirapatrasilp, W. Siriwut, R. Srisonchai, T. Seesamut. Measurement: shell height 14.5 mm, shell width 4.9 mm and 11 whorls. ***Paratypes***CUMZ 12167/2–4 (3 shells; Fig. 9F) and NHMUK 20210332 (1 shell), same data as holotype.

###### Type locality.

Pha Daeng Cave, Mueang Mae Hong Son District, Mae Hong Son Province, Thailand, 19°25'23.9"N, 97°59'03.1"E, 270 m amsl.

###### Diagnosis.

Shell elongate turriform; ca. 11 whorls, with regular strong ribs separated by wide space. Area between ribs with very fine spiral striation. Outer peristome expanded and strongly reflected.

###### Differential diagnosis.

*Pseudopomatiaspallgergelyi* sp. nov. can be distinguished from *P.caligosus* and *P.shanensis* by a more slender shell shape with more whorls that are less bulging, stronger ribs that are nearly twice as widely spaced, and a more expanded and strongly reflected outer peristome.

###### Description.

Shell height 14.0–14.6 mm; shell width 4.8–5.1 mm. Shell elongate turriform, widest at its base, solid, semi-transparent, whitish to pale pinkish. Whorls ca. 11 with sutures deep. Protoconch ca. 2 whorls (slightly eroded), first ca. 1½ whorl very finely granulated; remaining whorls and teleoconch very finely, regularly ribbed every 0.4–0.5 mm; ribs strong 0.1 mm wide, triangular in cross section. Area between ribs with very fine spiral lines, visible only under high magnification (> 20×). Last whorl with 20–26 ribs. Apex obtuse. Spire angle ca. 30°. Aperture rounded with very slightly angled columellar-parietal transition and more sharply angled parietal-palatal transition appearing as indentation; outer peristome expanded and strongly reflected (0.5–0.6 mm wide and 0.5 mm thick), white to pale pinkish in colour. Umbilicus closed. Operculum unknown.

###### Etymology.

The specific epithet is dedicated to B. Páll-Gergely, a Hungarian malacologist who extensively studies the taxonomy and systematics of Southeast Asian land snails, especially revising the taxonomy of the genus *Pseudopomatias*.

###### Distribution.

Known only from the type locality.

##### ﻿4. Genus Pupinella

Taxon classificationAnimaliaArchitaenioglossaPupinidae

Gray, 1850

58203E38-1F46-5FB8-A40B-6F87B0B80280

Pupinella Gray, 1850: 33. Kobelt 1902: 291. Egorov 2013: 9.

###### Type species.

*Cyclostomapupiniforme* Sowerby I, 1842, by original designation.

###### Diagnosis.

Shell with funnel- or gutter-like [= umbilical passage in Varga and Páll-Gergely (2017)] anterior canal forming a tube opening at both ends, appearing as a slit when observed from apertural view that is widened or slightly widened on outer margin.

###### Differential diagnosis.

**Figure 10.**
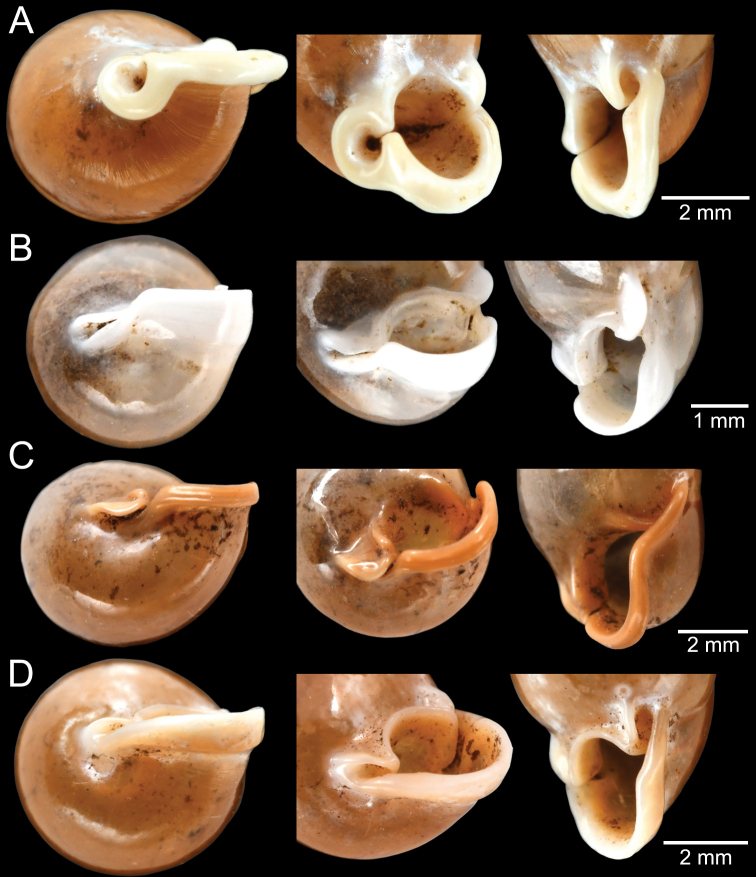
Umbilical, columellar and parietal views of **A***Pupinellamansuyi*, specimen CUMZ 12148 from Pha Chu, Nan **B***Pupinaartata* from the *Pupinaartata* species group, specimen CUMZ 12003 from Ban Ping Khong, Chiang Mai **C***Pupinagodwinausteni* sp. nov. from the *Pupinaarula* species group, holotype CUMZ 12090/1 **D***Pupinaaureola* from the *Pupinaaureola* species group, specimen CUMZ 12130 from Sra Morakot, Krabi.

*Pupinella* is most similar to *Pupina* in shell shape and the presence of both teeth and canals, but differs in having an umbilical passage or a funnel-like anterior canal forming a tube opening at both ends (Fig. 10A; Varga and Páll-Gergely 2017). The comparison of the umbilical, columellar, and parietal views between *Pupinella* and *Pupina* is illustrated in Fig. 10.

###### Remarks.

**Figure 11.**
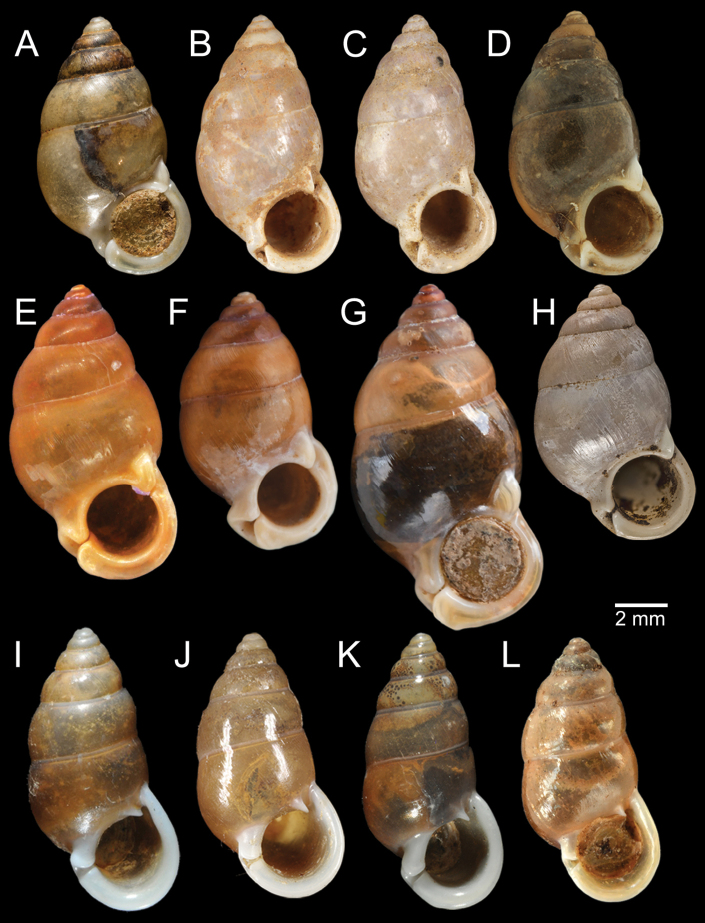
Shells of *Pupinella* species from mainland Southeast Asia. **A–G***Pupinellamansuyi*: **A** syntype of *Eupupinamansuyi*MNHN-IM-2000-30756 from Deux-Ponts **B** syntype of *Eupupinamansuyi*MNHN-IM-2000-36067 from Deux-Ponts **C** syntype of *Eupupinamansuyi*MNHN-IM-2000-36068 from Quang-Huyen **D** syntype of *Eupupinamansuyi*RBINS MT970/1 from Quang-Huyen **E** holotype of *Pupinellafrednaggsi*NHMUK 20170285 **F** specimen CUMZ 12148 from Pha Chu Mount, Nan, and **G** specimen CUMZ 12149 from Pha Tub Cave, Nan **H***Pupinellasonlaensis*, paratype ZRC.MOL.9377 **I–L***Pupinellaillustris***I, J** syntypes of *Pupinaillustris*MNHN-IM-2000-35842 from Tonkin **K** lectotype of *Pupinatonkiniana*MNHN-IM-2000-35838 from Lang-Son et That-Khé, and **L** paralectotype of *Pupinatonkiniana*SMF 109932/10 from Tonkin: That-khé. Photo: A. Lardeur, P. Maestrati, MNHN (**A–C, I–K**), F. Trus, RBINS (**D**), S.K. Tan, ZRC (**H**).

The most comprehensive compilation of members of this genus could be traced back to Kobelt (1902). This genus has two subgenera, the nominotypical subgenus and *Pupinopsis* H. Adams, 1866 (Kobelt 1902; Egorov 2013). The subgenusPupinopsis is diagnosed with a presence of a posterior canal, as in the type species *Pupinellaswinhoei* H. Adams, 1866 (see Hwang 2014: fig. 1g, h). On the other hand, the posterior canal is absent in the subgenusPupinella, as in the type species *Pupinellapupiniformis* (Sowerby, 1842) (see Varga and Páll-Gergely 2017: fig. 1a–c). The taxonomic works on *Pupinella* are sporadic (e.g., van Benthem Jutting 1963; Ueng and Chiou 2004) and there has been no taxonomic revision of this genus since then. Three species formerly in *Pupina* from Vietnam are now allocated to this genus (see below), and all four species from mainland Southeast Asia would belong to the subgenusPupinopsis. A synoptic view of all four *Pupinella* species is given in Fig. 11 to provide the comparative size.

##### ﻿ Pupinella mansuyi

Taxon classificationAnimaliaArchitaenioglossaPupinidae

(Dautzenberg & Fischer, 1908)

DC4253FC-E3BA-5D3E-B764-4A9A53879F8C

**Figure 12.**
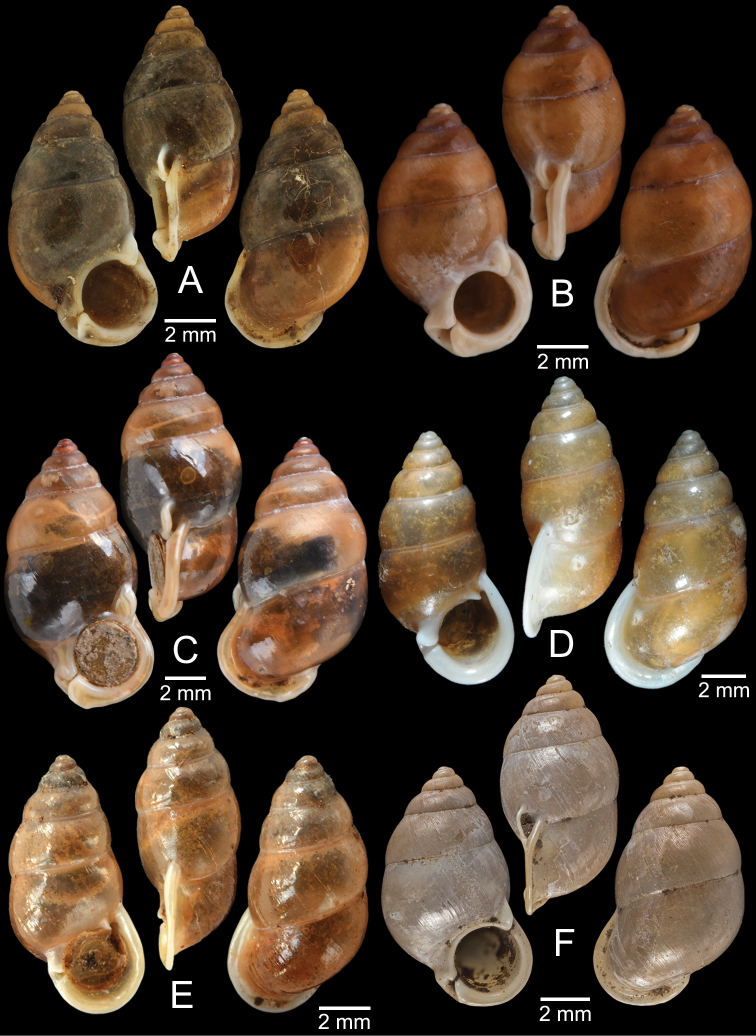
**A–C***Pupinellamansuyi*: **A** syntype of *Eupupinamansuyi*RBINS MT970/1 from Quang-Huyen **B** specimen CUMZ 12148 from Pha Chu Mount, Nan, and **C** specimen CUMZ 12149 from Pha Tub Cave, Nan. **D, E***Pupinellaillustris***D** syntype of *Pupinaillustris*MNHN-IM-2000-35842 from Tonkin and **E** paralectotype of *Pupinatonkiniana*SMF 109932/10 from Tonkin: That-khé **F***Pupinellasonlaensis*, paratype ZRC.MOL.9377. Photo: F. Trus, RBINS (**A**), P. Maestrati, MNHN (**D**), S.K. Tan, ZRC (**F**).

Figs 10A , 11A–G , 12A–C

Eupupina mansuyi Dautzenberg & Fischer, 1908: 207, 208, pl. 6, figs 12–15. Type locality: Deux-Ponts [in northeastern Vietnam]; Quang-Huyen [Quang Uyen, Cao Bang Province, Vietnam].
Pupina mansuyi —Saurin 1953: 113, environs du village méo de Pah Hia, à 100 kilomètres au Sud de Xieng-Khouang, chef-lieu de la province du Tran Ninh, Laos [probably refers to Ban Namthong, Longchaeng District, Xaisomboun Province, Laos]. Fischer 1963: 33.
Pupinella mansuyi —Do et al. 2015: 128, fig. 7c, Son La Province, Vietnam. Inkhavilay et al. 2019: 46, 47, fig. 16d.
Pupinella frednaggsi Thach & Huber in Thach, 2017: 19, 20, figs 124–130. Type locality: suburb of Luang Phrabang, Laos. Inkhavilay et al. 2019: 46, figs 16b, c, 18h, Tam Phatok Cave, Ngoy District, Luang Phrabang Province. Páll-Gergely et al. 2020: 41, Nam Wu, Ban Pak Ou, Luang Phrabang Province. Syn. nov.
Pupinella franzhuberi Thach, 2020: 21, figs 161–165. Type locality: Luang Prabang, Laos. Syn. nov.

###### Type material examined.

***Syntype*** of *Eupupinamansuyi*MNHN-IM-2000-30756 from Deux-Ponts (1 shell; Fig. 11A, Inkhavilay et al. 2019: fig. 16d). ***Syntypes*** of *Eupupinamansuyi*MNHN-IM-2000-36067 (10 shells; Fig. 11B) from Deux-Ponts. ***Syntypes*** of *Eupupinamansuyi*MNHN-IM-2000-36068 (5 shells; Fig. 11C) from Quang-Huyen. ***Syntypes*** of *Eupupinamansuyi*RBINS MT970/1 (5 shells; Figs 11D, 12A) from Quang-Huyen. ***Holotype*** of *Pupinellafrednaggsi*NHMUK 20170285 (Fig. 11E, Inkhavilay et al. 2019: fig. 16b). ***Holotype*** of *Pupinellafranzhuberi*MNHN-IM-2000-35510 figured in Thach (2020: figs 161–165).

###### Other material examined.

CUMZ 12148 (38 shells; Figs 10A, 11F, 12B) from Pha Chu, Na Noi District, Nan Province, 12 Jan. 2008. CUMZ 12149 (3 specimens in ethanol; Figs 11G, 12C) from Pha Tub Cave, Mueang Nan District, Nan Province, 11 Oct. 2009. CUMZ 12150 (15 specimens in ethanol) from Pha Tub Cave, Mueang Nan District, Nan Province, 24 Aug. 2014. CUMZ 12151 (1 shell) from Pha Tub Cave, Mueang Nan District, Nan Province, 22 Feb. 2019. CUMZ 12152 (2 shells) from Tham Phajarui Temple, Pa Daet District, Chiang Rai Province, 25 Oct. 2008. CUMZ 12153 (66 shells) from Tham Phra Bamphen Bun Temple, Phan District, Chiang Rai Province, 29 Nov. 2009.

###### Diagnosis.

Shell fusiform; last whorl ca. 60% of shell height. Apertural lip highly expanded and reflected; inner peristome thickened and cord-like; apertural lip when observed from lateral view almost straight. Parietal callus thickened and cord-like. Parietal tooth fin-shaped, highly thickened, covering posterior canal. Anterior canal funnel-like. Umbilicus closed.

###### Differential diagnosis.

*Pupinellamansuyi* can be distinguished from all other species in mainland Southeast Asia by a highly expanded and reflected apertural lip with a thickened, cord-like inner peristome. Comparing to *P.sonlaensis* and *P.thaitranbaii*, this species has a thicker and more cord-like parietal callus as well as a thicker fin-shaped parietal tooth.

###### Distribution.

Northern Vietnam (Do et al. 2015), Luang Phrabang Province, Laos (Inkhavilay et al. 2019; Páll-Gergely et al. 2020), Nan and Chiang Rai provinces, northern Thailand.

###### Remarks.

Upon examining the type specimens of *P.mansuyi*, *P.frednaggsi*, and *P.franzhuberi*, the holotypes of *P.frednaggsi* and *P.franzhuberi* agree well with all the type specimens of *P.mansuyi* in having a fusiform shell shape, a highly expanded and reflected apertural lip with a thickened cord-like peristome, parietal callus, and a highly thickened, fin-shaped, parietal tooth covering the posterior canal. Moreover, the distinctions of *P.frednaggsi* and *P.franzhuberi* from *P.mansuyi* as indicated in the original descriptions should be treated as infraspecific variation. Thus, *P.frednaggsi* and *P.franzhuberi* are regarded herein as junior subjective synonyms of *P.mansuyi*. The absence of a columellar tooth in the syntype of *Eupupinamansuyi* from Deux-Ponts (Fig. 11A) is likely due to teratological conditions. This species has a wide distribution range from northern Vietnam to northern Thailand. The distribution of this species in Thailand is provided in Fig. 7.

#### Species from other parts of mainland Southeast Asia not recorded for Thailand

##### ﻿ Pupinella illustris

Taxon classificationAnimaliaArchitaenioglossaPupinidae

(Mabille, 1887) comb. nov.

210059B2-AF71-51B8-8210-80CDE3E8D9AD

Figs 11I–L , 12D, E

Pupina illustris Mabille, 1887: 136, 137. Type locality: Tonkin. Fischer 1891: 107. Fischer and Dautzenberg 1904: 431.
Pupina tonkiniana Bavay & Dautzenberg, 1899: 54, 55, pl. 3, fig. 6, 6a (as Pupinatonkiana in the original description). Type locality: Entre Lang-Son [Lang Son Province, Vietnam] et That-Khé [That Ke, Lang Son Province, Vietnam]. Syn. nov.
Pupina (Tylotoechus) illustris —Kobelt 1902: 314, 315.
Pupina (Tylotoechus) tonkiniana —Kobelt 1902: 323, 324. Zilch 1957: 48.
Pupina tonkiniana —Fischer and Dautzenberg 1904: 432. Fischer-Piette 1950: 167. Do et al. 2015: 126, fig. 6b, Son La Province, Vietnam. Raheem et al. 2017: 5 (plate figure).

###### Type material examined.

***Syntypes*** of *Pupinaillustris*MNHN-IM-2000-35842 (9 shells; Figs 11I, J, 12D) from Tonkin. ***Lectotype*** of *Pupinatonkiniana*MNHN-IM-2000-35838 (Fig. 11K) from Lang-Son et That-Khé. Paralectotypes of *Pupinatonkiniana*SMF 109932/10 (10 shells; Figs 11L, 12E) from Tonkin: That-khé. Paralectotypes of *Pupinatonkiniana*RBINS MT976/2 (14 shells) from Lang Son et That-khé.

###### Diagnosis.

Shell elongate fusiform; last whorl ca. 55–60% of shell height. Apertural lip expanded and slightly reflected; apertural lip when observed from lateral view almost straight. Parietal callus absent. Parietal tooth pointily sharp, located next to wide posterior canal. Anterior canal funnel-like. Umbilicus closed.

###### Differential diagnosis.

*Pupinellaillustris* can be distinguished from all other species in mainland Southeast Asia by an elongate fusiform shell shape, an absence of parietal callus and a pointily sharp parietal tooth located next to a wide posterior canal.

###### Distribution.

Northern Vietnam (Do et al. 2015; Raheem et al. 2017).

###### Remarks.

This taxon is allocated to the genus *Pupinella* due to the presence of a funnel-like anterior canal, which is the diagnostic character of this genus. In the original description of *Pupinatonkiniana*, two ways of spelling were shown: the spelling ‘*tonkiana*’ in the description, and ‘*tonkiniana*’ in the plate caption. Later, Kobelt (1902) acted as the First Reviser (ICZN 1999: Art. 24.2.3) in selecting ‘*tonkiniana*’ as the correct original spelling. As the original description did not explicitly state that the description of *P.tonkiniana* was based on a single specimen (nor could this be inferred), the designation of a holotype by Fischer-Piette (1950) in fact constitutes a lectotype designation (ICZN 1999: Art. 74.6).

Upon examining the type specimens of both *P.illustris* and *P.tonkiniana*, the type series of *P.tonkiniana* agree well with all the syntypes of *P.illustris* in having an elongate fusiform shell shape, an expanded and slightly reflected apertural lip without a parietal callus, and a sharp, tooth-like, parietal tooth located next to a wide posterior canal. Thus, *P.tonkiniana* is regarded herein as a junior subjective synonym of *P.illustris*.

##### ﻿ Pupinella sonlaensis

Taxon classificationAnimaliaArchitaenioglossaPupinidae

(Do, 2017) comb. nov.

8831BC71-A344-5ADD-8A6E-49F4F37F8A27

Figs 11H , 12F

Pupina sonlaensis Do, 2017: 300, 302, figs 2a, 3a. Type locality: limestone karst in Muong Bu Commune, Muong La District, Son La Province, Vietnam.

###### Type material examined.

***Holotype***HNUE-OC 00108 figured in Do (2017: figs 2a, 3a). ***Paratypes***ZRC.MOL.9377 (3 shells; Figs 11H, 12F) from the type locality.

###### Diagnosis.

Shell ovate-fusiform; last whorl ca. 60% of shell height. Apertural lip slightly expanded and reflected, thickened cord-like peristome absent; apertural lip when observed from lateral view almost straight. Parietal callus somewhat distinct and cord-like. Parietal tooth sharp with wide base, thickened and covering posterior canal. Anterior canal funnel-like, appearing as a slit on the inside, widened on outer margin, bordered by a thickened columellar margin. Umbilicus closed.

###### Differential diagnosis.

*Pupinellasonlaensis* is most similar to *P.mansuyi* in shell size, but differs in having an ovate-fusiform shell shape with a less thickened parietal tooth, as well as a less thickened, expanded, and reflected apertural lip without a thickened cord-like inner peristome.

###### Distribution.

Muong La District, Thuan Chau District, and Van Ho District, Son La Province, Vietnam (Do 2017).

###### Remarks.

This taxon is allocated to the genus *Pupinella* due to the presence of a funnel-like anterior canal, which is the diagnostic character of this genus. The paratype figured in this study is similar to *P.mansuyi* in having a triangular parietal tooth covering the posterior canal and an expanded and reflected apertural lip with somewhat cord-like inner peristome, although with less thickening, and the shell has a less elongate shape. However, the holotype of *P.sonlaensis* figured in Do (2017: figs 2a, 3a) has an ovate-fusiform shell with a thickened, wide-based parietal tooth not covering the posterior canal, and a slightly expanded and reflected apertural lip without a thickened cord-like inner peristome. A thorough examination of the specimens would clarify whether the type series contain more than one taxon or whether the validity of this taxon should be reassessed.

##### ﻿ Pupinella thaitranbaii

Taxon classificationAnimaliaArchitaenioglossaPupinidae

(Do, 2017) comb. nov.

25F6F493-DD55-5865-9E32-C86701C4F2B9

Pupina thaitranbaii Do, 2017: 302, 303, figs 2b, 3b. Type locality: limestone forest in Pa Cop Village, Van Ho Commune, Van Ho District, Son La Province, Vietnam.

###### Type material examined.

***Holotype***HNUE-OC 00109 figured in Do (2017: figs 2b, 3b).

###### Diagnosis.

Shell ovate-fusiform; last whorl ca. two-thirds of shell height. Apertural lip expanded and slightly reflected; apertural lip curved when observed from lateral view. Parietal callus somewhat thickened and cord-like. Parietal tooth thickened, fin-shaped, covering posterior canal. Anterior canal forming a long gutter, extending into a spike-like protrusion. Umbilicus open and deep.

###### Differential diagnosis.

*Pupinellathaitranbaii* can be distinguished from all other species in mainland Southeast Asia by having an anterior canal forming a long gutter and extending into a spike-like protrusion, a curved apertural lip when observed from lateral view, and an open and deep umbilicus.

###### Distribution.

Known only from the type locality (Do 2017).

###### Remarks.

This taxon is allocated to the genus *Pupinella* due to the presence of a funnel-like anterior canal, which is the diagnostic character of this genus.

##### ﻿5. Genus Rhaphaulus

Taxon classificationAnimaliaArchitaenioglossaPupinidae

Pfeiffer, 1856

3AE051D4-5003-52C7-BF30-03C1F83F2907

Rhaphaulus Pfeiffer, 1856b: 75. Kobelt 1902: 274, 275. Egorov 2013: 12.

###### Type species.

*Anaulusbombycinus* Pfeiffer, 1855, by monotypy.

###### Diagnosis.

Shell pupoid, with large penultimate whorl dominating the shell, being almost as wide as upper whorls combined when observed from apertural view. Peristome continuous, with parietal callus well-developed. Aperture shifting to the right side of the shell. Inner tube or breathing device short (of c. 0.25 whorl). Outer tube not perforated and varies in direction, never running strictly along the suture.

###### Differential diagnosis.

*Rhaphaulus* is most similar to *Streptaulus* Benson, 1857 and *Barnaia* Thach, 2017 in shell shape and size (8–19 mm) and a thin operculum. Both *Rhaphaulus* and *Streptaulus* have two portions of a breathing tube: an inner portion starting from the peristome and running internally and posteriorly under the suture to its inner opening within the body whorl, and an outer portion extending from the parieto-palatal junction of the peristome to the outer opening, whereas *Barnaia* lacks this outer portion. However, *Rhaphaulus* differs from *Streptaulus* in having a continuous peristome with well-developed parietal callus, and an outer tube without holes on side wall, whereas *Streptaulus* has an interrupted peristome with weak parietal callus, as well as several circular holes along the tube’s wall when the outer tube is present (Páll-Gergely et al. 2014, 2017).

###### Remarks.

Pfeiffer (1855) proposed a monotypic genus *Anaulus* with ‘*A.bombycinus*’ as the type species. However, this generic name was occupied by *Anaulus* Ehrenberg, 1844 (a diatom genus in the phylum Ochrophyta), hence *Anaulus* Pfeiffer, 1855 became a junior homonym. Later, Pfeiffer (1856b), under the remark of ‘*Rhaphauluslorraini* Pfr.’, stated that the generic name *Rhaphaulus* was to replace the junior homonym *Anaulus* Pfeiffer, 1855. The distribution of *Rhaphaulus* species in Thailand is provided in Figs 4, 7.

##### ﻿ Rhaphaulus lorraini

Taxon classificationAnimaliaArchitaenioglossaPupinidae

Pfeiffer, 1856

D5AAE266-8C79-5040-A997-B57F4BB0D001

Fig. 13A, B

Rhaphaulus lorraini Pfeiffer, 1856a: 36. Type locality: Pulo Penang [Penang Island, Penang State, Malaysia]. Pfeiffer 1856b: 75, pl. 20, figs 21, 22. von Martens 1867: 155. de Morgan 1885: 413. Smith 1898: 18, figs 3, 4. Kobelt 1902: 276. Laidlaw 1928: 33, Penang. Maassen 2001: 42, West Malaysia. Páll-Gergely et al. 2014: 572, fig. 9. BEDO 2017: 96.
Rhaphaulus lorainii [sic]—Sowerby I 1866: Pupinidae, pl. 2 (pl. 264), Rhaphaulus, fig. 5. Reeve 1878: Pupinidae, pl. 10, sp. 96.
Rhaphaulus lorrainii [sic]—Habe 1965: 115, 116, pl. 2, fig. 12, as a synonym of Rhaphauluschrysalis, Khao Chong, Trang Province, peninsular Thailand.
Rhaphaulus chrysalis ?—Maassen 2001: 42, West Malaysia.

###### Type material examined.

**Figure 13.**
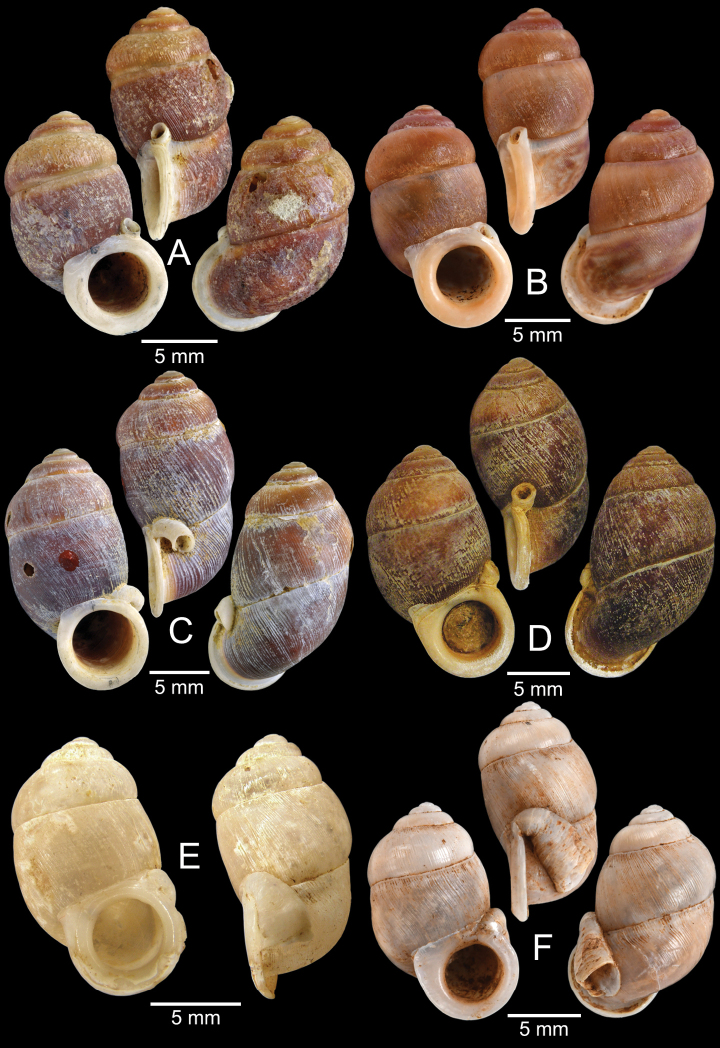
**A, B***Rhaphauluslorraini*: **A** syntype NHMUK 20130454 from Pulo Penang and **B** specimen CUMZ 12162 from Kiriwong (Tham Kope) Temple, Phang Nga **C***Rhaphaulusperakensis*, syntype NHMUK 1897.3.15.41 from Larut, Perak **D***Rhaphaulusascendens*, syntype UMZC I.100025 from Patalung, Malay Peninsula **E, F***Rhaphaulustonkinensis***E** holotype HNHM 98757 and **F** specimen CUMZ 12163/1 from Luang Cave, Chiang Rai. Photo: B. Páll-Gergely (**E**)

***Syntypes***NHMUK 20130454 (3 shells; Fig. 13A) from Pulo Penang.

###### Other material examined.

CUMZ 12162 (1 shell; Fig. 13B) from Kiriwong (Tham Kope) Temple, Thap Put District, Phang Nga Province, 16 Jan. 2009.

###### Diagnosis.

Shell ovate; body whorls bulging. Tube cylindrical, pointing upward and forward.

###### Differential diagnosis.

*Rhaphauluslorraini* can be distinguished from all other species from mainland Southeast Asia by a cylindrical tube pointing upward and forward.

###### Distribution.

Malaysia and southern Thailand (Laidlaw 1928; Páll-Gergely et al. 2014).

###### Remarks.

It is possible that *R.chrysalis* sensu Habe (1965) from Khao Chong, Trang Province, southern Thailand is *R.lorraini*. This species is distributed in the Malay Peninsula and is disjunct from *R.chrysalis*, which is distributed in northeastern India and Myanmar. See also remarks in *R.ascendens*.

##### ﻿ Rhaphaulus perakensis

Taxon classificationAnimaliaArchitaenioglossaPupinidae

Smith, 1898

CAF1EE5E-3DF7-5B39-AD03-55F922D83BAA

Fig. 13C

Rhaphaulus perakensis Smith, 1898: 17, figs 1, 2. Type locality: Maxwell’s Hill, Larut [Bukit Larut], Perak. Kobelt 1902: 276, 277. Laidlaw 1928: 32, 33. van Benthem Jutting 1949: 57, Kuala Kenering; Maxwell’s Hill, Perak; Dusun Tua, Selangor [Malaysia]. Habe 1965: 115, 116, as a synonym of Rhaphauluschrysalis. Hemmen and Hemmen 2001: 40, Thailand. Páll-Gergely et al. 2014: 572, fig. 12, western Malaysia. BEDO 2017: 97.
Rhaphaulus perakensis var. jalorensis Sykes, 1903: 197, pl. 20, figs 9, 10. Type locality: Bukit Bisar, on the borders of Jalor [Khao Yai National Reserved Forest, Namtok Sai Khao National Park, Mueang Yala District, Yala Province, Thailand].
Rhaphaulus perakensis var. ialorensis [sic]—Laidlaw 1928: 33.
Rhaphaulus perakensis jalorensis —Maassen 2001: 42.
Rhaphaulus perakensis perakensis —Maassen 2001: 42.
Rhaphaulus jalorensis —Páll-Gergely et al. 2014: 572, western Malaysia. BEDO 2017: 96. Sutcharit et al. 2018: 157, fig. 5–13l.

###### Type material examined.

***Syntypes*** of *Rhaphaulusperakensis*NHMUK 1897.3.15.41–2 (2 shells; Fig. 13C) from Larut, Perak.

###### Diagnosis.

Shell elongate ovate; body whorls slightly bulging. Tube cylindrical, pointing diagonally downward and backward.

###### Differential diagnosis.

*Rhaphaulusperakensis* can be distinguished from all other species from mainland Southeast Asia by a cylindrical tube pointing diagonally downward and backward.

###### Distribution.

Northern Peninsular Malaysia and southern Thailand (Maassen 2001; Páll-Gergely et al. 2014).

###### Remarks.

No material of this species was found during this survey. Maassen (2001) treated *R.perakensisjalorensis* as a junior subjective synonym of *R.p.perakensis* without apparent reason, whereas Páll-Gergely et al. (2014) listed this subspecies as a valid species following the opinion of Sykes (1903).

##### ﻿ Rhaphaulus ascendens

Taxon classificationAnimaliaArchitaenioglossaPupinidae

Sykes, 1903

182BAF49-BB25-5768-9571-EDB181FD596B

Fig. 13D

Rhaphaulus ascendens Sykes, 1903: 196, 197, pl. 20, figs 11, 12. Type locality: Patalung [Phatthalung Province, Thailand]. Laidlaw 1928: 33. Hemmen and Hemmen 2001: 40. Páll-Gergely et al. 2014: 572. Thach 2018: 21, figs 126–129, Phang Nga District, South Thailand. BEDO 2017: 95. Sutcharit et al. 2018: 157, figs 5–11d, 5–13k.

###### Type material examined.

***Syntype***UMZC I.100025 (1 shell; Fig. 13D) from Patalung, Malay Peninsula.

###### Diagnosis.

Shell ovate; body whorls not bulging. Tube cylindrical and pointing straight upward.

###### Differential diagnosis.

*Rhaphaulusascendens* can be distinguished from all other species from mainland Southeast Asia by having body whorls that are not bulging and a cylindrical tube pointing straight upward.

###### Distribution.

Southern Thailand (Páll-Gergely et al. 2014; Thach 2018).

###### Remarks.

No material of this species was found during this survey. Laidlaw (1928) treated *R.ascendens* as a junior subjective synonym of *R.lorraini*. However, by comparing the type specimens of both species, the body whorls of *R.ascendens* are not bulging, whereas the distribution ranges tend to overlap. Thus, the validity of *R.ascendens* needs further confirmation.

##### ﻿ Rhaphaulus tonkinensis

Taxon classificationAnimaliaArchitaenioglossaPupinidae

Páll-Gergely, Hunyadi & Maassen, 2014

0475C288-DF1C-5969-9968-97D2534C4B40

Figs 13E, F , 14A

Rhaphaulus tonkinensis Páll-Gergely et al. 2014: 567, 569, fig. 1. Type locality: rocky wall, left side of the road nr. 6, 156 km towards Moc Chau, Ha Noi, Son La Province, Vietnam. Do et al. 2015: 128, fig. 7d, Son La Province, Vietnam. Páll-Gergely et al. 2017: fig. 1a–e. Raheem et al. 2017: 6 (plate figure).

###### Type material examined.

***Holotype***HNHM 98757 from Ha Noi, Son La Province, Vietnam (Fig. 13E).

###### Other material examined.

**Figure 14.**
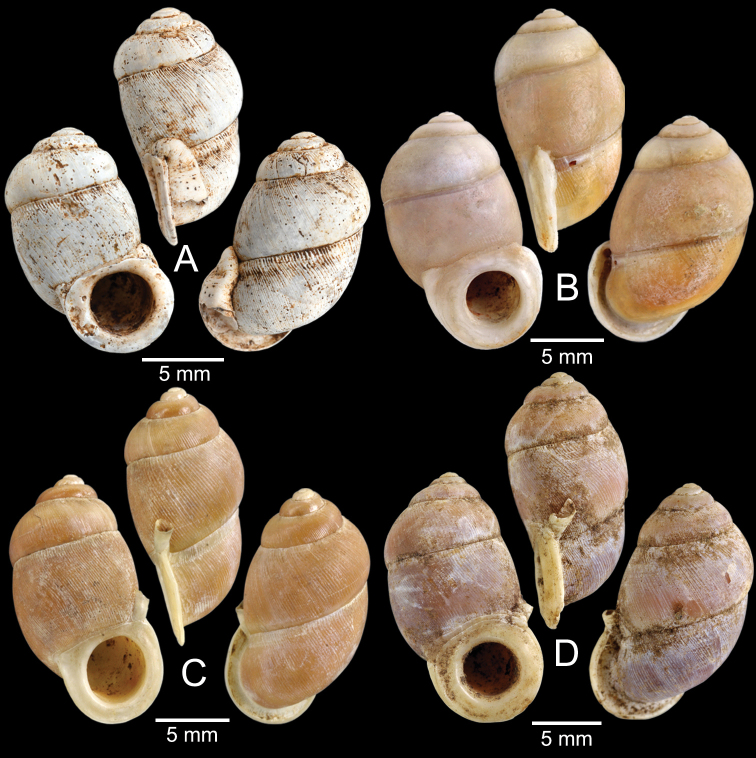
**A***Rhaphaulustonkinensis*, specimen CUMZ 12163/2 from Luang Cave, Chiang Rai. **B–D***Rhaphauluschrysalis***B** possible syntype NHMUK 2013.04.16 from Siam **C** specimen NHMUK 1871.9.23.52 from Burma, and **D** specimen NHMUK 1903.7.1.3073 from Molmein.

CUMZ 12163 (4 shells; Figs 13F, 14A) from Luang Cave, Mae Sai District, Chiang Rai Province, 23 Oct. 2015. CUMZ 12164 (2 shells) from Pha Mee Cave, Mae Sai District, Chiang Rai Province, 23 Oct. 2015.

###### Diagnosis.

Shell elongated ovate; body whorls slightly bulging. Tube thick and flat, turning first straight upward then abruptly downward, highly widening and extending to nearly the entire last whorl height.

###### Differential diagnosis.

*Rhaphaulustonkinensis* can be distinguished from all other species from mainland Southeast Asia by a distinctive tube that is thick and flat, turning first straight upward then abruptly downward, greatly widening and extending to nearly the entire last whorl height.

###### Distribution.

Northern Vietnam (Do et al. 2015) and Chiang Rai Province, northern Thailand.

###### Remarks.

The tube of one specimen from Tham Luang, Mae Sai District, Chiang Rai Province when turning downward does not adhere to the apertural margin (Fig. 13F). However, the tube of another specimen from the same locality adheres to the apertural margin (Fig. 14A), identical to the holotype (Páll-Gergely et al. 2014). Thus, the extent of tube adherence to the apertural margin is treated as an infraspecific variation.

#### Species with uncertain record from Thailand

##### ﻿ Rhaphaulus chrysalis

Taxon classificationAnimaliaArchitaenioglossaPupinidae

(Pfeiffer, 1853)

9742492B-3726-5D68-AFFB-450AED24E5A4

Fig. 14B–D

Cyclostoma chrysalis Pfeiffer, 1853: 239, pl. 31, figs 23, 24. Type locality: Arva [Mandalay Region, Myanmar]. Pfeiffer 1854: 158.
Rhaphaulus chrysalis —Theobald 1858[1857]: 247, Maulmein [Mawlamyine, Mawlamyine Township, Mawlamyine District, Mon State, Myanmar]. Sowerby I 1866: Pupinidae, pl. 2 (pl. 264), Rhaphaulus, figs 6, 7, Siam. Hanley and Theobald 1875: 53, pl. 133, fig. 7. Nevill 1878: 301. Reeve 1878: Pupinidae, pl. 10, sp. 95. Godwin-Austen 1886: 200, 201, pl. 47, fig. 1, 1a. Tapparone-Canefri 1889: 310. Smith 1898: 19. Kobelt 1902: 275, 276. Gude 1921: 165, 166, fig. 24. Páll-Gergely et al. 2014: 572, fig. 11, north-eastern India and Myanmar. BEDO 2017: 95. Sutcharit et al. 2018: 157.
Raphaulus [sic] chrysalis—Stoliczka 1871: 151, farm caves, near Moulmein, Myanmar.

###### Type material examined.

***Possible syntype***NHMUK 2013.04.16 (1 shell; Fig. 14B) from Siam.

###### Other material examined.

NHMUK 1871.9.23.52 (1 shell; Fig. 14C) from Burma. NHMUK 1903.7.1.3073 (2 shells; Fig. 14D) from Molmein.

###### Diagnosis.

Shell ovate; body whorls slightly bulging. Tube cylindrical, pointing upward and backward.

###### Differential diagnosis.

*Rhaphauluschrysalis* is most similar to *R.lorraini* in shell shape, but differs in having a cylindrical tube pointing upward and backward, instead of forward as in *R.lorraini*.

###### Distribution.

Northeastern India, Myanmar, and an uncertain record from Thailand (Páll-Gergely et al. 2014).

###### Remarks.

No material of this species was found during this survey, and the record in Thailand needs further confirmation. The type locality on the label of the possible type specimen is “Siam”, which is different from that reported in the original description as “Arva”. A lack of a tube in a possible syntype NHMUK 2013.04.16 (Fig. 14B) is possibly due to damage.

##### ﻿6. Genus Tortulosa

Taxon classificationAnimaliaArchitaenioglossaPupinidae

Gray, 1847

67FF90F9-F144-5C2C-946E-363E9E0BDE68

Tortulosa Gray, 1847: 177. Kobelt 1902: 281. Egorov 2013: 14.

###### Type species.

*Turbotortuosus* Férussac, 1821, by original designation.

###### Diagnosis.

Shell elongated ovate. Periumbilical keel present. Aperture almost round; basal edge of peristome with a canal or indentation extending below into periumbilical keel. Operculum moderately thick to thick, corneous, circular, flat or cylindrical, closely coiled, multi-layer.

###### Differential diagnosis.

*Tortulosa* can be distinguished from all other genera in this subfamily, especially *Coptocheilus* which has a similar shell size and matt surface, by a canal or indentation at a basal edge of peristome extending below into a periumbilical keel, and a thick, multi-layer operculum (Kobelt 1902; Raheem et al. 2014).

###### Remarks.

This genus comprises two subgenera: the nominotypical subgenus and EucataulusKobelt, 1902. ThesubgenusTortulosa possesses a detached last whorl and contains only one species, *Tortulosatortuosa*. At present, the subgenusEucataulus contains 29 species, all of which are distributed in Western Ghats, India, and Sri Lanka (Kobelt 1902; Raheem et al. 2014, 2018).

##### ﻿ Tortulosa tortuosa

Taxon classificationAnimaliaArchitaenioglossaPupinidae

(Férussac, 1821)

0C7D2D21-74BE-5698-B915-39251E676276

Figs 8D , 15 , 16

Turbo tortuosus —Chemnitz 1795: 158, 159, pl. 195, figs 1882, 1883. Type locality: Nicobarischen Eylanden [Nicobar Islands]. Unavailable name.
Helix (Cochlodina) tortuosa Férussac, 1821: 61.
Pupa tortuosa—Gray 1825: 413.
Cyclostoma tortuosum —Sowerby I 1843: 152, pl. 28, figs 185, 186. Pfeiffer 1848: pl. 24, figs 19, 20. Pfeiffer 1849: 165, 166.
Tortulosa tortuosa —Adams and Adams 1856: 285, pl. 86, fig. 2, 2a, b. Gude 1921: 190, ?Nicobars; India: Trevandrum. van Benthem Jutting 1960: 11, 12, limestone hill Kaki Bukit, near kampong Wang Tangga, Perlis [Malaysia]. Berry 1963: pl. 6, fig. 31. Hemmen and Hemmen 2001: 40, fig. 7, Wat Thum Sua, Nation Valley, near Krabi. Maassen 2001: 44. Sutcharit and Panha 2008: 50, 51, with figs, Khao Nan National Park, Nakhon Si Thammarat, Thailand. Egorov 2013: 14, 15, fig. 23. Raheem et al. 2014: 53, figs 9e, 30b, c. BEDO 2017: 98. Sutcharit et al. 2018: 159, figs 5–11f, 5–13n. Thach 2018: 97 (figure caption), figs 139, 140. Meksuwan et al. 2020: 249, fig. 2, Tonsai Waterfall, Thalang District, Phuket Province. Páll-Gergely et al. 2020: 41.
Cataulus (Tortulosa) tortuosus —Sowerby I 1866: Pupinidae, pl. 2 (pl. 264), Cataulus, fig. 1.
Cataulus tortuosus —Reeve 1878: Pupinidae, pl. 6, sp. 49. Nevill 1881: 149.
Tortulosa (Tortulosa) tortuosa —Kobelt 1902: 288, fig. 64.
Perlisia tweediei Tomlin, 1948: 225, 226, pl. 11, fig. 6. Type locality: Kaki Bukit, Perlis [Malaysia]. Páll-Gergely et al. 2020: 41, fig. 3.
Tortulosa tweediei —BEDO 2017: 98.
Tortulosa huberi Thach, 2018: 21, 22, figs 133–138. Type locality: Krabi, South Thailand. Páll-Gergely et al. 2020: 41, fig. 5.
Tortulosa schileykoi Thach & Huber in Thach, 2018: 22, figs 142–146. Type locality: Phang Nga, South Thailand. Páll-Gergely et al. 2020: 41, fig. 4.

###### Type material examined.

**Figure 15.**
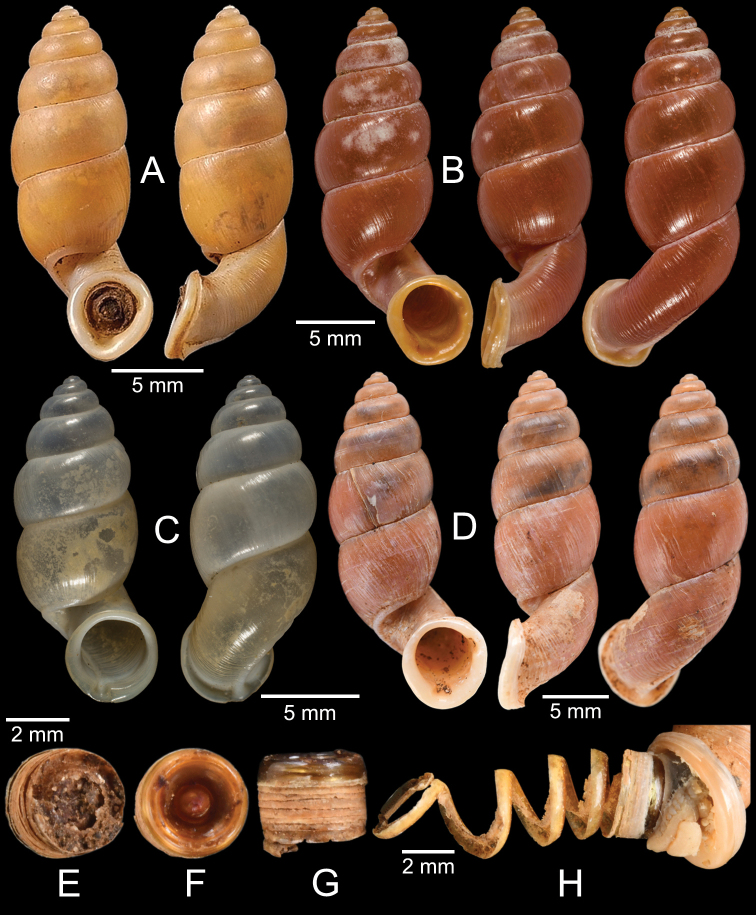
*Tortulosatortuosa***A** lectotype of *Perlisiatweediei*NHMUK 1948.10.2.6 from Kaki Bukit, Perlis **B** holotype of *Tortulosahuberi*MNHN-IM-2000-34054 from Krabi **C** holotype of *Tortulosaschileykoi*MNHN-IM-2000-34055 from Phang Nga **D** specimen CUMZ 12166 from Tham Suea Temple, Krabi, and **E–H** operculum of specimen CUMZ 12160 from Tham Kanlayanamit Temple, Nakhon Si Thammarat, showing **E** outer operculum **F** inner operculum **G** side view (inner surface up), and **H** spring-like inner operculum when extended by force. Photo: M. Caballer, MNHM (**B, C**).

***Lectotype*** of *Perlisiatweediei*NHMUK 1948.10.2.6 (Fig. 15A) from Kaki Bukit, Perlis. ***Holotype*** of *Tortulosahuberi*MNHN-IM-2000-34054 (Fig. 15B) from Krabi Province, Thailand. ***Holotype*** of *Tortulosaschileykoi*MNHN-IM-2000-34055 (Fig. 15C) from Phang Nga Province, Thailand.

###### Material examined.

**Figure 16.**
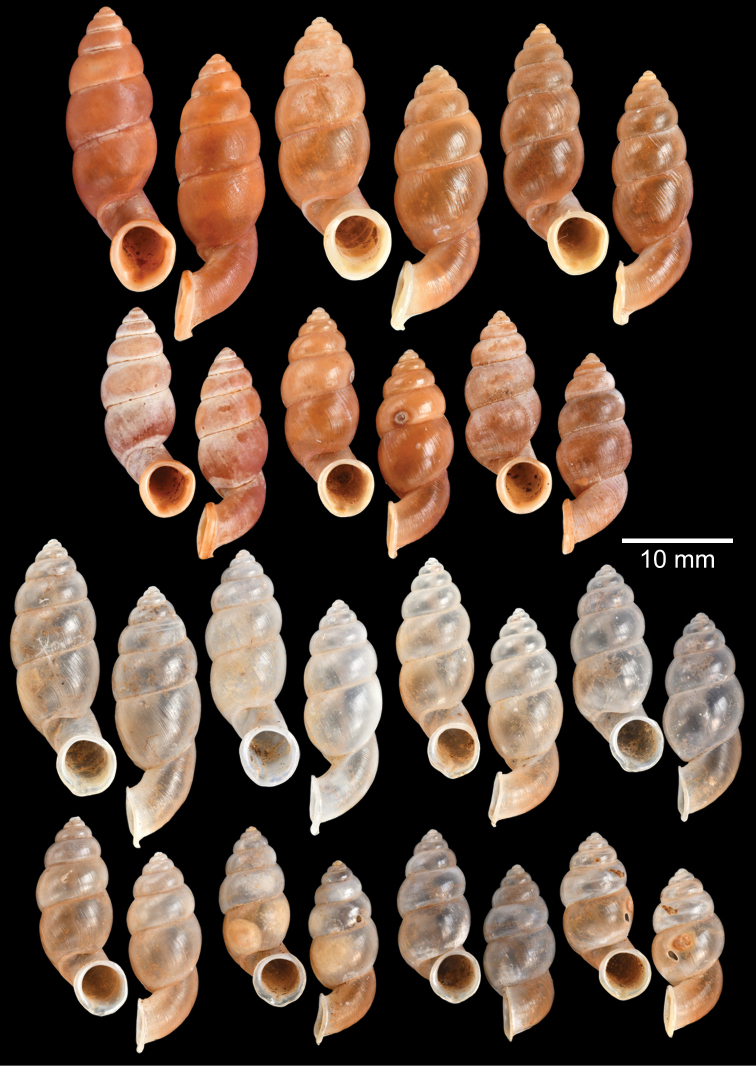
Infraspecific variation of shell shape and colour found in the same collecting locality of *Tortulosatortuosa*, CUMZ 12166 from Tham Suea Temple, Krabi.

NHMUK 20100643/1‒2 (2 shells) from the Nicobar Islands figured in Raheem et al. (2014: figs 30b, c). CUMZ 12154 (1 shell) from Nai-Chong Silvicultural Research Station, Mueang Krabi District, Krabi Province, 16 Jan. 2009. CUMZ 12166 (> 500 shells; Figs 15D, 16) from Tham Suea Temple, Mueang Krabi District, Krabi Province, 10 May 2010. CUMZ 12155 (12 specimens in ethanol; Fig. 8D) from Tham Suea Temple, Mueang Krabi District, Krabi Province, 9 July 2017. CUMZ 12156 (2 specimens in ethanol) from Phung Chang Cave, Mueang Phang Nga District, Phang Nga Province, 8 Aug. 2016. CUMZ 12157 (1 shell) from Phung Chang Cave, Mueang Phang Nga District, Phang Nga Province, 31 July 2018. CUMZ 12188 (2 shells) from Nam Phut Cave, Mueang Phang Nga District, Phang Nga Province, 7 Oct. 2010. CUMZ 12158 (1 specimen in ethanol) from Ban Yai, Phanom District, Surat Thani Province, 7 Aug. 2016. CUMZ 12159 (18 specimens in ethanol) from Khiri Rat Phatthana Temple, Wiang Sa District, Surat Thani Province, 4 July 2017. CUMZ 12189 (3 shells) from Natural Trail, Ratchaprapha Dam, Ban Ta Khun District, Surat Thani Province, 8 Dec. 2008. CUMZ 12160 (3 specimens in ethanol; Fig. 15E–H) from Tham Kanlayanamit Temple, Tham Phannara District, Nakhon Si Thammarat Province, 4 July 2017. CUMZ 12161 (3 specimens in ethanol) from Ton Din Cave, Khuan Don District, Satun Province, 7 July 2017.

###### Diagnosis.

Shell rounded, spindle-shaped, translucent whitish to brown. Whorls 7, convex; third to penultimate whorls broader; last whorl narrower, detached, brought forward, with a filiform basal keel broader at the mouth. Aperture almost circular, always with basal indentation; palatal indentation obvious in specimens with thicker shell. Operculum thick cylindrical, corneous, multi-layer, spring-like when extended by force; inner operculum (attached to dorsal side of posterior body) translucent yellow, convex with crater within and conical protrusion in the middle; outer operculum (free surface) dark brown and usually eroded.

###### Differential diagnosis.

*Tortulosatortuosa* can be distinguished from other species in this genus by a narrower last whorl that is detached from the penultimate whorl and brought forward, a shallower basal indentation, and the presence of a palatal indentation (Raheem et al. 2014).

###### Distribution.

Northern Peninsular Malaysia and southern Thailand. The type locality of this species is still controversial while the occurrences in India and Nicobar Islands need further confirmation (Sutcharit and Panha 2008; Raheem et al. 2014, 2018; Páll-Gergely et al. 2020).

###### Remarks.

The name *Turbotortuous* Chemnitz, 1795 was published prior to Férussac’s name, but it is unavailable (Raheem et al. 2018). See Raheem et al. (2014, 2018) and Páll-Gergely et al. (2020) for the notes on taxonomy and type specimen of this species. Currently, this is the only extant species in the subgenusTortulosa. One extinct species, *T.naggsi* Raheem & Schneider, 2017 in Raheem et al. (2018) was discovered from Son La Province, Northern Vietnam. This species exhibits a terminal part of the body whorl that is fully attached to the penultimate whorl, and thus corresponds more to the subgenusEucataulus from South Asia (Raheem et al. 2018).

Maassen (2001) treated *P.tweediei*, and Páll-Gergely et al. (2020) treated both *T.huberi* and *T.schileykoi* as junior subjective synonyms of *T.tortuosa*. We agree on those synonymisations because the specimens we collect from the same locality exhibit a high infraspecific variation in the length of the detached part of the body whorl relative to the shell height, also in shell shape from ovate to elongate, and shell colour from translucent whitish to brown (Fig. 16). All the specimens in Fig. 16 were found together with hundreds of other specimens inside the same decaying log at Tham Suea Temple, Krabi Province, southern Thailand. The distribution of this species in Thailand is provided in Fig. 4.

##### ﻿Subfamily Pupininae

Taxon classificationAnimaliaArchitaenioglossaPupinidae

Pfeiffer, 1853

04E9BF08-D329-5F5D-81E1-504AD67A6E76

###### Remarks.

Only one genus, *Pupina*, with a total of 14 species and one subspecies belonging to three species groups, is known to occur in Thailand, and two additional species have an uncertain record.

##### ﻿7. Genus Pupina

Taxon classificationAnimaliaArchitaenioglossaPupinidae

Vignard, 1829

EA8D61C6-996D-5718-AF27-7530E2CD113A

Pupina Vignard, 1829: 439, 440. Kobelt 1902: 302. Egorov 2013: 4, 5.

###### Type species.

*Pupinakeraudrenii* Vignard, 1829, by monotypy.

###### Diagnosis.

Shell elongate ovate, smooth, with a shining enamel-like coating. Peristome with two canals; posterior canal at the suture; anterior canal oblique at the middle of columellar margin. Parietal callus normally thickened, and bordered by two teeth; parietal tooth located near or covering posterior canal; lower columellar tooth located near or covering anterior canal (Figs 3, 10B–D).

###### Differential diagnosis.

*Pupina*, especially the *Pupinaartata* species group (see below), is most similar to *Signepupina* Iredale, 1937 and *Cordillerapina* Stanisic, 2010 in having fin-shaped teeth. However, *Signepupina* tends to have a more elongated or turriform shell shape and *Cordillerapina* has a non-glossy surface with axial ribs (Stanisic et al. 2010).

###### Remarks.

*Pupina* is the oldest taxon as well as the type genus of the family Pupinidae, and the only genus from the subfamily Pupininae occurring in mainland Southeast Asia. The three original subgenera, namely *Pupina* s. s., *Tylotoechus* Kobelt & Möllendorff, 1897, and *Siphonostyla* Kobelt, 1897 (Kobelt and von Möllendorff 1897) were adopted by later authors (Gude 1921; Egorov 2013). The subgenusSiphonostyla is diagnosed with a specialised anterior canal, which is lengthened into an ascending tube (Kobelt 1902; Egorov 2013), as in the type species *Pupinalongituba* Kobelt, 1897 (see Egorov 2013: fig. 6).

**Figure 17.**
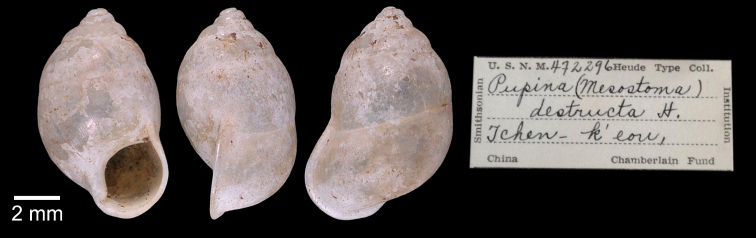
Specimen of *Pupinadestructa*, the type species of *Tylotoechus*, USNM 472296. Photo: USNM.

Various diagnoses between *Pupina* s. s. and *Tylotoechus* had been proposed by different authors (Table 2). *Tylotoechus* was originally established by Kobelt and von Möllendorff (1897) apparently to replace *Mesostoma* Heude, 1886 [non Dugès, 1830]. The type species had been subsequently designated as *Pupinadestructa* Heude, 1885 by Gude (1921), which agreed well with the original proposal by Heude (1886), in that *P.destructa* being monotypic in *Mesostoma*. Later, Clench (1949) elevated *Tylotoechus* to the generic level, and stated that many *Tylotoechus* species recognised by Kobelt (1902) should belong to *Pupina* s. s. Upon examining the type specimen figure of *P.destructa* in Heude (1885: pl. 24, fig. 15) and the specimen in the Heude Collection deposited in the National Museum of Natural History, Smithsonian Institution (USNM 472296, from the type locality, Tchen-k’eou, China; Fig. 17), we found that the parietal tooth is weak and does not extend up onto the body whorl, in contrast to the diagnostic stated in Kobelt (1902) and Clench (1949) (Table 2). It is not certain whether Heude (1885), Kobelt (1902) and Clench (1949) recognised the diagnostic characters of *Tylotoechus* in the same fashion or not.

**Table 2.** Diagnoses of the subgenera *Pupina* s. s. and *Tylotoechus* from different authors.

Author and citation	*Pupina* Vignard, 1829 Type species: *Pupinakeraudrenii* Vignard, 1829	*Tylotoechus* Kobelt & Möllendorff, 1897 Type species: *P.destructa* Heude, 1885
P.M. Heude (Heude 1885: pl. 24, fig. 15; Heude 1890: 130)	–	… interrupted peristome; columella cloven, right margin intact, parietal callus with tooth and slit.
		… The aperture is rather that of *Pupina* than *Registoma*. The columellar fissure is that of the latter, while the fissure on the right edge is missing. The parietal callus does not reach the edge, remains inwards and is rather weak, while simulating the opening of the *Pupina*, Seems to belong to the same group as *Pupinajaponica* Martens.
		(as of *Mesostoma* Heude, 1886, non *Mesostoma* Ehrenberg, 1835 [rhabdocoel flatworm])
W. Kobelt (Kobelt 1902: 302, 306, figs 70, 71)	Canal simple, formed by a tongue-like projecting callus on the apertural wall.	Upper canal formed by a tongue detached from the callus and the edge of the mouth.
W.J. Clench (Clench 1949: 31, 44, figs 17b, c, 18c, d)	Possessing a well-developed parietal tooth within margin of aperture; possessing a columellar notch cut parallel with face of aperture.	Possessing a well-developed parietal tooth extending outward and up onto body whorls; possessing a columellar notch.
		The single character upon which the genus is based is only the extension of the parietal tooth outward and upward as a tongue-like process on the body whorl in *Tylotoechus*, the parietal tooth remaining within the margin of the aperture in *Pupina*, s. s. Extremes in both cases are easily placed, but many species are exceedingly close to either of the two genera.
R. Egorov (Egorov 2013: 5–7, figs 3, 7)	Parietal canal simple, formed by tongue-shaped projecting callus, sometimes reduced. Parietal tooth differently developed.	Parietal canal formed by apertural margin and tongue-shaped projected in front process separated from callus.

Clench (1949) also established three new *Pupina*-related genera based on differences of columellar tooth from the Pacific Islands, namely *Pupinoa*, *Pupinesia*, and *Kanapa*. The current elevation of *Tylotoechus* and *Siphonostyla* to generic level, and the treatment of *Pupinoa*, *Pupinesia*, and *Kanapa* at subgeneric level (Bank 2017; MolluscaBase 2022) needs a further comprehensive revision, especially the examination of all type specimens of nominal taxa within each subgenus and the results from molecular phylogenetic analyses. As the validity of each subgenus within *Pupina* is still uncertain, this work adopts the genus *Pupina* in a wide sense, and does not apply the subgeneric classification or the elevation of those subgenera to the generic level.

**Figure 18.**
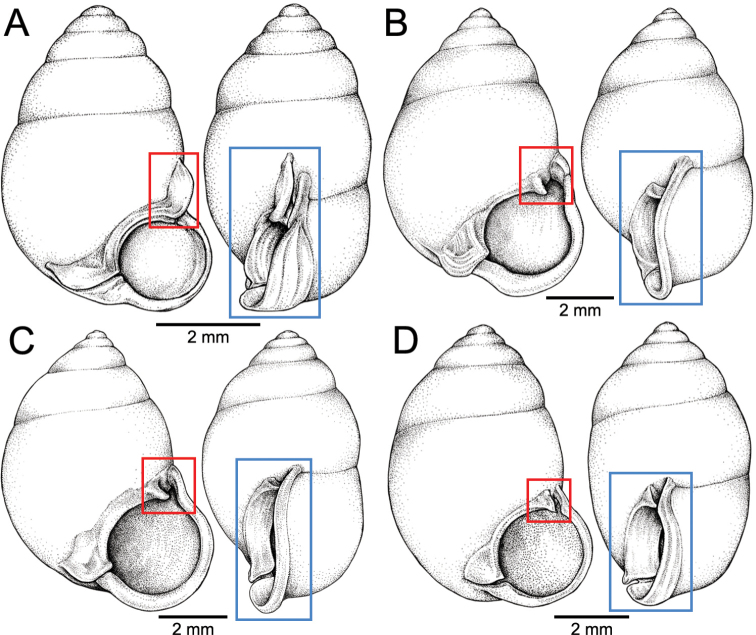
External shell morphology of three mainland Southeast Asian *Pupina* species groups **A***Pupinaartata* from the *Pupinaartata* species group, CUMZ 12003 from Ban Ping Khong, Chiang Mai **B***Pupinapeguensis* from the *Pupinaarula* species group, CUMZ 12094 from Khao Tham Phra Temple, Chiang Rai **C***Pupinasiamensis* from the *Pupinaarula* species group, CUMZ 12052 from Sri Thong Cave, Sra Keo, and **D***Pupinatchehelensis* from the *Pupinaaureola* species group, CUMZ 12136 from limestone mountain, Phang Nga. Red frames focus on the parietal tooth and posterior canal; blue frames focus on the curvature of the apertural lip when observed from lateral view.

**Figure 19.**
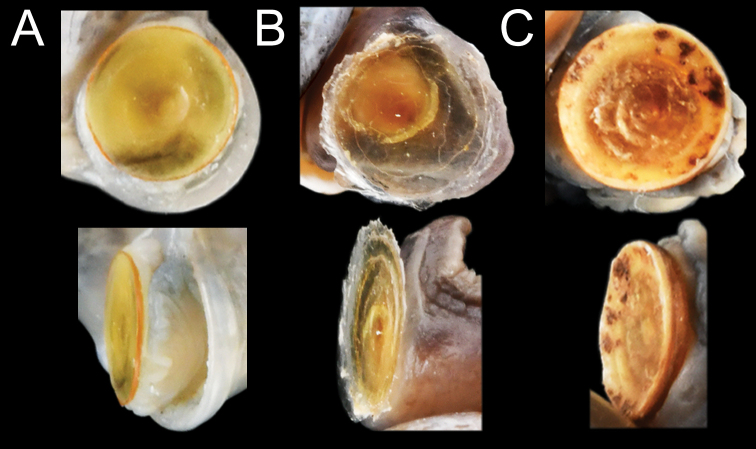
Opercula of three mainland Southeast Asian *Pupina* species groups **A***Pupinabensoni* sp. nov. from the *Pupinaartata* species group, specimen CUMZ 12047 **B***Pupinasiamensis* from the *Pupinaarula* species group, specimen CUMZ 12067, and **C***Pupinaaureola* from the *Pupinaaureola* species group, specimen CUMZ 12116. All not to scale.

Based on the distinction of shell teeth, canals (Figs 10, 18), and operculum (Fig. 19), the mainland Southeast Asian *Pupina* could be classified into three species groups, namely *P.artata* group, *P.arula* group, and *P.aureola* group. These species groups, however, might not reflect DNA-based reciprocal monophyly.

#### Group I. *Pupinaartata* species group

Figs 10B, 18A, 19A

This species group is characterised by a triangular or fin-shaped parietal tooth covering a posterior canal. A columellar tooth is less thickened, never ear-shaped and mostly fin-shaped, located next to or covering an anterior canal. When observed from apertural view, the anterior canal mostly appears slit-like and the posterior canal is not visible. An apertural lip is straight or slightly curved when observed from lateral view. An operculum is round, thin, multispiral, yellowish, transparent corneous, and with a smooth edge.

The *Pupinaartata* species group highly resembles the Australian genus *Signepupina* (type species: *Pupinellamacgillivrayi* Cox, 1864 [= *Signepupinameridionalis* (Pfeiffer, 1864)]). Both groups possess a triangular or fin-shaped parietal tooth covering the posterior canal, and the columellar tooth is mostly fin-shaped, located next to the anterior canal, making the anterior canal slit-like. However, *Signepupina* tends to have a more elongated or turriform shell shape. As the relationship between *Pupina* and *Signepupina* is still uncertain, we do not allocate the *Pupinaartata* species group from mainland Southeast Asia to *Signepupina*.

**Figure 20.**
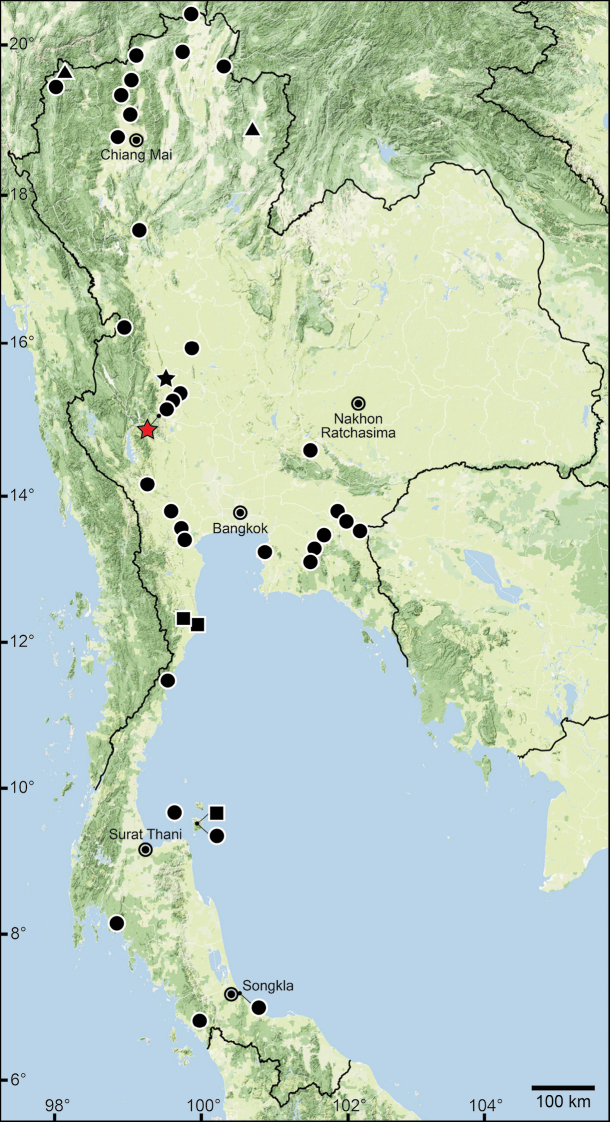
Distribution map of the *Pupinaartata* species group: *Pupinaartata* (circle), *Pupinalimitanea* (triangle), *Pupinapallens* (square), and *Pupinabensoni* sp. nov. (star) with a red star indicating the type locality.

**Figure 21.**
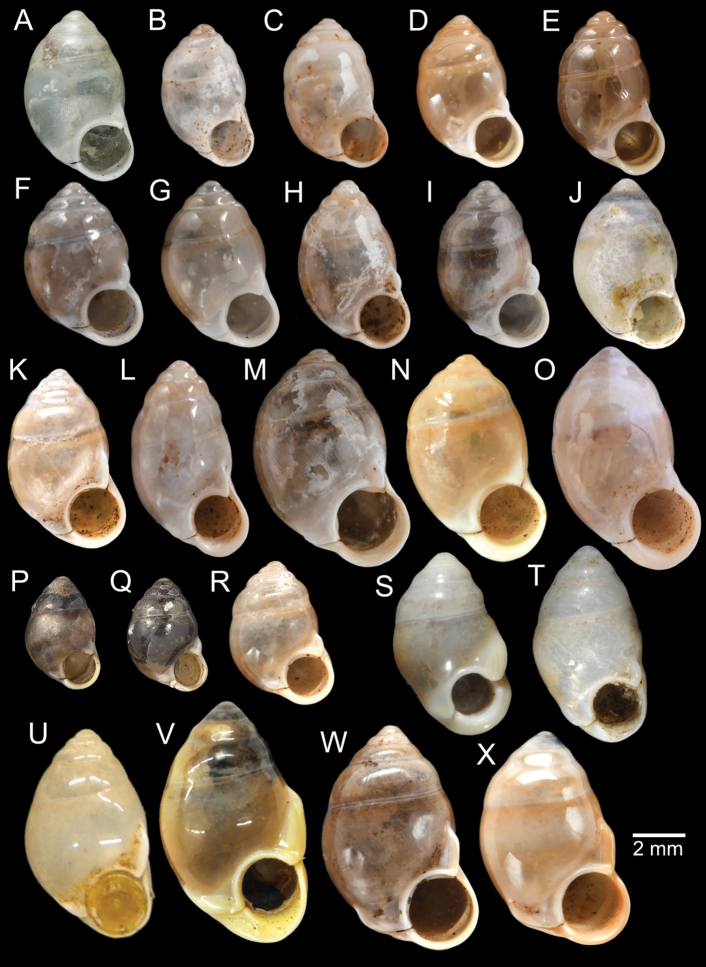
Shells of *Pupinaartata* species group from mainland Southeast Asia **A–M***Pupinaartata***A** syntype UMZC I.102960.A and specimens **B**CUMZ 12011 **C**CUMZ 12023 **D**CUMZ 12038 **E**CUMZ 12026 **F**CUMZ 12015 **G**CUMZ 12009 **H**CUMZ 12001 **I**CUMZ 12003 **J**NHMUK 1906.4.4.28 **K**CUMZ 12037 **L**CUMZ 12031, and **M**CUMZ 12018 **N, O***Pupinapallens***N** lectotype SMF 109951 and **O** specimen CUMZ 12042 **P–R***Pupinalimitanea***P, Q** syntypes NHMUK 1903.7.1.2967 and **R** specimen CUMZ 12044 **S***Pupinabilleti*, holotype MNHN-IM-2000-35841 **T***Pupinaverneaui*, syntype MNHN-IM-2000-35843 **U, V***Pupinahungerfordiana*, specimens NHMUK 91.3.14.686–7 **W, X***Pupinabensoni* sp. nov. **W** holotype CUMZ 12045/1 and **X** paratype CUMZ 12046/1. Photo: H. Taylor, NHM (**A, P, Q**), P. Maestrati, MNHN (**S, T**).

This species group from mainland Southeast Asia contains seven species, including three nominal species and one new species (*P.bensoni* sp. nov.) from Thailand. The distribution of the *P.artata* species group in Thailand is provided in Fig. 20. A synoptic view of all species within the *P.artata* species group from mainland Southeast Asia is given in Fig. 21 to provide the comparative size.

##### ﻿ Pupina artata

Taxon classificationAnimaliaArchitaenioglossaPupinidae

Benson, 1856

38446D96-E878-5C33-B44B-278839E24F7B

Figs 8E–H , 10B , 18A , 21A–M , 22 , 23 , 24A , 25A–C

Pupina artata Benson, 1856: 230. Type locality: Moulmein [Mawlamyine, Mawlamyine Township, Mawlamyine District, Mon State, Myanmar]. Theobald 1858 [1857]: 247, 248. Pfeiffer 1860: 142, pl. 37, figs 10–12. Sowerby I 1866: Pupinidae, pl. 3 (pl. 265), Pupina, figs 1, 2. Hanley and Theobald 1870: 4, pl. 7, fig. 5. Stoliczka 1871: 151, 152. Nevill 1878: 299, 300, Ava [Mandalay Region, Myanmar]; Moulmein; Buket Pondong [Gunung Pondok, Perak State, Malaysia]. Reeve 1878: Pupinidae, pl. 1, sp. 3. Crosse 1879: 340. de Morgan 1885: 413, Boukit Pondong, Pérak; Java [doubtful]; Moulmein; Lahat, Ipoh, Gôping, Kinta [Perak State, Malaysia]. von Möllendorff 1894: 155, the Samui Islands, Gulf of Siam [Samui Island, Surat Thani Province, Thailand]. Godwin-Austen 1897: 38, 39, pl. 69, fig. 6, 6a, b. Fischer and Dautzenberg 1904: 431, Ile Samui, golfe de Siam [Samui Island, Surat Thani Province, Thailand]. van Benthem Jutting 1960: 12, a hill near the hot springs, near Tandjong Rambutan, N.E. of Ipoh, Perak. Solem 1966: 12, Chieng Dao, Doi Sutep [Chiang Dao District and Doi Suthep Mountain, Chiang Mai Province, Thailand]. Davison 1995: 236, 237, limestone island C, Temengor dam, Perak, Malaysia. Chan 1998b: 2, Ipoh, Perak. Maassen 2001: 39, 40. BEDO 2017: 87. Sutcharit et al. 2018: fig. 5–13d.
Pupina artata var. blanfordiana Nevill, 1878: 300. Type locality: Thyet Myo [Thayetmyo, Magway Region, Myanmar]; Akoutong [Akauk Taung, Pyay District, Bago Region, Myanmar]; Kamah Hill, Tongoop, & c., Arakan [Toungup, Thandwe District, Rakhine State, Myanmar]; Prome [Pyay, Bago Region, Myanmar].
Pupina peguensis [non Benson]—Godwin-Austen 1897: 40, pl. 69, fig. 3, 3a–d, Kama on the right bank of the Irrawaddy, Pegu [Kamma Township, Thayet District, Magway Region, Myanmar]. BEDO 2017: 93.
Pupina (Tylotoechus) artata —Kobelt 1902: 306, 307. Gude 1921: 193. Laidlaw 1928: 33.
Pupina (Pupina) artata —Hemmen and Hemmen 2001: 39.
Pupina blanfordi [non Theobald]—BEDO 2017: 89.
Pupina limitanea [non Godwin-Austen]—BEDO 2017: 90. Sutcharit et al. 2018: fig. 5–13g.
Pupina sp.—Sutcharit et al. 2018: fig. 5–11a.

###### Type material examined.

**Figure 22.**
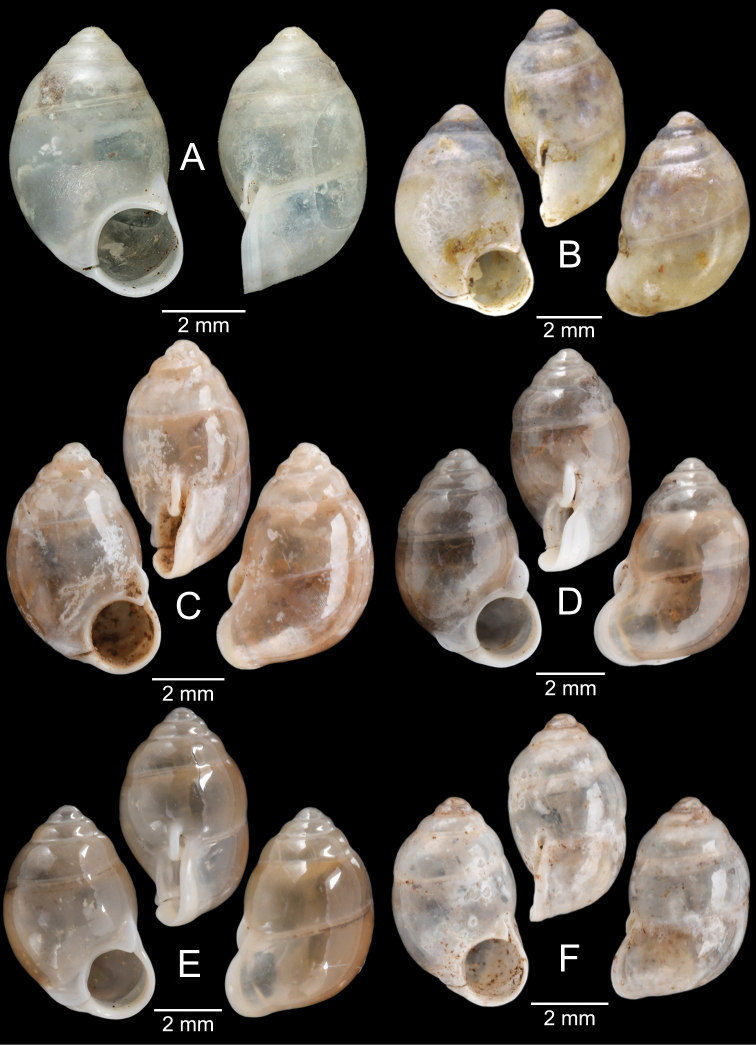
*Pupinaartata***A** syntype UMZC I.102960.A from Moulmein **B** specimen NHMUK 1906.4.4.28 from Moulmein **C** specimen CUMZ 12001 from Khao Tham Phra Temple, Chiang Rai **D** specimen CUMZ 12003 from Ban Ping Khong, Chiang Mai **E** specimen CUMZ 12009 from Phu Sang Waterfall, Phayao, and **F** specimen CUMZ 12011 from Thep Sathaporn Temple, Nakhon Sawan. Photo: H. Taylor, NHM (**A**).

***Syntype***UMZC I.102960.A (1 shell; Figs 21A, 22A) from the R. McAndrew collection, labelled “Bens. col., Moulmein”.

###### Other material examined.

**Figure 23.**
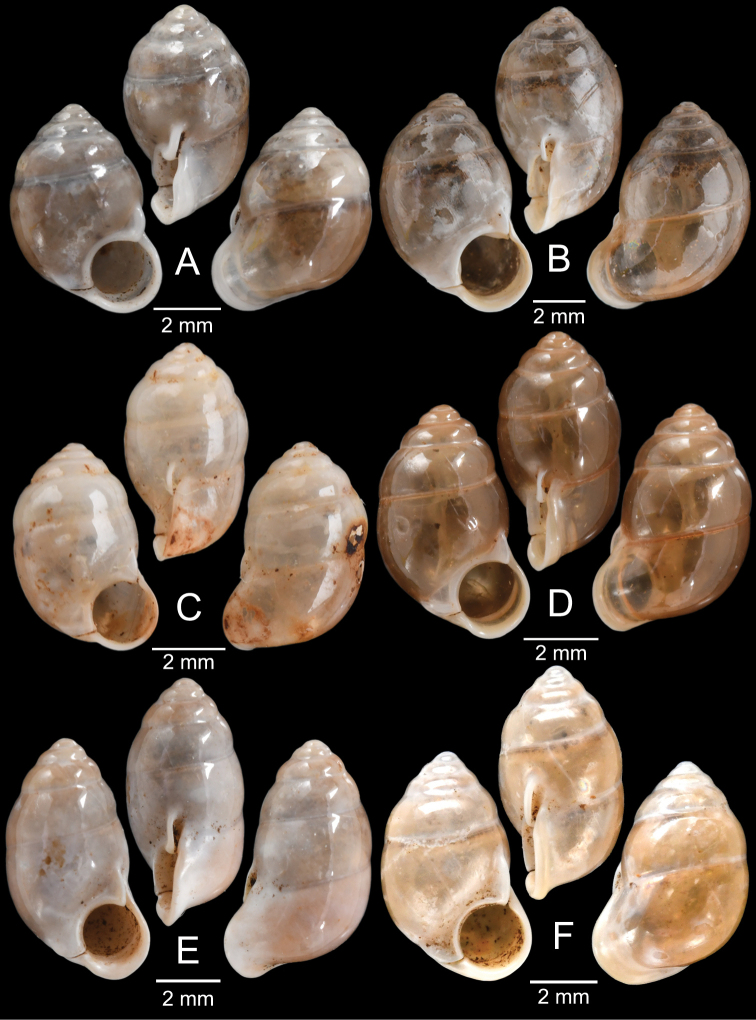
*Pupinaartata*: specimens **A**CUMZ 12015 from Khao Wong Phrommachan Temple, Uthai Thani **B**CUMZ 12018 from Tham Khao Thalu Temple, Ratchaburi **C**CUMZ 12023 from Tham Khiriwong Temple, Prachub Kirikhan **D**CUMZ 12026 from Tham Khao Cha Ang On Temple, Chonburi **E**CUMZ 12031 from Tham Khao Chakan Temple, Sa Kaeo, and **F**CUMZ 12037 from Wua Ta Lap Island, Surat Thani.

**Figure 24.**
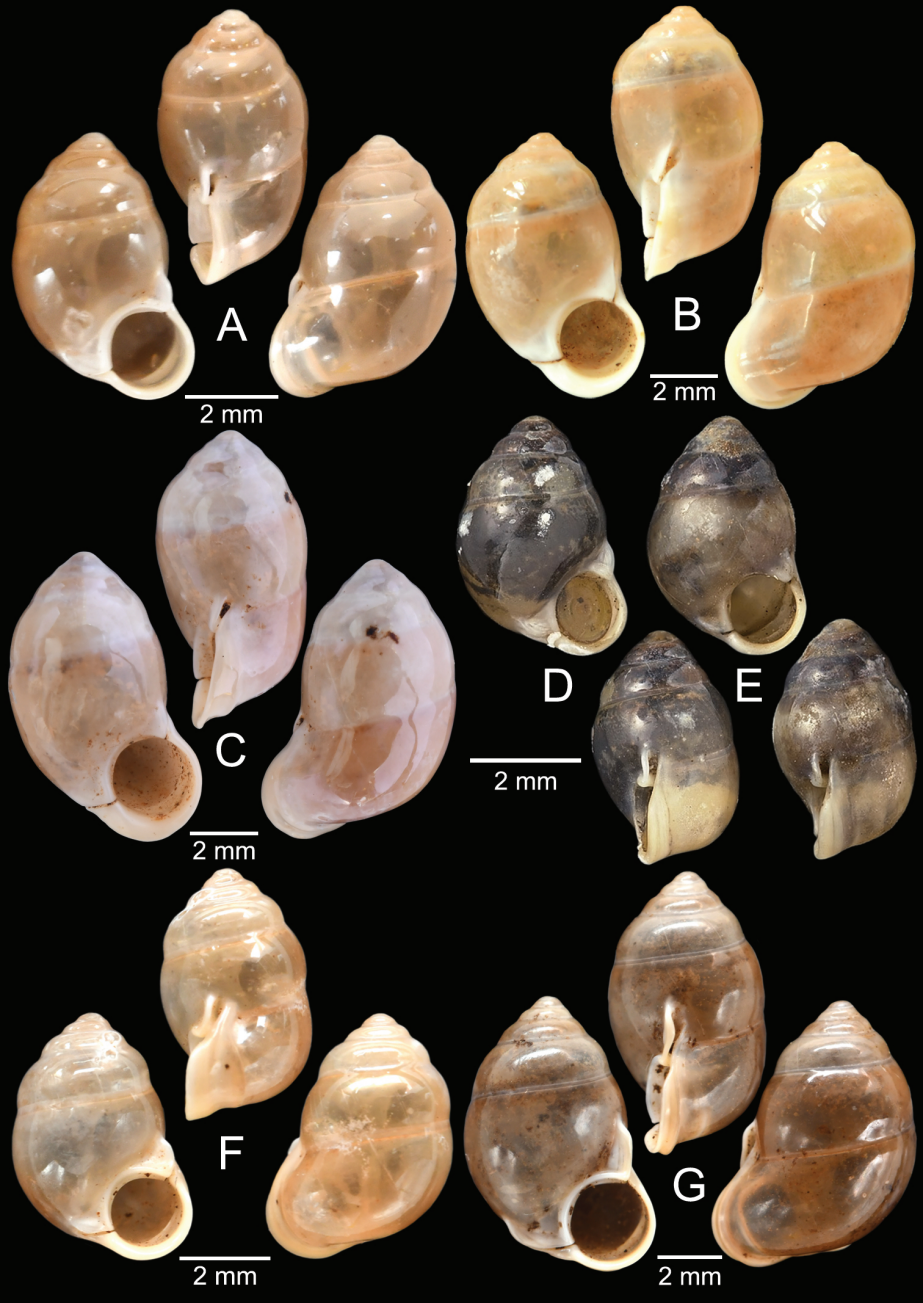
**A***Pupinaartata*, specimen CUMZ 12038 from Tham Suea Temple, Krabi **B, C***Pupinapallens***B** lectotype SMF 109951 and **C** specimen CUMZ 12042 from Suan Wiwek Bureau of Monks, Prachuap Khiri Khan **D–F***Pupinalimitanea***D, E** syntypes NHMUK 1903.7.1.2967 from East of Burma & Siam and **F** specimen CUMZ 12044 from Mae Lana junction, Mae Hong Son **G***Pupinabensoni* sp. nov., holotype CUMZ 12045/1. Photo: H. Taylor, NHM (**D, E**).

**Figure 25.**
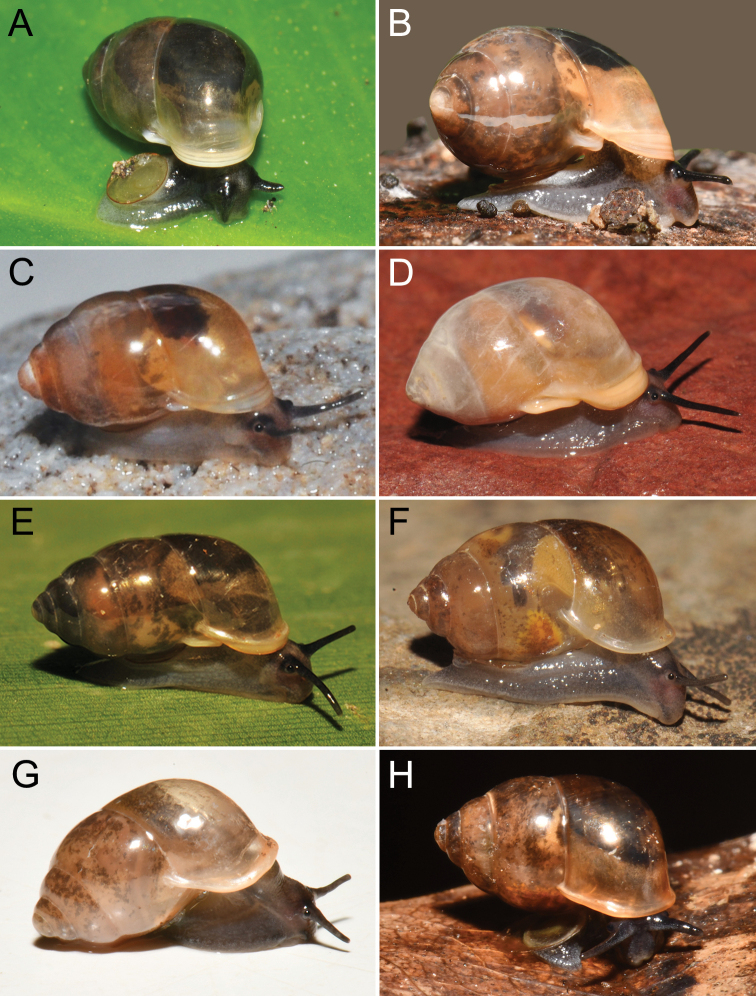
Live specimens of **A–C***Pupinaartata*: specimens **A**CUMZ 12020 from Buri Ratchawanaram Temple, Ratchaburi **B**CUMZ 12022 from Golden Dragon Cave, Ratchaburi, and **C**CUMZ 12037 from Wua Ta Lap Island, Surat Thani **D, E***Pupinabensoni* sp. nov. **D** paratype CUMZ 12045/2 from Khao Wong Cave, Uthai Thani and **E** specimen CUMZ 12047 from Tham Namthip Bureau of Monks, Uthai Thani **F, G***Pupinapeguensis*: specimens **F**CUMZ 12050 from Chai Thong Wararam Temple, Nakhon Sawan and **G**CUMZ 12051 from Tham Saeng Wiset Bureau of Monks, Nakhon Sawan **H***Pupinasiamensis*, specimen CUMZ 12069 from Khao Chi Chan Buddha Image, Chonburi. All not to scale.

NHMUK 1906.4.4.28 (6 shells; Figs 21J, 22B) from Moulmein, Myanmar. CUMZ 12001 (7 shells; Figs 21H, 22C) from Khao Tham Phra Temple, Mueang Chiang Rai District, Chiang Rai Province, 9 Jan. 2008. CUMZ 12002 (1 shell) from Luang Cave, Mae Sai District, Chiang Rai Province, 23 Oct. 2015. CUMZ 12003 (21 shells; Figs 10B, 18A, 21I, 22D) from Ban Ping Khong, Chiang Dao District, Chiang Mai Province, 8 Oct. 2008. CUMZ 12193 (4 shells) from Ban Ping Khong, Chiang Dao District, Chiang Mai Province, 21 Nov. 2012. CUMZ 12190 (3 shells) from Chiang Dao Cave, Chiang Dao District, Chiang Mai Province, 25 Oct. 2015. CUMZ 12004 (4 specimens in ethanol) from Bua Tong Cave, Mae Tang District, Chiang Mai Province, 8 Oct. 2017. CUMZ 12168 (4 shells) from Doi Ang Khang, Fang District, Chiang Mai Province, 24 Oct. 2015. CUMZ 12005 (43 shells) from Pha Daeng Cave, Mueang Mae Hong Son District, Mae Hong Son Province, 18 Jan. 2015. CUMZ 12006 (17 shells and 1 specimen in ethanol; Fig. 8E) from Pha Daeng Cave, Mueang Mae Hong Son District, Mae Hong Son Province, 3 Dec. 2020. CUMZ 12007 (7 shells) from Tham Nam Pha Pha Ngam Temple, Mae Phrik District, Lampang Province, 7 Jan. 2008. CUMZ 12008 (3 shells and 4 specimens in ethanol; Fig. 8F) from Tham Nam Pha Pha Ngam Temple, Mae Phrik District, Lampang Province, 8 Oct. 2020. CUMZ 12009 (12 shells; Figs 21G, 22E) from Phu Sang Waterfall, Phu Sang District, Phayao Province, 24 Oct. 2008. CUMZ 12010 (5 shells) from Phu Sang Waterfall, Phu Sang District, Phayao Province, 19 Nov. 2012. CUMZ 12011 (12 shells and 7 specimens in ethanol; Figs 21B, 22F) from Thep Sathaporn Temple, Banphot Phisai District, Nakhon Sawan Province, 17 July 2008. CUMZ 12012 (17 shells) from Khao Chuak Charoentham Temple, Ban Rai District, Uthai Thani Province, 8 July 2009. CUMZ 12013 (3 shells) from Khao Chuak Charoentham Temple, Ban Rai District, Uthai Thani Province, 27 Aug. 2016. CUMZ 12014 (1 shell) from Khao Chuak Charoentham Temple, Ban Rai District, Uthai Thani Province, 5 Dec. 2020. CUMZ 12015 (14 shells; Figs 21F, 23A) from Khao Wong Phrommachan Temple, Ban Rai District, Uthai Thani Province, 8 July 2009. CUMZ 12016 (5 shells) from Tham Prathat Mueang Thep Temple, Ban Rai District, Uthai Thani Province, 5 Dec. 2020. CUMZ 12017 (13 shells) from Krasae Cave, Sai Yok District, Kanchanaburi Province, 10 Dec. 2006. CUMZ 12173 (14 shells) from Tham Charoentham Temple, Mueang Kanchanaburi District, Kanchanaburi Province, 19 Aug. 2020. CUMZ 12192 (1 shell) from Ban Tapoepu-Wakruko, Umphang District, Tak Province, 30 June 2015. CUMZ 12018 (68 shells and 10 specimens in ethanol; Figs 21M, 23B) from Tham Khao Thalu Temple, Chom Bueang District, Ratchaburi Province, 9 Dec. 2006. CUMZ 12019 (21 shells) from Tham Khao Thalu Temple, Chom Bueang District, Ratchaburi Province, 9 Dec. 2009. CUMZ 12020 (4 shells and 6 specimens in ethanol; Fig. 25A) from Buri Ratchawanaram Temple, Pak Tho District, Ratchaburi Province, 8 May 2017. CUMZ 12021 (20 shells and 1 specimen in ethanol) from Buri Ratchawanaram Temple, Pak Tho District, Ratchaburi Province, 18 Aug. 2020. CUMZ 12022 (5 specimens in ethanol; Fig. 25B) from Golden Dragon Cave, Pak Tho District, Ratchaburi Province, 18 Aug. 2019. CUMZ 12023 (17 shells; Figs 21C, 23C) from Tham Khiriwong Temple, Bang Saphan District, Prachub Kirikhan Province, 21 Apr. 2007. CUMZ 12024 (147 shells) from Tham Khiriwong Temple, Bang Saphan District, Prachub Kirikhan Province, 29 July 2019. CUMZ 12169 (10 shells) from Tham Thep Nimit Temple, Pak Chong District, Nakhon Ratchasima Province, 24 Aug. 2020. CUMZ 12025 (28 shells) from Tham Khao Cha Ang On Temple, Bo Thong District, Chonburi Province, 13 Mar. 2006. CUMZ 12026 (28 shells and 23 specimens in ethanol; Figs 21E, 23D) from Tham Khao Cha Ang On Temple, Bo Thong District, Chonburi Province, 17 Aug. 2006. CUMZ 12028 (34 shells) from Bo Thong District, Chonburi Province, 9 May 2008. CUMZ 12027 (2 specimens in ethanol) from Phromawat Temple, Si Racha District, Chonburi Province, 19 Sept. 2020. CUMZ 12174 (1 shell) from Tham Khao Loi Temple, Khao Chamao District, Rayong Province, 23 Oct. 2010. CUMZ 12029 (85 shells and 10 specimens in ethanol; Fig. 8G, H) from Khao Tham Raet Temple, Tha Takiap District, Chachoengsao Province, 21 May 2012. CUMZ 12030 (3 shells) from Khao Tham Raet Temple, Tha Takiap District, Chachoengsao Province, 1 Mar. 2018. CUMZ 12031 (43 shells; Figs 21L, 23E) from Tham Khao Chakan Temple, Khao Chakan District, Sa Kaeo Province, 7 Apr. 2000. CUMZ 12032 (7 specimens in ethanol) from Tham Khao Chakan Temple, Khao Chakan District, Sa Kaeo Province, 25 Feb. 2018. CUMZ 12033 (2 shells) from Tham Khao Maka Temple, Mueang Sa Kaeo District, Sa Kaeo Province, 2 Nov. 2008. CUMZ 12034 (2 shells) from Khao Pha Pheung Temple, Klong Had District, Sra Keo Province, 21 May 2018. CUMZ 12035 (1 specimen in ethanol) from Na Mueang Waterfall, Ko Samui District, Surat Thani Province, 4 Mar. 2007. CUMZ 12036 (10 shells) from Wua Ta Lap Island, Ko Samui District, Surat Thani Province, 5 Mar. 2007. CUMZ 12037 (1 shell and 2 specimens in ethanol; Figs 21K, 23F, 25C) from Wua Ta Lap Island, Ko Samui District, Surat Thani Province, 6 June 2009. CUMZ 12038 (4 shells; Figs 21D, 24A) from Tham Suea Temple, Mueang Krabi District, Krabi Province, 6 Oct. 2006. CUMZ 12039 (4 shells) from Khao Noi Phothiyan Temple, Mueang Satul District, Satul Province, 31 Aug. 2015. CUMZ 12040 (1 shell) from Khao Rup Chang, Mueang Songkhla District, Songkhla Province, 23 Jan. 2007.

###### Diagnosis.

Shell ovate; last whorl ca. three quarters of shell height. Apertural lip slightly thickened, not expanded. Both parietal and columellar teeth fin-shaped and slightly thickened; parietal tooth covering posterior canal; columellar tooth next to slit-like anterior canal.

###### Differential diagnosis.

*Pupinaartata* is most similar to *P.pallens* and *P.limitanea* in shell shape, but different from *P.pallens* in that the basal position of the apertural lip is not widened, and different from *P.limitanea* by a longer last whorl, and parietal and columellar teeth and apertural lip less thickened.

###### Distribution.

Peninsular Malaysia, Myanmar (Laidlaw 1928; Solem 1966), and throughout Thailand except in the northeastern region.

###### Remarks.

The type specimen of *P.artatablanfordiana* could not be located, so the validity of this subspecies is still unknown. The specimen identified as *P.peguensis* and figured in Godwin-Austen (1897: pl. 69, fig. 3, 3a–d) from Kama on the right bank of the Irrawaddy River, Pegu is different from the holotype of *P.peguensis* (see Tripathy and Sajan 2019), but similar to the type specimen of *P.artata*. Thus, this specimen is herein identified as *P.artata*.

The specimen of *P.artata* figured in Maassen (2002: text-fig. 3) from Sumatra should constitute a different species as it is different from the syntype figured here in having a smaller, sharper parietal tooth revealing the posterior canal and an ear-lobe-shaped columellar tooth covering the anterior canal. Thus, those specimens should belong to the *P.arula* species group instead (see below).

All specimens from Thailand with a slightly thickened, fin-shaped parietal tooth covering the posterior canal are herein identified as *P.artata*. However, these specimens exhibit a variable shell size (smaller with shell height 5.4 mm, shell width 3.5 mm, to larger with shell height 8.4 mm; shell width 5.9 mm; Fig. 21A–M). The shell shape is also variable from ovate which is similar to the syntype (Fig. 21A), to more globose (Fig. 21F) or more elongate (Fig. 21L). In addition, these specimens exhibit a variation in length, outer curvature and thickness of the parietal tooth, and body colour. There is also a case of different shell colour morphs (brown and grey) within the same population (Fig. 8G, H). Therefore, DNA data is needed to reveal the extent of genetic differentiation or cryptic diversity within the *P.artata* morphotype.

##### ﻿ Pupina pallens

Taxon classificationAnimaliaArchitaenioglossaPupinidae

Möllendorff, 1894

39E4A79A-ABF5-51FD-A178-E60435341183

Figs 21N, O , 24B, C

Pupina pallens Möllendorff, 1894: 155, pl. 16, figs 27, 28. Type locality: Samui Islands, Gulf of Siam [Samui Island, Surat Thani Province, Thailand]. Fischer and Dautzenberg 1904: 431. BEDO 2017: 92. Sutcharit et al. 2018: fig. 5–13i.
Pupina (Tylotoechus) pallens —Kobelt 1902: 318, 319. Laidlaw 1928: 34. Zilch 1957: 47, pl. 2, fig. 16. Hemmen and Hemmen 2001: 39.

###### Type material examined.

***Lectotype***SMF 109951 (Figs 21N, 24B) and paralectotypes SMF 109952 (4 shells), SMF 109953 (2 shells) from Golf von Siam: Koh Samui.

###### Other material examined.

CUMZ 12041 (1 shell) from Bang Phu Temple, Sam Roi Yot District, Prachuap Khiri Khan Province, 19 Oct. 2020. CUMZ 12042 (14 shells; Figs 21O, 24C) from Suan Wiwek Bureau of Monks, Sam Roi Yot District, Prachuap Khiri Khan Province, 21 Oct. 2020.

###### Diagnosis.

Shell ovate; last whorl ca. three quarters of shell height. Apertural lip slightly thickened, not expanded; basal position widened. Both parietal and columellar teeth fin-shaped and slightly thickened; parietal tooth covering posterior canal; columellar tooth next to slit-like anterior canal.

###### Differential diagnosis.

*Pupinapallens* can be distinguished from all other species in the *P.artata* species group from mainland Southeast Asia by the widened basal position of the apertural lip.

###### Distribution.

The type locality (Laidlaw 1928) and Prachuap Khiri Khan Province, western Thailand.

###### Remarks.

von Möllendorff (1894) stated that this species is different from *P.arula* in having “the more obtuse spire, the more distorted last whorl, and consequently the aperture placed more to the right and protracted at the base, the thinner outer peristome, the broader columella, the broad triangular parietal lamella, and the narrower lower incision”. More sampling of this species, with both morphometric and molecular phylogenetic analyses, are needed to resolve the relationship between *P.pallens* and other species in the *P.artata* species group.

##### ﻿ Pupina limitanea

Taxon classificationAnimaliaArchitaenioglossaPupinidae

Godwin-Austen, 1897

1EF92976-4F0F-55C7-93DB-99C6A125930A

Figs 21P–R , 24D–F

Pupina limitaneus [sic] Godwin-Austen, 1897: 40, pl. 69, fig. 4, 4a, b. Type locality: Eastern frontier of Burmah and Siam; Eastern Shan Plateau [Shan State, Myanmar].
Pupina (Tylotoechus) limitanea —Kobelt 1902: 316, 317. Gude 1921: 196. Hemmen and Hemmen 2001: 39.
Pupina brachysoma [non Ancey]—Inkhavilay et al. 2019: 29, fig. 15f, Nam Ork Roo, Ban Nathong village, Namo District, Oudomxay Province.

###### Type material examined.

***Syntypes***NHMUK 1903.7.1.2967 (10 shells; Figs 21P, Q, 24D, E) from East of Burma & Siam.

###### Other material examined.

CUMZ 12043 (1 specimen in ethanol) from Pha Tub Cave, Mueang Nan District, Nan Province, 11 Oct. 2009. CUMZ 12171 (1 shell) from Luang Sakoen Cave, Song Khwae District, Nan Province, 19 Jan. 2017. CUMZ 12044 (2 shells; Figs 21R, 24F) from Mae Lana junction, Pang Mapha District, Mae Hong Son Province, 18 Jan. 2015.

###### Diagnosis.

Shell ovate; last whorl ca. 60% of shell height. Apertural lip highly thickened, not expanded. Both parietal and columellar teeth fin-shaped and very thickened; parietal tooth always covering posterior canal; columellar tooth either next to or covering slit-like anterior canal.

###### Differential diagnosis.

*Pupinalimitanea* is most similar to *P.artata* in shell shape, but differs in having parietal and columellar teeth and apertural lip thickened, and a shorter last whorl.

###### Distribution.

Eastern Myanmar, Laos (Godwin-Austen 1897; Inkhavilay et al. 2019), and Nan Province, northern Thailand.

###### Remarks.

The specimen of *P.brachysoma* from Oudomxay Province, Laos figured in Inkhavilay et al. (2019: fig. 15f) is different from the type materials of *P.brachysoma* (see below) in having a thick and large parietal tooth covering the posterior canal, whereas *P.brachysoma* has a sharp triangular parietal tooth which is not thickened, making the posterior canal visible. Therefore, the specimen from Oudomxay Province, Laos is herein identified as *P.limitanea* of the *P.artata* species group, whereas *P.brachysoma* belongs to the *P.aureola* species group.

As this species is highly similar to *P.artata*, more sampling of this species, with both morphometric and molecular phylogenetic analyses, are needed to resolve the relationship between these two species.

##### ﻿ Pupina bensoni

Taxon classificationAnimaliaArchitaenioglossaPupinidae

Jirapatrasilp sp. nov.

A426991E-39C3-5FA7-BADC-AAEEEFA95C44

https://zoobank.org/B493C554-4B2C-4910-809C-33297E1A6005

**Figure 26.**
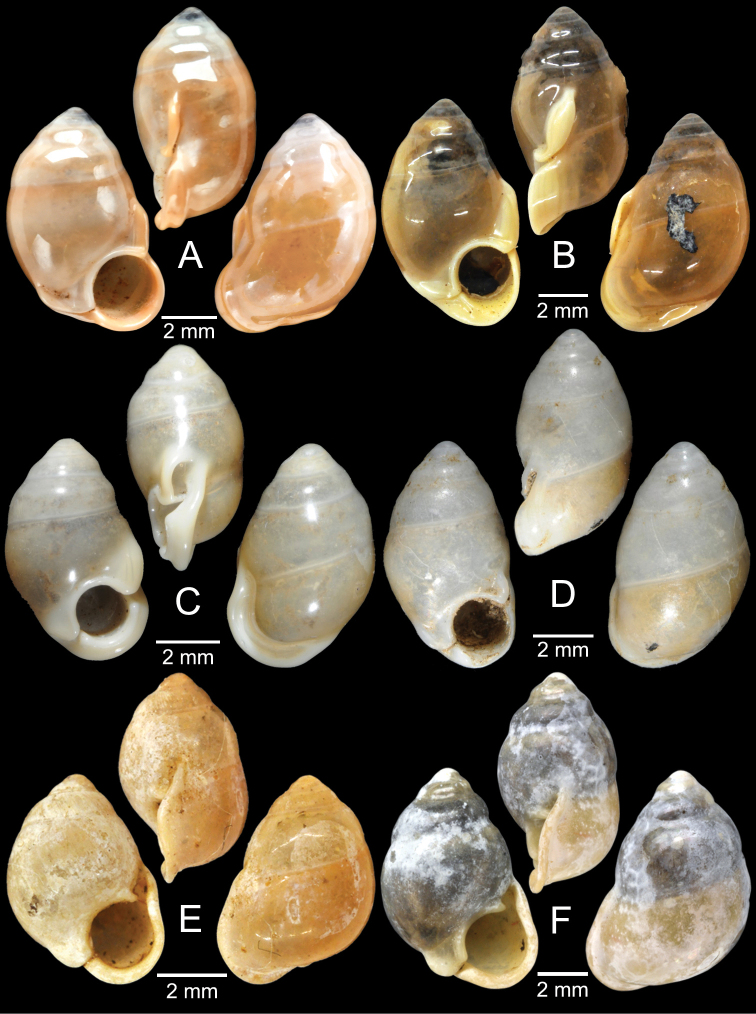
**A***Pupinabensoni* sp. nov., paratype CUMZ 12046/1 from Khao Wong Cave, Uthai Thani **B***Pupinahungerfordiana*, specimen NHMUK 91.3.14.686–7 from Hsaddan Koo **C***Pupinabilleti*, holotype MNHN-IM-2000-35841 **D***Pupinaverneaui*, syntype MNHN-IM-2000-35843 from Ha-Giang, Tonkin **E, F***Pupinapeguensis***E** syntype of *Pupinablanfordi*NHMUK 1888.12.4.100 from Pegu and **F** specimen NHMUK ex. Cuming coll. from Lao Mountains, Camboja. Photo: P. Maestrati, MNHN (**C, D**).

Figs 19A , 21W, X , 24G , 25D, E , 26A

###### Type material.

***Holotype***CUMZ 12045/1 (Figs 21W, 24G), 5 June 2017, coll. C. Sutcharit, R. Srisonchai, A. Pholyotha. Measurement: shell height 8.5 mm, shell width 5.9 mm and 5½ whorls. ***Paratypes***CUMZ 12045/2–10 (7 shells and 2 specimens in ethanol; Fig. 25D) and NHMUK 20210333 (2 shells), same data as holotype; CUMZ 12046, 5 Dec. 2020, coll. P. Jirapatrasilp, C. Sutcharit, A. Pholyotha (14 shells and 2 specimens in ethanol; Figs 21X, 26A), from the type locality.

###### Type locality.

Khao Wong Cave, Ban Rai District, Uthai Thani Province, Thailand, 15°01'53.1"N, 99°27'21.0"E, 246 m asl.

###### Other material examined.

CUMZ 12047 from Tham Namthip Bureau of Monks, Lan Sak District, Uthai Thani Province, 28 July 2016 (8 shells and 12 specimens in ethanol; Figs 19A, 25E). CUMZ 12048 from Tham Namthip Bureau of Monks, Lan Sak District, Uthai Thani Province, 5 Dec. 2020 (7 shells and 7 specimens in ethanol).

###### Diagnosis.

Shell ovate; last whorl ca. two thirds of shell height. Apertural lip thickened, not expanded to slightly expanded; with a furrow between inner and outer peristomes; inner peristome thickened and cord-like. Parietal tooth thickened, long trapezoid shaped, reaching beyond the middle of last whorl, outer border nearly straight, always covering posterior canal; columellar tooth thickened, curvedly triangular shaped, located next to slit-like anterior canal.

###### Differential diagnosis.

*Pupinabensoni* sp. nov. is most similar to *P.hungerfordiana* in having a long parietal tooth reaching beyond the middle of last whorl, but differs in the long, trapezoid shape of parietal tooth, with the outer border nearly straight, and a furrow between inner and outer peristomes, with the inner peristome thickened and cord-like.

###### Description.

Shell height 7.0–8.6 mm; shell width 4.0–6.0 mm. Shell ovate, solid, semi-transparent, whitish to brown, devoid of prominent sculpture on glazed smooth surface. Apex obtuse. Growth lines on shell surface inconspicuous. Whorl 5½–6, last whorl large ca. two-thirds of shell height. Spire angle ca. 90°; somewhat extended. Sutures slightly impressed, but shallow. Aperture circular; lip thickened with paler colour (ca. 0.2–0.3 mm wide and 0.5–0.6 mm thick), not expanded to slightly expanded. Apertural lip with a furrow between inner and outer peristomes, with inner peristome thickened and cord-like. Parietal callus sharply defined and thickened with paler colour. Peristome interrupted by two canals; posterior canal ca. 1.5 mm long and 0.3 mm at its widest, continuing slightly obliquely forming narrow groove bordered by parietal tooth and extended part of apertural lip; anterior canal curved and slit-liked continuing horizontally ca. 1.7 mm. Parietal tooth thickened, long trapezoid shaped (ca. 2.0 mm high, 0.7 mm wide and 0.3 mm thick), outer border somewhat straight, located at angular corner of aperture, extending beyond apertural lip and reaching beyond the middle of last whorl, always covering posterior canal. Columellar tooth somewhat thickened, curvedly triangular shaped (ca. 0.9 mm high, 2.2 mm long and 0.3 mm thick), located next to anterior canal. Umbilicus closed. Operculum round, yellowish, transparent corneous with smooth edge.

###### Etymology.

The specific epithet is dedicated to W.H. Benson, an Irish malacologist, who made large collections of molluscs and described numerous species from India and Myanmar, especially the two oldest *Pupina* species from this region.

###### Distribution.

This new species is found from Uthai Thani Province, central Thailand.

#### Species of group I (*P.artata* species group) from other parts of mainland Southeast Asia not recorded for Thailand

##### ﻿ Pupina hungerfordiana

Taxon classificationAnimaliaArchitaenioglossaPupinidae

Nevill, 1878

8BDDA5A9-CED5-5F05-944C-8ABB749B5AB4

Figs 21U, V , 26B

Pupina hungerfordiana Nevill, 1878: 300, 301. Type locality: Hsaddan Koo, Salween Valley [Hasaddan Koo, the cave on the limestone hill south of Hpa-An in Ein Du Village, Hpa-An Township, Hpa-An District, Kayin State, Myanmar]. Nevill 1881: 148, pl. 6, fig. 6.
Pupina hungerfordi [sic]—Godwin-Austen 1897: 41, 42, pl. 69, fig. 7, 7a.
Pupina (Tylotoechus) hungerfordiana —Kobelt 1902: 314. Gude 1921: 194, 195.

###### Type material examined.

***Holotype*** of *Pupinahungerfordiana* figured in Nevill (1881: pl. 6, fig. 6).

###### Other material examined.

NHMUK 91.3.14.686–7 (2 shells; Figs 21U, V, 26B) from Hsaddan Koo.

###### Diagnosis.

Shell ovate; last whorl ca. two thirds of shell height. Apertural lip thickened. Parietal tooth thickened, long fin-shaped, reaching beyond the middle of last whorl, outer border curved, covering posterior canal; columellar tooth somewhat thickened, curvedly triangular shaped, located next to slit-like anterior canal.

###### Differential diagnosis.

*Pupinahungerfordiana* is most similar to *P.artata* and *P.bensoni* sp. nov. in shell shape, but different from *P.artata* by the long, thickened, fin-shaped parietal tooth, reaching beyond the middle of last whorl, and different from *P.bensoni* sp. nov. by the lack of furrow between the inner and outer peristomes.

###### Distribution.

Known only from the type locality (Gude 1921).

###### Remarks.

As *P.hungerfordiana* was described based on a single specimen as explicitly stated in the original description, that specimen is the holotype fixed by monotypy (ICZN 1999: Art. 73.1.2).

##### ﻿ Pupina billeti

Taxon classificationAnimaliaArchitaenioglossaPupinidae

Fischer, 1898

AF634E30-201B-5FA8-9D59-A15E71291C04

Figs 21S , 26C

Pupina billeti Fischer, 1898: 333, 334, pl. 18, figs 38–41. Type locality: Rochers calcaires Déo-Ma-Phuc [limestone areas around Ma Phuc Pass, Tra Linh District, Cao Bang Province, Vietnam]. Fischer and Dautzenberg 1904: 431, Bac-Kan, Tonkin [Bac Kan Province, Vietnam].
Pupina (Tylotoechus) billeti —Kobelt 1902: 309.

###### Type material examined.

***Holotype***MNHN-IM-2000-35841 (Figs 21S, 26C) from Deo-Ma-Phuc.

###### Diagnosis.

Shell ovate; last whorl ca. 70% of shell height. Apertural lip extremely thickened; with a furrow between inner and outer peristomes; inner peristome thickened and cord-like; parietal callus distinct. Both parietal and columellar teeth extremely thickened; parietal tooth covering posterior canal; columellar tooth next to slit-like anterior canal.

###### Differential diagnosis.

*Pupinabilleti* can be distinguished from all other species in the *P.artata* species group from mainland Southeast Asia by having the thickest parietal and columellar teeth and apertural lip, and a distinct parietal callus.

###### Distribution.

Northern Vietnam (Fischer and Dautzenberg 1904).

###### Remarks.

As *P.billeti* was described based on a single specimen as explicitly stated in the original description, that specimen is the holotype fixed by monotypy (ICZN 1999: Art. 73.1.2).

##### ﻿ Pupina verneaui

Taxon classificationAnimaliaArchitaenioglossaPupinidae

Dautzenberg & Fischer, 1906

DC458A75-7277-5EAF-8521-11B683D72B88

Figs 21T , 26D

Pupina verneaui Dautzenberg & Fischer, 1906 [1905]: 440, 441, pl. 10, figs 13–15. Type locality: Ha-Giang [Ha Giang Province, Vietnam]. Fischer 1963: 34. Do et al. 2015: 126, fig. 6c, Son La Province, Vietnam.
Eupupina verneaui —Dautzenberg and Fischer 1908: 208, 209, Mo-Xat [west of Quang Uyen, Cao Bang Province, Vietnam]; Quang-Huyen [Quang Uyen, Cao Bang Province, Vietnam].

###### Type material examined.

***Syntypes***MNHN-IM-2000-35843 (Figs 21T, 26D) from Ha-Giang, Tonkin.

###### Diagnosis.

Shell ovate-fusiform; last whorl ca. 70% of shell height; suture very shallow. Apertural lip somewhat thickened, not expanded. Both parietal and columellar teeth fin-shaped and thickened; parietal tooth somewhat covering posterior canal; columellar tooth next to slit-like anterior canal.

###### Differential diagnosis.

*Pupinaverneaui* is most similar to *P.artata* in having fin-shaped and thickened teeth, but differs in having a more ovate-fusiform shell shape and a rather shallower suture.

###### Distribution.

Northern Vietnam (Do et al. 2015).

###### Remarks.

The specimen of *P.verneaui* figured in Inkhavilay et al. (2019: fig. 16a) from Ban Nong Kham village, Kasy District, Vientiane Province, Laos should constitute a different species as it is different from the syntype figured here in having a wider spire, a more bulging last whorl and a thinner and sharper parietal tooth.

#### Group II: *Pupinaarula* species group

Figs 10C, 18B, C, 19B

This species group is characterised by an indistinct to thick parietal tooth, extending from a parietal callus. When observed from lateral view, the parietal tooth continues horizontally. A columellar tooth is fin-shaped, or the outer margin is curved downward appearing as an earlobe shape covering an anterior canal. The anterior canal is either not visible or appears slit-like when observed from apertural view, where the anterior canal is as long as the apertural lip width. A posterior canal is always wide and curved outward, bulging at the outer margin, sometimes slit-like. An outer apertural lip is slightly curved (Fig. 18C) to sharply bent when observed from lateral view (Fig. 18B). An operculum is round, thin, multispiral, yellowish, transparent corneous, and sometimes with uneven edge.

**Figure 27.**
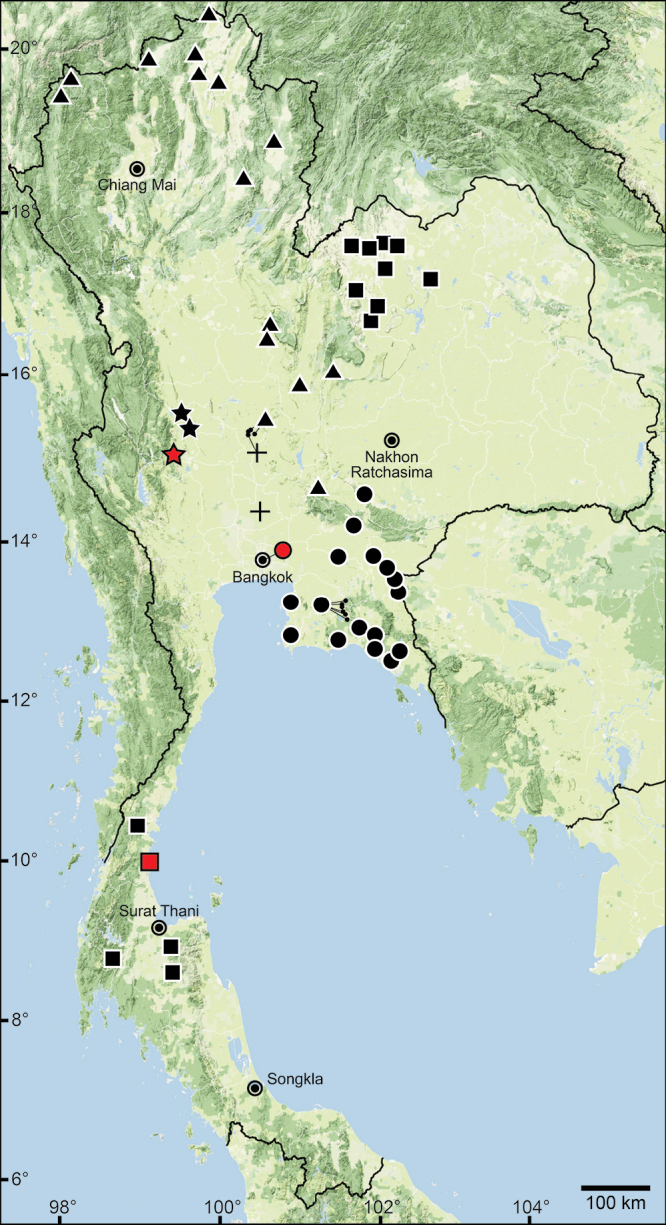
Distribution map of the *Pupinaarula* species group: *Pupinapeguensis* (triangle), *Pupinacrosseana* (plus sign), *Pupinasiamensis* (circle), *Pupinabilabiata* sp. nov. (square), and *Pupinagodwinausteni* sp. nov. (star). Each red symbol indicates the type locality of its respective taxon. The occurrences of *Pupinaarula* and *Pupinamouhoti* in northern Thailand are uncertain, thus their distributions are not mapped.

**Figure 28.**
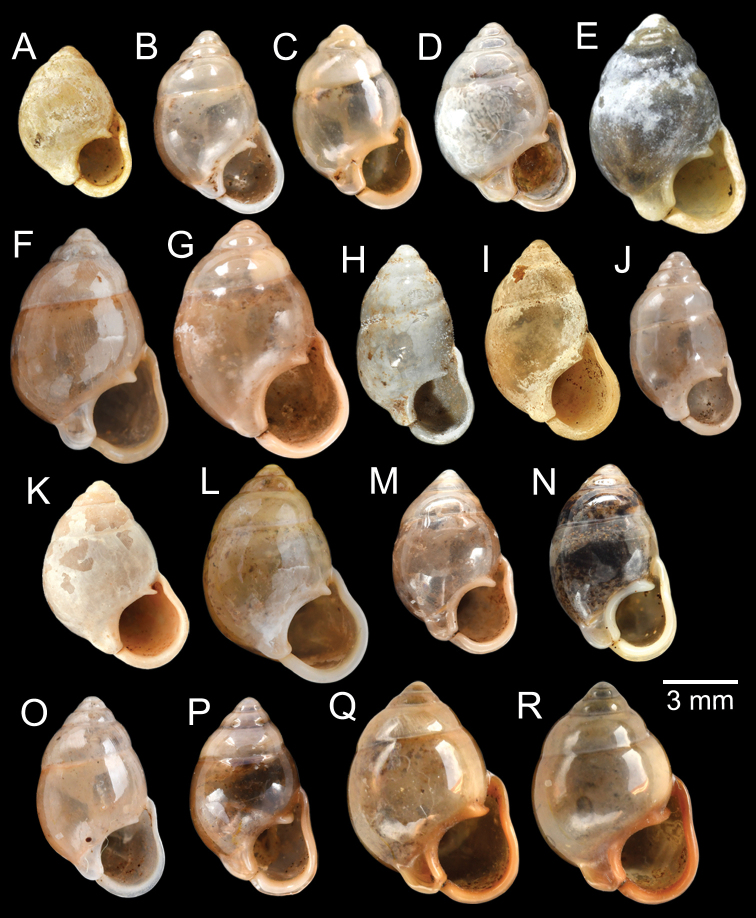
Shells of *Pupinaarula* species group from mainland Southeast Asia **A–G***Pupinapeguensis***A** syntype of *Pupinablanfordi*NHMUK 1888.12.4.100 and specimens **B**CUMZ 12105 **C**CUMZ 12050 **D**CUMZ 12103 **E**NHMUK ex. Cuming coll. **F**CUMZ 12094, and **G**CUMZ 12108 **H–J***Pupinacrosseana***H** lectotype MNHN-IM-2000-35834 **I** paralectotype RBINS MT966/10591, and **J** specimen CUMZ 12049 **K, L***Pupinasiamensis***K** lectotype SMF 109948 and **L** specimen CUMZ 12052 **M–P***Pupinabilabiata* sp. nov. **M** holotype CUMZ 12073/1 and specimens **N**CUMZ 12079 **O**CUMZ 12081, and **P**CUMZ 12086 **Q, R***Pupinagodwinausteni* sp. nov. **Q** holotype CUMZ 12090/1 and **R** paratype CUMZ 12090/2. Photo: P. Maestrati, MNHN (**H**), F. Trus, RBINS (**I**).

**Figure 29.**
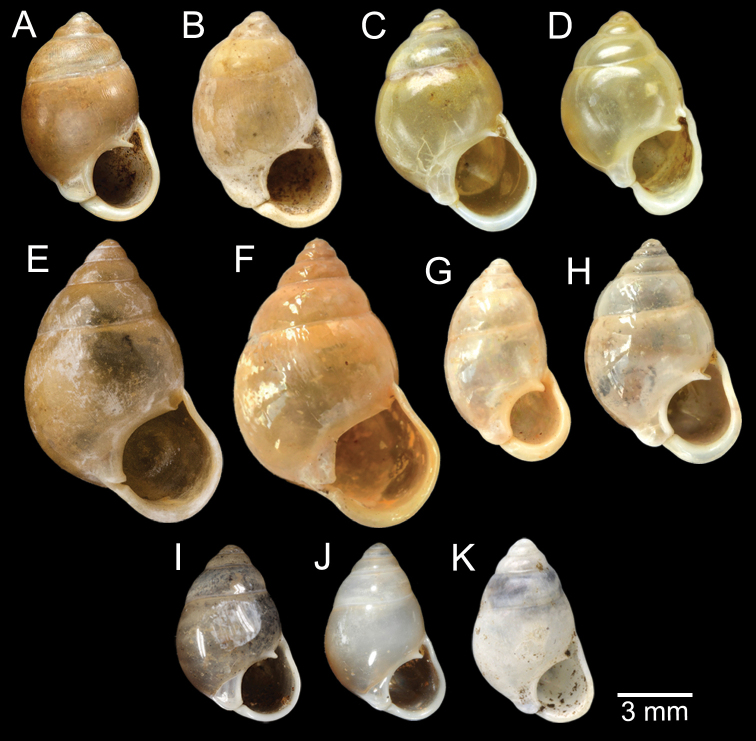
Shells of *Pupinaarula* species group from mainland Southeast Asia **A, B***Pupinaarula***A** syntype UMZC I.103025 and **B** specimen NHMUK 1888.12.4.109. **C, D***Pupinamouhoti*, possible syntypes NHMUK ex. Cuming coll. **E, F***Pupinavescoi***E** syntype NHMUK 1893.2.4.767 and **F** specimen SMF 109956/1 **G***Pupinaperakensis*, lectotype SMF 109969/1 **H***Pupinaexcisa*, lectotype SMF 110778/1 **I–K***Pupinaexclamationis***I, J** syntypes MNHN-IM-2000-35840 and **K** specimen NHMUK 1901.12.23.205 “forma minor”. Photo: J. Ablett, H. Taylor, NHM (**A**), A. Lardeur, P. Maestrati, MNHN (**I, J**).

This species group from mainland Southeast Asia contains 10 species, including five nominal species and two new species (*P.bilabiata* sp. nov. and *P.godwinasuteni* sp. nov.) from Thailand. The distribution of the *Pupinaarula* species group in Thailand is provided in Fig. 27. A synoptic view of all species within the *P.arula* species group from mainland Southeast Asia is given in Figs 28, 29 to provide the comparative size.

##### ﻿ Pupina peguensis

Taxon classificationAnimaliaArchitaenioglossaPupinidae

Benson, 1860

3ED45375-5B70-5B66-8887-18AF13B99A51

**Figure 30.**
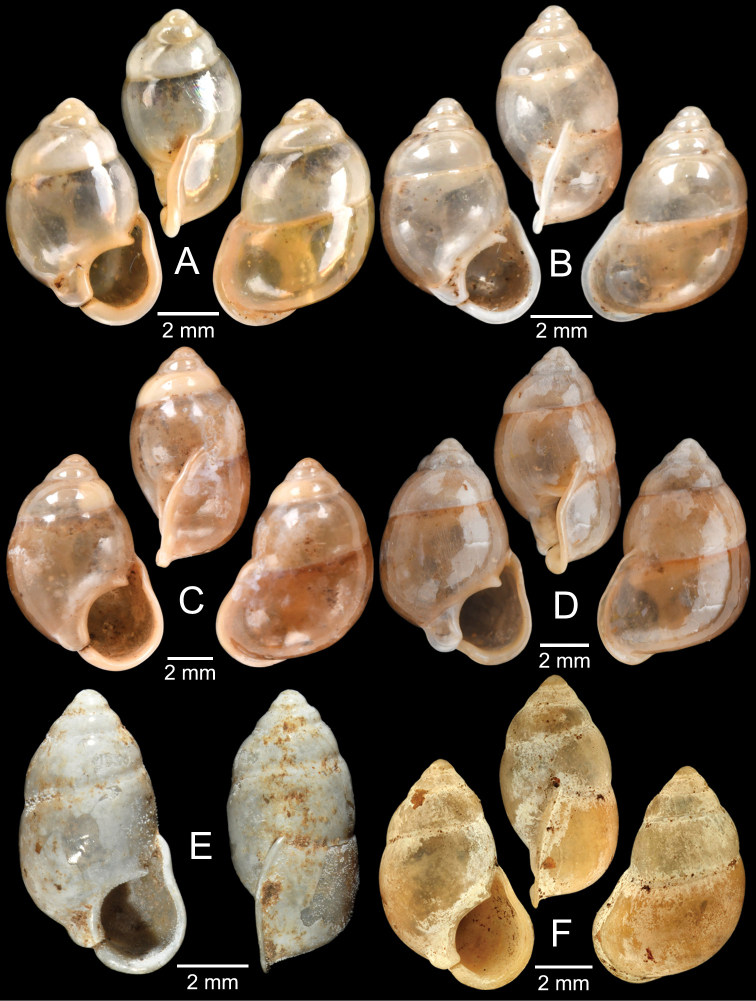
**A–D***Pupinapeguensis*: specimens **A**CUMZ 12050 from Chai Thong Wararam Temple, Nakhon Sawan **B**CUMZ 12105 from Thep Phithak Punnaram Temple, Nakhon Ratchasima **C**CUMZ 12108 from Tham Thep Bandan Temple, Phetchabun, and **D**CUMZ 12094 from Khao Tham Phra Temple, Chiang Rai **E, F***Pupinacrosseana***E** lectotype MNHN-IM-2000-35834 from Cambodge and **F** paralectotype RBINS MT966/10591 from Phnom-Rohan, Cambodge. Photo: P. Maestrati, MNHN (**E**), F. Trus, RBINS (**F**).

Figs 18B , 25F, G , 26E, F , 28A–G , 30A–D

Pupina peguensis Benson, 1860: 192, 193. Type locality: Pegu [Bago Region, Myanmar]. Nevill 1878: 300, Shuay-Gheen, Burma [Shwegyin, Bago Region, Myanmar]; Zwagabin [Zwekabin Taung mountain, Hpa-An District, Kayin State, Myanmar]. Tripathy and Sajan 2019: 508, fig. 1.
Pupina blanfordi Theobald, 1864: 247, 248. Type locality: Pegu. Hanley and Theobald 1870: 4, pl. 7, fig. 6. Reeve 1878: Pupinidae, pl. 1, sp. 6. Godwin-Austen 1897: 41, pl. 69, fig. 2, 2a, b. Syn. nov.
Pupina (Tylotoechus) blanfordi —Kobelt 1902: 309, 310. Gude 1921: 194.
Pupina (Tylotoechus) peguensis —Kobelt 1902: 319. Gude 1921: 197.
Pupina mouhoti [non Pfeiffer]—BEDO 2017: 91. Sutcharit et al. 2018: figs 4–2–7, 5–13h. Inkhavilay et al. 2019: 46, fig. 15g, Ngoy Town, Ngoy District, Luang Phrabang Province, Laos.

###### Type material examined.

***Holotype*** of *Pupinapeguensis*NZSI M.32940/9 from ‘Shuay-Gheen’, Burma figured in Tripathy and Sajan (2019: fig. 1). ***Syntype*** of *Pupinablanfordi*NHMUK 1888.12.4.100 (1 shell; Figs 26E, 28A) from Pegu.

###### Other material examined.

Specimen NHMUK ex. Cuming coll. (1 shell; labelled as *Pupinamouhoti*, Pfeiffer; Figs 26F, 28E) from Lao Mountains, Camboja. CUMZ 12050 (78 shells and 73 specimens in ethanol; Figs 25F, 28C, 30A) from Chai Thong Wararam Temple, Tak Fa District, Nakhon Sawan Province, 9 June 2017. CUMZ 12051 (170 shells and 125 specimens in ethanol; Fig. 25G) from Tham Saeng Wiset Bureau of Monks, Tak Fa District, Nakhon Sawan Province, 6 Dec. 2020. CUMZ 12105 (2 shells; Figs 28B, 30B) from Thep Phithak Punnaram Temple, Pak Chong District, Nakhon Ratchasima Province, 18 Sept. 2017. CUMZ 12107 (1 specimen in ethanol) from Tham Wua Daeng Temple, Phakdi Chumphon District, Chaiyaphum Province, 3 Sept. 2020. CUMZ 12108 (8 shells; Figs 28G, 30C) from Tham Thep Bandan Temple, Wichian Buri District, Phetchabun Province, 21 Oct. 2007. CUMZ 12109 (1 shell) from Tham Pha Ta Phon, Noen Maprang District, Phitsanulok Province, 3 Aug. 2020. CUMZ 12110 (1 shell) from Tham Wang Na Bureau of Monks, Noen Maprang District, Phitsanulok Province, 8 June 2017. CUMZ 12172 (15 shells) from Tham Pet Tham Thong Forest Park, Takhli District, Nakhon Sawan Province, 1 Dec. 2009. CUMZ 12094 (12 shells; Figs 18B, 28F, 30D) from Khao Tham Phra Temple, Mueang Chiang Rai District, Chiang Rai Province, 9 Jan. 2008. CUMZ 12095 (1 shell) from Tham Phajarui Temple, Pa Daet District, Chiang Rai Province, 25 Oct. 2008. CUMZ 12096 (9 shells) from Luang Cave, Mae Sai District, Chiang Rai Province, 23 Oct. 2015. CUMZ 12097 (3 shells) from Tham Phra Bamphen Bun Temple, Phan District, Chiang Rai Province, 29 Nov. 2009. CUMZ 12098 (1 shell) from Mae Lana checkpoint, Pang Mapha District, Mae Hong Son Province, 6 Oct. 2017. CUMZ 12099 (5 shells) from Mae Lana junction, Pang Mapha District, Mae Hong Son Province, 18 Jan. 2015. CUMZ 12100 (6 shells) from Pha Daeng Cave, Mueang Mae Hong Son District, Mae Hong Son Province, 18 Jan. 2015. CUMZ 12101 (1 specimen in ethanol) from Pha Daeng Cave, Mueang Mae Hong Son District, Mae Hong Son Province, 5 Oct. 2017. CUMZ 12102 (6 shells) from Pha Daeng Cave, Mueang Mae Hong Son District, Mae Hong Son Province, 3 Dec. 2020. CUMZ 12187 (2 shells) from Doi Ang Khang, Fang District, Chiang Mai Province, 24 Oct. 2015. CUMZ 12103 (3 specimens in ethanol; Fig. 28D) from Pha Tub Cave, Mueang Nan District, Nan Province, 11 Oct. 2009. CUMZ 12104 (24 shells) from Tham Pha Nang Khoi Temple, Rong Kwang District, Phrae Province, Thailand, 9 Oct. 2007.

###### Diagnosis.

Shell globose to ovate-fusiform; last whorl ca. 75–80% of shell height. Apertural lip thickened but not expanded; apertural lip curved when observed from lateral view. Columellar tooth fin-shaped or curved downward like an earlobe.

###### Differential diagnosis.

*Pupinapeguensis* is similar to *P.arula* in shell shape and a curved apertural lip when observed from lateral view, but differs in having a glossy shell surface. This species is also similar to *P.exclamationis* in having a glossy surface and a curved apertural lip when observed from lateral view, but differs in having a more ovate shell shape and a more distinct parietal callus.

###### Distribution.

Myanmar (Benson 1860; Theobald 1864), Luang Phrabang Province, Laos (Inkhavilay et al. 2019), northern, northeastern, and central Thailand.

###### Remarks.

Given that the holotype of *P.peguensis* and the syntype of *P.blanfordi* are highly similar in shell shape and size, and their type localities belong to the same area, *P.blanfordi* is regarded herein as a junior subjective synonym of *P.peguensis*. This species was previously identified as *P.mouhoti* (BEDO 2017; Sutcharit et al. 2018). However, compared to the type specimens of *P.mouhoti*, *P.peguensis* has a longer and wider posterior canal. In addition, the apertural lip when observed from lateral view of *P.peguensis* is curved and its columellar tooth is curved downward like an earlobe.

All specimens in the *Pupinaarula* species group from Thailand with an ovate shell shape and a curved apertural lip when observed from lateral view are herein identified as *P.peguensis* (Fig. 28A–G). However, these specimens exhibit a variable shell size (smaller with shell height 6.1 mm, shell width 4.6 mm; Fig. 28A, to larger with shell height 9.6 mm; shell width 7.1 mm; Fig. 28G). The shell shape is also variable from globose as in the type material (see Tripathy and Sajan 2019: fig. 1), to more ovate and ovate-fusiform. As this species is also similar to *P.exclamationis*, more sampling, with both morphometric and molecular phylogenetic analyses, are needed to resolve the relationship between these two species and reveal the extent of genetic differentiation or cryptic diversity within the *P.peguensis* morphotype.

##### ﻿ Pupina crosseana

Taxon classificationAnimaliaArchitaenioglossaPupinidae

Morlet, 1883

7738A796-69C2-5FE5-AA33-47EC9BC7B126

**Figure 31.**
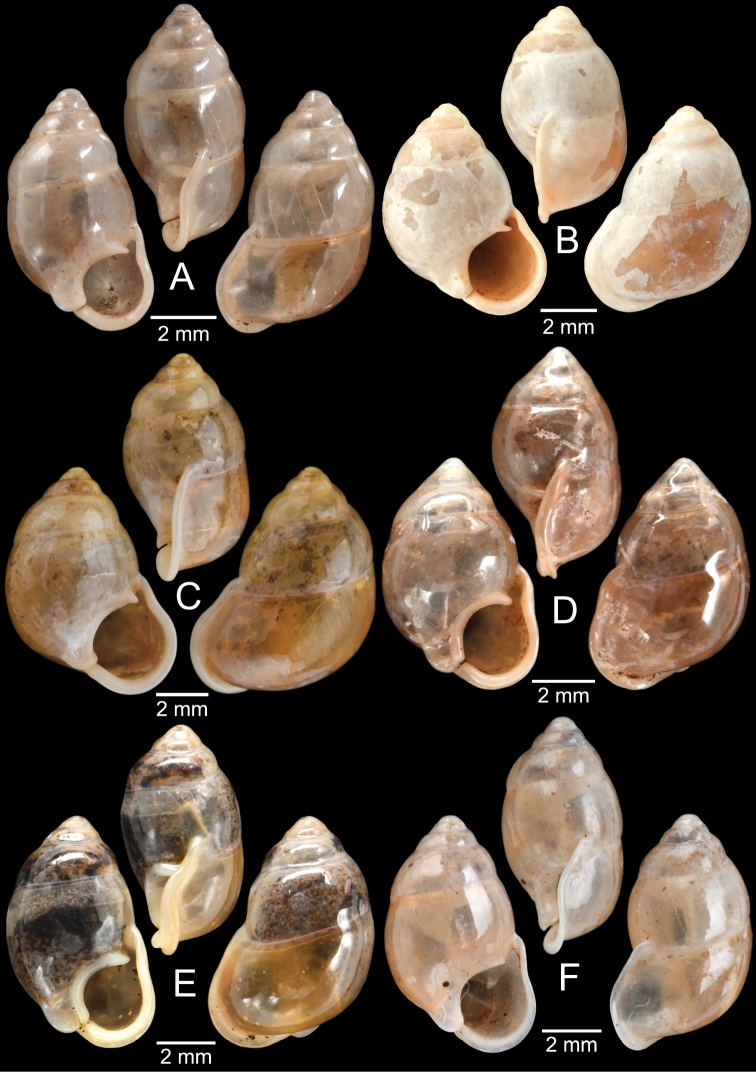
**A***Pupinacrosseana*, specimen CUMZ 12049 from Khao Jedee Temple, Nakhon Sawan **B, C***Pupinasiamensis*: **B** lectotype SMF 109948 and **C** specimen CUMZ 12052 from Sri Thong Cave, Sra Keo **D–F***Pupinabilabiata* sp. nov. **D** holotype CUMZ 12073/1, and specimens **E**CUMZ 12079 from Tham Pha Ya Temple, Loei and **F**CUMZ 12081 from Pha Jor Cave, Nong Bua Lam Phu.

Figs 28H–J , 30E, F , 31A

Pupina crosseana Morlet, 1883: 108, 109, pl. 4, fig. 5. Type locality: Cambodge [Cambodia]. Morlet 1889: 152, Pnom-Rohan (Cambodge) [Phnum Roung, Kampong Thom Province, Cambodia]; Ajuthia (Siam) [Phra Nakhon Si Ayutthaya Province, Thailand]. Fischer 1891: 107. Morlet 1904: 371, pl. 20, fig. 14, 14a. Fischer and Dautzenberg 1904: 431. Fischer-Piette 1950: 153. Fischer 1973: 48. BEDO 2017: 89.
Pupina (Tylotoechus) crosseana —Kobelt 1902: 310, 311. Hemmen and Hemmen 2001: 39.

###### Type material examined.

***Lectotype***MNHN-IM-2000-35834 (Figs 28H, 30E) from Cambodge. Paralectotype RBINS MT966/10591 (1 shell; Figs 28I, 30F) from Phnom-Rohan, Cambodge.

###### Other material examined.

CUMZ 12049 (16 shells; Figs 28J, 31A) from Khao Jedee Temple, Ta Kli District, Nakhon Sawan Province, 25 Oct. 2005.

###### Diagnosis.

Shell fusiform; last whorl ca. three quarters of shell height. Apertural lip somewhat thickened, but not expanded; apertural lip when observed from lateral view somewhat curved. Columellar tooth fin-shaped.

###### Differential diagnosis.

*Pupinacrosseana* is most similar to *P.perakensis* in having a fusiform shell shape, but differs in having the parietal callus and parietal tooth less thickened, a less curved apertural lip when observed from lateral view, and a fin-shaped columellar tooth.

###### Distribution.

Cambodia and central Thailand (Fischer and Dautzenberg 1904).

###### Remarks.

As the original description did not explicitly state that the description of this species was based on a single specimen (nor could this be inferred), the designation of a holotype by Fischer-Piette (1950) in fact constitutes a lectotype designation (ICZN 1999: Art. 74.6).

##### ﻿ Pupina siamensis

Taxon classificationAnimaliaArchitaenioglossaPupinidae

Möllendorff, 1902

59C3770E-FAE5-5DAD-BBC2-115A71AABB2B

**Figure 32.**
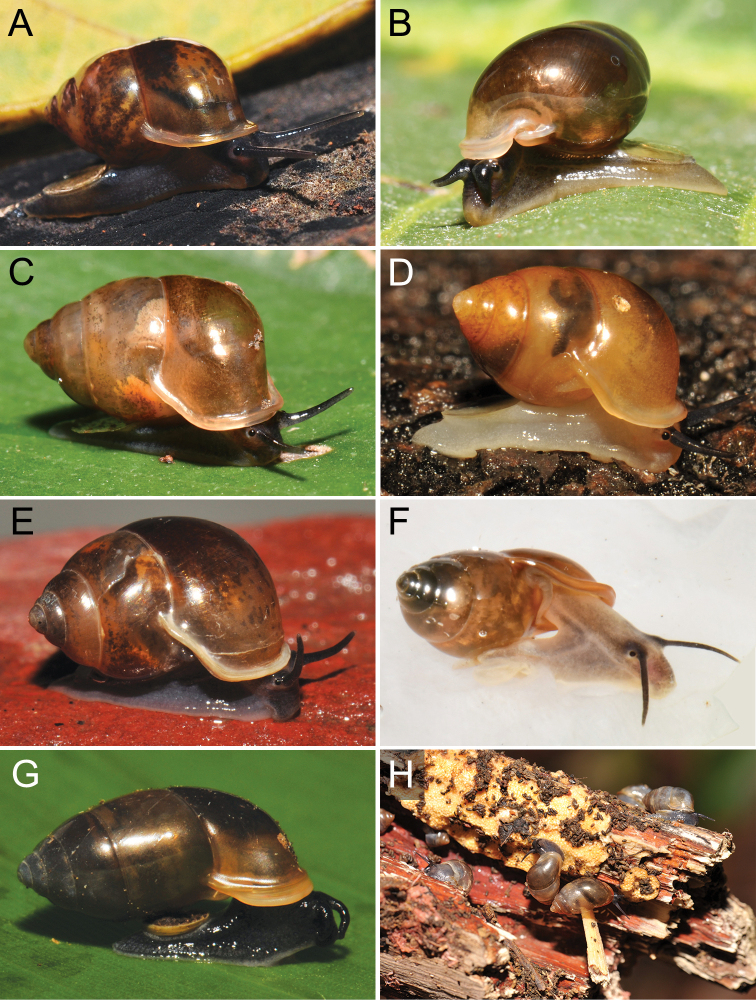
Live specimens of **A***Pupinasiamensis*, specimen CUMZ 12071 from Tham Khao Loi Temple, Rayong **B–D***Pupinabilabiata* sp. nov. **B** paratype CUMZ 12073/2 from Banpot Pisai Temple, Chumphon and specimens **C**CUMZ 12082 from Pha Jor Cave, Nong Bua Lam Phu and **D**CUMZ 12087 from Ban Yai, Surat Thani **E, F***Pupinagodwinausteni* sp. nov.: paratypes **E**CUMZ 12090/26 and **F**CUMZ 12091 from Khao Wong Cave, Uthai Thani **G, H***Pupinaaureola*: specimens **G**CUMZ 12117 from Lod Cave, Nakhon Sri Thammarat and **H**CUMZ 12121 from Tham Thong Panara Temple, Nakhon Sri Thammarat, showing its microhabitat in rotten log. All not to scale.

Figs 18C , 19B , 25H , 28K, L , 31B, C , 32A

Pupina (Tylotoechus) siamensis Möllendorff, 1902b: 160. Type locality: “Bangkok” [see Remarks]. Zilch 1957: 47, pl. 2, fig. 15. Hemmen and Hemmen 2001: 39.
Pupina siamensis —Fischer and Dautzenberg 1904: 432, Muok-Lek, Siam [Muak Lek District, Saraburi Province, Thailand]. Boonngam et al. 2008: 258, Chonburi Province, Thailand. Chanyapate et al. 2008: 2116, with text fig., Sakaerat Biosphere Reserves, Nakhon Ratchasima Province. Chidchua and Dumrongrojwattana 2010: 164, fig. 2, Klaeng District, Rayong Province and Kaenghangmaew District, Chanthaburi Province. Dumrongrojwattana 2016: 17, 18, fig. 4–4, Kaeng Hin Poeng, Thap Lan National Park, Prachin Buri Province. BEDO 2017: 94. Sutcharit et al. 2018: fig. 5–13j.

###### Type material examined.

***Lectotype***SMF 109948 (Figs 28K, 31B) from “Bangkok”, Thailand.

###### Other material examined.

CUMZ 12052 (15 shells; Figs 18C, 28L, 31C) from Sri Thong Cave, Klong Had District, Sra Keo Province, 25 Nov. 2006. CUMZ 12053 (3 shells) from Liam Cave, Klong Had District, Sra Keo Province, 25 Nov. 2006. CUMZ 12054 (7 shells and 9 specimens in ethanol) from Khao Pha Pheung Temple, Klong Had District, Sra Keo Province, 21 May 2012. CUMZ 12055 (12 shells and 1 specimen in ethanol) from Tham Khao Maka Temple, Mueang Sa Kaeo District, Sa Kaeo Province, 2 Nov. 2008. CUMZ 12056 (9 shells) from Tham Khao Chakan Temple, Khao Chakan District, Sa Kaeo Province, 7 Apr. 2000. CUMZ 12057 (9 specimens in ethanol) from Tham Khao Chakan Temple, Khao Chakan District, Sa Kaeo Province, 25 July 2018. CUMZ 12058 (3 specimens in ethanol) from Khao Chakan, Khao Chakan District, Sa Kaeo Province, 22 May 2012. CUMZ 12059 (1 shell) from Makok Waterfall, Khlung District, Chanthaburi Province, 10 Aug. 2014. CUMZ 12060 (2 specimens in ethanol) from Phlio Waterfall, Mueang Chanthaburi District, Chanthaburi Province, 20 Oct. 2010. CUMZ 12061 (3 specimens in ethanol) from Khao Sukim Temple, Tha Mai District, Chanthaburi Province, 9 Aug. 2011. CUMZ 12062 (2 specimens in ethanol) from Tham Krong Thip Bureau of Monks, Tha Mai District, Chanthaburi Province, 24 July 2018. CUMZ 12063 (2 specimens in ethanol) from Tham Khao Wong Temple, Kaeng Hang Maeo District, Chanthaburi Province, 4 Aug. 2016. CUMZ 12064 (8 shells) from Tham Khao Charoensuk Temple, Phanom Sarakham District, Chachoengsao Province, 2 Jan. 2008. CUMZ 12065 (18 shells and 3 specimens in ethanol) from Tham Khao Cha Ang On Temple, Bo Thong District, Chonburi Province, 17 Aug. 2006. CUMZ 12066 (5 shells) from Bo Thong District, Chonburi Province, 9 May 2008. CUMZ 12194 (2 specimens in ethanol) from Khao Ha Yot Temple, Bo Thong District, Chonburi Province, 6 Feb. 2022. CUMZ 12067 (2 shells and 20 specimens in ethanol; Fig. 19B) from Phromawat Temple, Si Racha District, Chonburi Province, 19 Sept. 2020. CUMZ 12068 (10 specimens in ethanol) from Pa Lilaiyawan Temple, Si Racha District, Chonburi Province, 19 Sept. 2020. CUMZ 12069 (1 shell and 9 specimens in ethanol; Fig. 25H) from Khao Chi Chan Buddha Image, Sattahip District, Chonburi Province, 19 Sept. 2020. CUMZ 12070 (1 shell and 8 specimens in ethanol) from Ban Klong Wan Pen, Sattahip District, Chonburi Province, 19 Sept. 2020. CUMZ 12071 (10 specimens in ethanol; Fig. 32A) from Tham Khao Loi Temple, Khao Chamao District, Rayong Province, 5 Sept. 2008. CUMZ 12072 (1 specimen in ethanol) from Khao Hin Tang Bureau of Monks, Klaeng District, Rayong Province, 9 June 2019.

###### Diagnosis.

Shell globose; last whorl ca. 80% of shell height. Apertural lip thickened, but not expanded; apertural lip when observed from lateral view almost straight. Columellar tooth fin-shaped.

###### Differential diagnosis.

*Pupinasiamensis* is most similar to *P.mouhoti* in having an almost straight apertural lip when observed from lateral view, but differs in having a more globose shell shape and a thicker, more distinct, parietal tooth.

###### Distribution.

Eastern and northeastern Thailand (Boonngam et al. 2008; Chanyapate et al. 2008; Chidchua and Dumrongrojwattana 2010; Dumrongrojwattana 2016).

###### Remarks.

As the original description did not explicitly state that the description of this species was based on a single specimen (nor could this be inferred), the designation of a holotype by Zilch (1957) in fact constitutes a lectotype designation (ICZN 1999: Art. 74.6).

The type locality of this species in Bangkok, the capital city of Thailand, as designated by von Möllendorff (1902b) is dubious. This species was described based on a collection made by the butterfly collector, H. Fruhstorfer, who made an expedition in Thailand (Lamas 2005). The type locality “Bangkok” is probably not the location where the type specimen was collected. The probable type locality would be “Muok-Lek” [Muak Lek District, Saraburi Province, Thailand] as indicated in Fischer and Dautzenberg (1904), and several butterfly specimens were also collected from this site by H. Fruhstorfer. This locality is in the same vicinity as recent records and our collecting localities of *P.siamensis*.

##### ﻿ Pupina bilabiata

Taxon classificationAnimaliaArchitaenioglossaPupinidae

Jirapatrasilp sp. nov.

5EC10E16-5CC2-59D3-9290-F05016543641

https://zoobank.org/E970A73C-E3EE-4DDA-B7C8-1B9D7960E3EF

**Figure 33.**
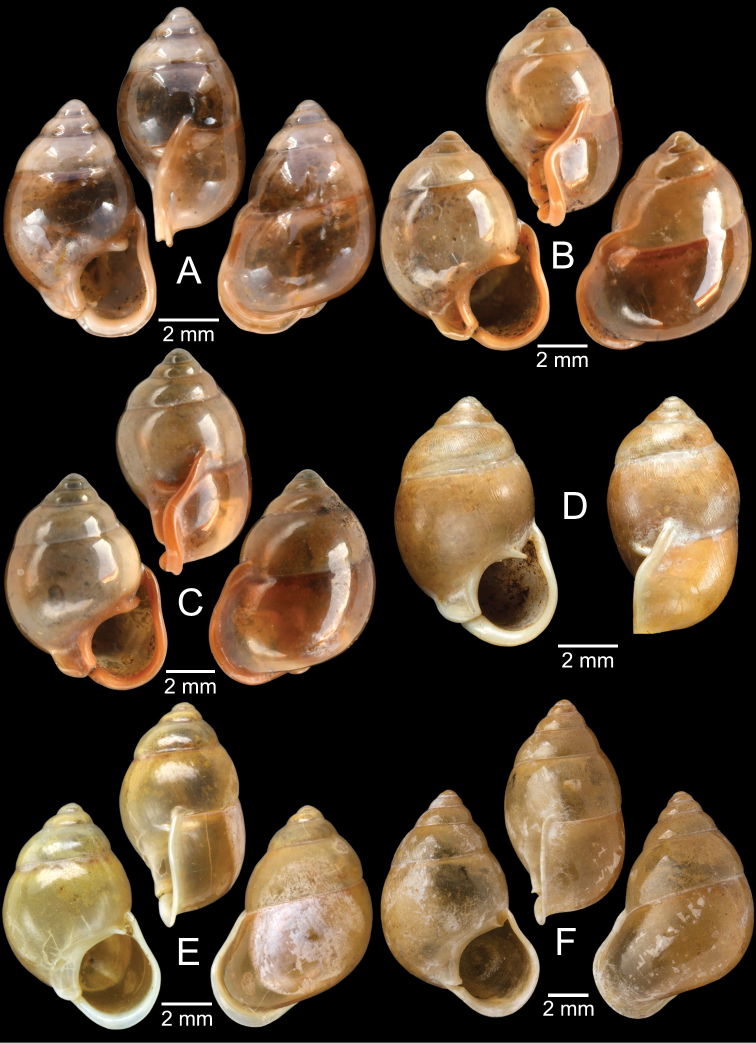
**A***Pupinabilabiata* sp. nov., specimen CUMZ 12086 from Na San Temple, Surat Thani **B, C***Pupinagodwinausteni* sp. nov. **B** holotype CUMZ 12090/1 and **C** paratype CUMZ 12090/2 from Khao Wong Cave, Uthai Thani **D***Pupinaarula*, syntype UMZC I.103025 “Ind” **E***Pupinamouhoti*, possible syntype NHMUK ex. Cuming coll. from Camboja **F***Pupinavescoi*, syntype NHMUK 1893.2.4.767 from Cochin China. Photo: J. Ablett, H. Taylor, NHM (**D**).

Figs 28M–P , 31D–F , 32B–D , 33A

###### Type material examined.

***Holotype***CUMZ 12073/1 (Figs 28M, 31D), 31 July 2019, coll. C. Sutcharit, A. Pholyotha. Measurement: shell height 7.4 mm, shell width 5.0 mm and 5½ whorls. ***Paratypes***CUMZ 12073/2–13 (12 specimens in ethanol; Fig. 32B) and NHMUK 20210334 (2 shells), same data as holotype.

###### Type locality.

Banpot Pisai Temple, Lang Suan District, Chumphon Province, Thailand, 9°56'05.0"N, 99°08'56.7"E, 20 m amsl.

###### Other material examined.

CUMZ 12074 (11 shells) from Bat Cave, Phu Pha Man District, Khon Kaen Province, 20 Oct. 2007. CUMZ 12075 (9 shells and 7 specimens in ethanol) from Phraya Nakharaj Cave, Phu Pha Man District, Khon Kaen Province, 21 July 2020. CUMZ 12076 (4 shells) from Tham Pha Pu Temple, Mueang Loei District, Loei Province, 28 Oct. 2018. CUMZ 12077 (1 shell and 17 specimens in ethanol) from Tham Pha Pu Temple, Mueang Loei District, Loei Province, 1 Sept. 2020. CUMZ 12078 (3 shells) from Phu Pha Lom, Mueang Loei District, Loei Province, 1 Sept. 2020. CUMZ 12079 (2 shells; Figs 28N, 31E) from Tham Pha Ya Temple, Na Duang District, Loei Province, 28 Oct. 2018. CUMZ 12080 (2 specimens in ethanol) from Hin Pha Ngam Park, Nong Hin District, Loei Province, 2 Sept. 2020. CUMZ 12081 (13 shells; Figs 28O, 31F) from Pha Jor Cave, Na Wang District, Nong Bua Lam Phu Province, 15 Oct. 2007. CUMZ 12082 (15 shells and 9 specimens in ethanol; Fig. 32C) from Pha Jor Cave, Na Wang District, Nong Bua Lam Phu Province, 31 Aug. 2020. CUMZ 12083 (2 shells) from Tham Suwannakhuha Temple, Suwannakhuha District, Nong Bua Lam Phu Province, 31 Aug. 2020. CUMZ 12084 (8 shells) from Pa Pha Ya Temple, Suwannakhuha District, Nong Bua Lam Phu Province, 31 Aug. 2020. CUMZ 12085 (2 specimens in ethanol) from Phu Thong Thep Nimit Temple, Nong Saeng District, Udon Thani Province, 30 Aug. 2020. CUMZ 12086 (2 shells; Figs 28P, 33A) from Na San Temple, Ban Na San District, Surat Thani Province, 3 July 2017. CUMZ 12087 (2 specimens in ethanol; Fig. 32D) from Ban Yai, Phanom District, Surat Thani Province, 7 Aug. 2016. CUMZ 12088 (2 shells) from Tham Nam Lod Thepnimit Bureau of Monks, Sawi District, Chumphon Province, 30 July 2019. CUMZ 12089 (1 specimen in ethanol) from Tham Kanlayanamit Temple, Tham Phannara District, Nakhon Si Thammarat Province, 4 July 2017.

###### Diagnosis.

Shell ovate-fusiform to fusiform; last whorl ca. three quarters of shell height. Apertural lip highly thickened, slightly expanded; with a furrow between inner and outer peristomes; inner peristome thickened, cord-like; apertural lip curved when observed from lateral view. Columellar tooth curved downward like an earlobe.

###### Differential diagnosis.

*Pupinabilabiata* sp. nov. is similar to *P.peguensis* in shell shape, but differs in having a furrow between inner and outer peristomes, with an inner peristome thickened and cord-like. This furrow also appears in *P.godwinausteni* sp. nov. and *P.stoliczkai* sp. nov., but *P.godwinausteni* sp. nov. is larger and more globose, and the apertural lip when observed from lateral view is more angled than that of *P.bilabiata* sp. nov., whereas *P.stoliczkai* sp. nov. belongs to the *P.aureola* species group.

###### Description.

Shell height 4.0–8.4 mm; shell width 4.4–5.7 mm. Shell ovate-fusiform to fusiform, solid, semi-transparent, whitish to pale brown, devoid of prominent sculpture on glazed smooth surface. Apex obtuse. Growth lines on shell surface inconspicuous. Whorls 5½–6, last whorl large (ca. three quarters of shell height) and bulging slightly. Spire angle ca. 80°, somewhat extended. Sutures slightly impressed, but shallow. Aperture circular; lip thickened to highly thickened (ca. 0.5–0.6 mm wide and 0.3–0.6 mm thick) with paler colour, slightly expanded; apertural lip curved when observed from lateral view. Apertural lip with a furrow between inner and outer peristomes, with inner peristome thickened and cord-like. Parietal callus sharply defined and thickened with paler colour. Peristome interrupted by two canals; posterior canal ca. 0.8–0.9 mm long, 0.5 mm at its widest, curved outward and bulging at outer margin; anterior canal slit-like, as long as apertural lip width. Parietal tooth indistinct to thick; columellar tooth curved downward like an earlobe (ca. 1.5 mm long, 0.9 mm wide and 0.5 mm thick), covering anterior canal. Umbilicus closed. Operculum round, yellowish, transparent corneous with uneven edge.

###### Etymology.

The Latin specific epithet *bilabiata* means “with double lip” representing the separation of the inner and outer peristomes by a furrow.

###### Distribution.

Northeastern and southern Thailand.

###### Remarks.

This new species has a disjunct distribution and shows varying degrees of thickness of the inner peristome within specimens from the same collecting localities.

##### ﻿ Pupina godwinausteni

Taxon classificationAnimaliaArchitaenioglossaPupinidae

Jirapatrasilp sp. nov.

27DC3A32-1413-5276-9451-22EA2D7FA9AD

https://zoobank.org/EF5C2A8A-36DE-4C06-896A-CE55F9520C60

Figs 10C , 28Q, R , 32E, F , 33B, C

###### Type material.

***Holotype***CUMZ 12090/1 (Figs 10C, 28Q, 33B), 5 June 2017, coll. C. Sutcharit, R. Srisonchai, A. Pholyotha. Measurement: shell height 8.8 mm, shell width 6.8 mm and 5 whorls. ***Paratypes***CUMZ 12090/2‒26 (24 shells and 1 specimen in ethanol; Figs 28R, 32E, 33C) and NHMUK 20210335 (3 shells), same data as holotype; CUMZ 12091 (20 shells and 24 specimens in ethanol; Fig. 32F) from the type locality, 5 Dec. 2020, coll. P. Jirapatrasilp, C. Sutcharit, A. Pholyotha.

###### Type locality.

Khao Wong Cave, Ban Rai District, Uthai Thani Province, Thailand, 15°01'52.6"N, 99°27'23.3"E, 246 m amsl.

###### Other material examined.

CUMZ 12092 (2 shells) from Tham Namthip Bureau of Monks, Lan Sak District, Uthai Thani Province, 28 July 2016. CUMZ 12093 (1 specimen in ethanol) from Hup Pa Tat, Lan Sak District, Uthai Thani Province, 1 Oct. 2018.

###### Diagnosis.

Shell globose; last whorl ca. 80% of shell height. Apertural lip very thickened and slightly expanded; with a furrow between inner and outer peristomes; inner peristome thickened, cord-like; apertural lip angled when observed from lateral view. Columellar tooth curved downward like an earlobe.

###### Differential diagnosis.

The globose shell shape of *P.godwinausteni* sp. nov. is most similar to *P.siamensis*, but *P.godwinausteni* sp. nov. differs from *P.siamensis* in having a larger shell, a more prominent parietal callus, a thicker apertural lip with a furrow between inner and outer peristomes, with an inner peristome thickened and cord-like, a longer posterior canal, a wider and more curved columellar tooth, and a more angled apertural lip when observed from lateral view.

###### Description.

Shell height 7.7–9.5 mm; shell width 5.5–7.0 mm. Shell globose, solid, semi-transparent, brown, devoid of prominent sculpture on glazed smooth surface. Apex obtuse. Growth lines on shell surface inconspicuous. Whorls 5, last whorl large (ca. 80% of shell height) and bulging. Spire angle ca. 90°, somewhat extended. Sutures slightly impressed, but shallow. Aperture circular; lip highly thickened (ca. 0.4–0.5 mm wide and 0.5–0.6 mm thick) with darker colour, slightly expanded; apertural lip when observed from lateral view angled. Apertural lip with a furrow between inner and outer peristomes, with inner peristome thickened and cord-like. Parietal callus thickened with darker colour. Peristome interrupted by two canals; posterior canal ca. 1.0–1.2 mm long, 0.6 mm at its widest, curved outward and bulging at the outer margin; anterior canal slit-like, as long as apertural lip width. Parietal tooth thick; columellar tooth curved downward like an earlobe (ca. 2.2 mm long, 1.2 mm wide and 0.5 mm thick), covering anterior canal. Umbilicus closed. Operculum round, yellowish, and transparent corneous with uneven edge.

###### Etymology.

The specific epithet is dedicated to H.H. Godwin-Austen, a British malacologist, who prominently contributed to malacological studies in South and Southeast Asia.

###### Distribution.

This new species is found in Uthai Thani Province, Thailand.

#### Species of group II (*P.arula* species group) with uncertain record from Thailand

##### ﻿ Pupina arula

Taxon classificationAnimaliaArchitaenioglossaPupinidae

Benson, 1856

6F8DB999-B63E-5743-A58A-DE18F878CB59

Figs 29A, B , 33D

Pupina arula Benson, 1856: 230. Type locality: ad Yunglaw, in valle Tenasserim [Tanintharyi Region, Myanmar]. Theobald 1858 [1857]: 247. Pfeiffer 1860: 141, pl. 37, figs 7–9. Hanley and Theobald 1870: 4, pl. 7, fig. 4. Reeve 1878: Pupinidae, pl. 1, sp. 5. Crosse 1879: 340 (part). de Morgan 1885: 413 (part). von Möllendorff 1887 [1886]: 314 (part). Godwin-Austen 1897: 37, 38, pl. 69, fig. 1, 1a. BEDO 2017: 88. Sutcharit et al. 2018: fig. 5–13e.
Pupina avula [sic]—Sowerby I 1866: Pupinidae, pl. 3 (pl. 265), Pupina, fig. 3.
Pupina (Tylotoechus) arula —Kobelt 1902: 307. Gude 1921: 193, 194 (part). Solem 1966: 12, Doi Sutep [Doi Suthep Mountain, Chiang Mai Province, Thailand]. Hemmen and Hemmen 2001: 39.
Pupina arula arula —Maassen 2001: 40.

###### Type material examined.

***Syntype***UMZC I.103025 (1 shell; Figs 29A, 33D) from the R. McAndrew collection labelled “Bens. col., Ind”.

###### Other material examined.

Specimen NHMUK 1888.12.4.109 (1 shell; Fig. 29B) from Yunglaw, Myanmar, the W. Theobald collection.

###### Diagnosis.

Shell ovate; last whorl ca. 80% of shell height. Shell surface matt. Apertural lip thickened but not expanded; apertural lip curved when observed from lateral view. Columellar tooth fin-shaped.

###### Differential diagnosis.

*Pupinaarula* can be distinguished from all other species in the *P.arula* species group from mainland Southeast Asia by a matt shell surface.

###### Distribution.

Myanmar and an uncertain record from northern Thailand (Solem 1966).

###### Remarks.

No material of this species was found during this survey. The specimen of *P.arula* mentioned in Davison (1995: 237) from Temengor dam, Perak, Malaysia possibly belongs to *P.perakensis*.

##### ﻿ Pupina mouhoti

Taxon classificationAnimaliaArchitaenioglossaPupinidae

Pfeiffer, 1861

46C21D61-9061-5337-960C-F1F5A34F076F

Figs 29C, D , 33E

Pupina mouhoti Pfeiffer, 1861: 196. Type locality: Camboja [Cambodia]. Pfeiffer 1863b [1862]: 278, pl. 36, fig. 7. Sowerby I 1866: Pupinidae, pl. 3 (pl. 265), Pupina, fig. 16. von Martens 1867: 67, Siam (?). Reeve 1878: Pupinidae, pl. 2, sp. 13. Morlet 1889: 152, Montson Kreang [possibly refers to Phum Ang Sang Kream, Kampong Speu Province, Cambodia], Battambang [Battambang Province, Cambodia], forêt de Srakéo (Siam) [Srakeo Province, Thailand]. Fischer 1891: 108. Fischer and Dautzenberg 1904: 431, Mont Souten à l’Ouest de Xieng-Mai, Laos occidental [Chiang Mai Province, Thailand]; Luang-Prabang [Luang Prabang Province, Laos]. Saurin 1953: 113, Pa Hia, Tran Ninh Province, Laos [probably refers to Ban Namthong, Longchaeng District, Xaisomboun Province, Laos]. Fischer 1973: 48.
Pupina (Tylotoechus) mouhoti —Kobelt 1902: 317. Hemmen and Hemmen 2001: 39.

###### Type material examined.

***Possible syntypes***NHMUK ex. Cuming coll. (3 shells; Figs 29C, D, 33E) from Cambodia.

###### Diagnosis.

Shell ovate-fusiform; last whorl ca. 80% of shell height. Apertural lip slightly thickened and slightly expanded; apertural lip when observed from lateral view almost straight. Columellar tooth curved downward like an earlobe.

###### Differential diagnosis.

*Pupinamouhoti* is most similar to *P.siamensis* and *P.vescoi*, but different from *P.siamensis* by a more ovate-fusiform shell shape and a smaller parietal tooth, and differs from *P.vescoi* by a smaller shell, a shorter spire, a more distinct parietal tooth, and having a columellar tooth curved downward like an earlobe.

###### Distribution.

Cambodia, Laos, and an uncertain record from Thailand (Fischer 1891; Kobelt 1902).

###### Remarks.

No material of this species was found during this survey. The specimens from Srakeo Province mentioned in Morlet (1889) possibly belong to *P.siamensis*. In addition, some specimens mentioned in Fischer and Dautzenberg (1904) and Saurin (1953) possibly belong to *P.peguensis*.

#### Species of group II (*P.arula* species group) from other parts of mainland Southeast Asia not recorded for Thailand

##### ﻿ Pupina vescoi

Taxon classificationAnimaliaArchitaenioglossaPupinidae

Morelet, 1862

ADB05C32-1480-5A21-807E-68DEC70FED07

**Figure 34.**
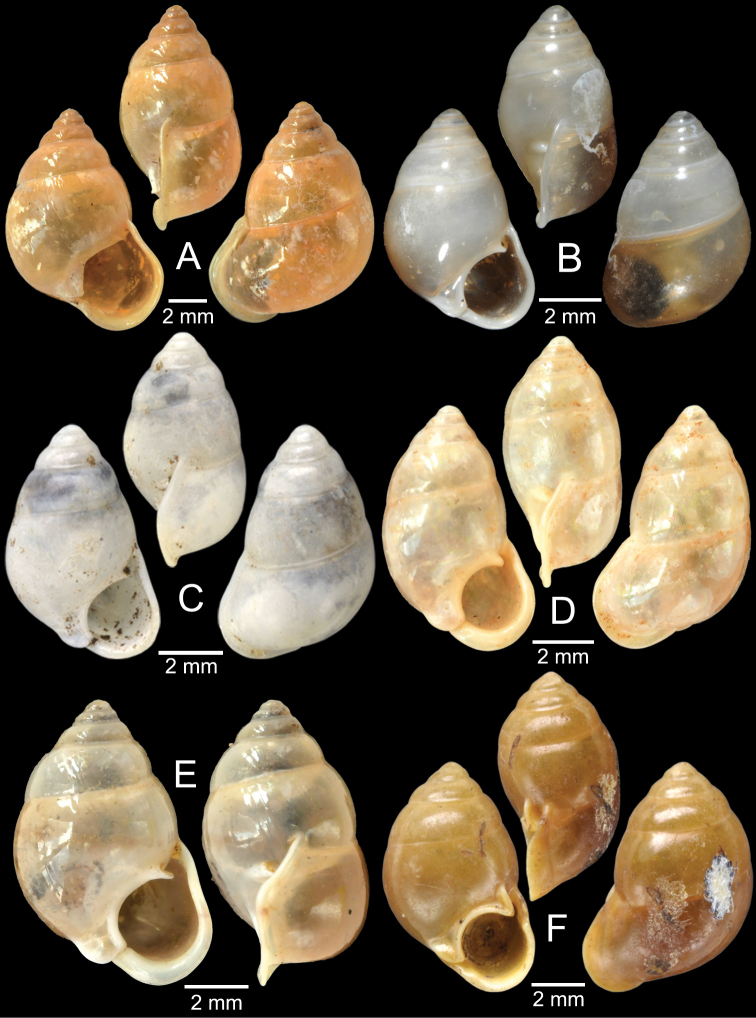
**A***Pupinavescoi*, specimen SMF 109956/1 from Cochin China **B, C***Pupinaexclamationis***B** syntype MNHN-IM-2000-35840 from Tonkin and **C** specimen NHMUK 1901.12.23.205 “forma minor” from Than-Moi, Tonkin **D***Pupinaperakensis*, lectotype SMF 109969/1 from Bukit Pondong, Perak **E***Pupinaexcisa*, lectotype SMF 110778/1 from Kelantan **F***Pupinaaureola*, possible syntype NHMUK 1988.12.4.101 from Pinang. Photo: P. Maestrati, MNHN (**B**).

Figs 29E, F , 33F , 34A

Pupina vescoi Morelet, 1862: 479. Type locality: Bien-Hoa Cochinchinae [Bien Hoa, Dong Nai Province, Vietnam]. Sowerby I 1866: Pupinidae, pl. 3 (pl. 265), Pupina, fig. 26. Morelet 1875: 287, 288, pl. 13, fig. 11. Nevill 1878: 299. Reeve 1878: Pupinidae, pl. 2, sp. 18. Fischer 1891: 107, Environs de Saigon [Ho Chi Minh City, Vietnam]; Fuyen-Moth [Phu Yen Province, Vietnam]. Fischer and Dautzenberg 1904: 432, Thudaumot [Thu Dau Mot, Binh Duong Province, Vietnam]. Raheem et al. 2017: 5 (plate figure).
Pupina (Tylotoechus) vescoi —Kobelt 1902: 325, Pulo-Condor [Con Dao Island, Ba Ria-Vung Tau Province, Vietnam].

###### Type material examined.

***Syntypes***NHMUK 1893.2.4.767–769 (3 shells; Figs 29E, 33F) from Cochin China.

###### Other material examined.

SMF 109956/1 (1 shell; Figs 29F, 34A) from Cochin China.

###### Diagnosis.

Shell ovate-fusiform; last whorl ca. three quarters of shell height. Apertural lip slightly thickened and slightly expanded; apertural lip when observed from lateral view almost straight. Parietal tooth small, indistinct; columellar tooth fin-shaped, not covering slit-like anterior canal.

###### Differential diagnosis.

*Pupinavescoi* is most similar to *P.mouhoti* and *P.siamensis*, but differs in having a larger shell with a higher spire, a smaller, indistinct parietal tooth, and a fin-shaped columellar tooth not covering a slit-like anterior canal.

###### Distribution.

South Vietnam (Fischer and Dautzenberg 1904).

##### ﻿ Pupina exclamationis

Taxon classificationAnimaliaArchitaenioglossaPupinidae

Mabille, 1887

06777E90-C84D-5B85-8F5A-A98785A5D09A

Figs 29I–K , 34B, C

Pupina exclamationis Mabille, 1887: 137, 138, pl. 4, figs 11, 12. Type locality: Tonkin. Fischer 1891: 108. Fischer and Dautzenberg 1904: 431, Bac-Kan, Tonkin; Monts Mauson, Tonkin [Mount Mau Son, Lang Son Province, Vietnam]. Do et al. 2015: 126, fig. 6a, Son La Province, Vietnam.
Pupina (Tylotoechus) exclamationis —Kobelt 1902: 312.

###### Type material examined.

***Syntypes***MNHN-IM-2000-35840 (4 shells; Figs 29I, J, 34B) from Tonkin.

###### Other material examined.

NHMUK 1901.12.23.205‒210 “forma minor” ex. H. Fruhstorfer coll. (5 shells; Figs 29K, 34C) from Than-Moi, Tonkin.

###### Diagnosis.

Shell ovate-fusiform to fusiform; last whorl ca. three quarters of shell height. Apertural lip somewhat thickened but not expanded; apertural lip slightly curved when observed from lateral view. Columellar tooth fin-shaped.

###### Differential diagnosis.

*Pupinaexclamationis* is most similar to *P.peguensis* in having a glossy surface and a curved apertural lip when observed from lateral view, but differs in having a more fusiform shell shape and a less distinct parietal callus.

###### Distribution.

Northern Vietnam (Do et al. 2015).

##### ﻿ Pupina perakensis

Taxon classificationAnimaliaArchitaenioglossaPupinidae

Möllendorff, 1891

1866D4BC-9445-5695-8641-4C0FFD34B9B1

Figs 29G , 34D

Pupina arula var. perakensis Möllendorff, 1891: 345. Type locality: Bukit Pondong, Perak [Gunung Pondok, Perak State, Malaysia].
Pupina arula perakensis — van Benthem Jutting 1949: 58, Cameron Highlands, Pahang; Telom Valley, near Gunong Siku, Pahang; Kuala Legap, Plus Valley, Perak [Malaysia]. van Benthem Jutting 1960: 13, hill near the hot springs, ca. 400 m from the main road from Tandjong Rambutan to Ipoh, near Tambun, Perak. Maassen 2001: 40.
Pupina (Tylotoechus) arula perakensis —Laidlaw 1928: 34. Zilch 1957: 44, pl. 2, fig. 17.
Pupina lowi [non Morgan]—Foon et al. 2017: 40, 41, fig. 15d, Ipoh, Perak.
Pupina tchehelensis [non Morgan]—Foon et al. 2017: 41, fig. 16a, Ipoh, Perak.

###### Type material examined.

***Lectotype***SMF 109969/1 (Figs 29G, 34D) from Bukit Pondong, Perak.

###### Diagnosis.

Shell fusiform; last whorl ca. 70% of shell height. Apertural lip thickened but not expanded; apertural lip curved when observed from lateral view. Parietal callus and parietal tooth highly thickened; columellar tooth curved downward like an earlobe.

###### Differential diagnosis.

*Pupinaperakensis* is most similar to *P.crosseana*, but differs in parietal callus and parietal tooth very thickened, and a columellar tooth curved downward like an earlobe.

###### Distribution.

Perak and Pahang States, Malaysia (Maassen 2001).

###### Remarks.

This taxon has always been treated as a subspecies of *P.arula* (van Benthem Jutting 1949; Zilch 1957; Maassen 2001). However, it is different from *P.arula* in having a glossy shell surface, a more fusiform shape with a higher spire; and a less bulging last whorl; additionally, the occurrence of this taxon is ca. 1,800 km from that of *P.arula*. Thus, this taxon is herein elevated to the specific level.

By comparing with the type specimen, the specimen of *P.tchehelensis* figured in Foon et al. (2017: fig. 16a) from Gunung Tempurung Plot 2, Ipoh, Perak should belong to *P.perakensis* (Foon, pers. comm.). Although the *P.lowi* specimen figured in Foon et al. (2017: 15d) from Bat Cave Hill, Ipoh, Perak has a shorter spire, we preliminarily identify this specimen as *P.perakensis* as well due to an overall character in the *Pupinaarula* species group, a similar glossy surface to the type specimen, and its nearby locality to the type locality.

##### ﻿ Pupina excisa

Taxon classificationAnimaliaArchitaenioglossaPupinidae

Möllendorff, 1902

8900D545-59A1-5D06-8D7C-13B8E99736A2

Figs 29H , 34E

Pupina (Tylotoechus) excisa Möllendorff, 1902a: 143. Type locality: Kelantan [Malaysia]. Laidlaw 1928: 34. Zilch 1957: 45, pl. 2, fig. 18.
Pupina excisa —Chan 1998a: 4, Ipoh, Perak. Chan 1998b: 2. Maassen 2001: 41. BEDO 2017: 90.

###### Type material examined.

***Lectotype***SMF 110778/1 (Figs 29H, 34E) from Kelantan.

###### Diagnosis.

Shell ovate with higher spire; last whorl ca. three quarters of shell height. Apertural lip somewhat thickened but not expanded; apertural lip when observed from lateral view angled. Columellar tooth curved downward like an earlobe.

###### Differential diagnosis.

*Pupinaexcisa* can be distinguished from all other species in the *P.arula* species group from mainland Southeast Asia by an ovate shell shape with a higher spire, and an angled apertural lip when observed from lateral view. *Pupinaexcisa* is different from *P.mouhoti* in having a thicker, more prominent parietal tooth.

###### Distribution.

Kelantan and Perak States, Malaysia (Maassen 2001).

#### Group III. *Pupinaaureola* species group

Figs 10D, 18D, 19C

This species group is characterised by an indistinct to thickened triangular or fin-shaped parietal tooth located next to a posterior canal. A columellar tooth is less thickened, never ear shaped and mostly fin-shaped, located next to an anterior canal. Both the anterior and posterior canals are either slit-like or widening toward the outer margin when observed from apertural view. An outer apertural lip is straight or slightly curved when observed from lateral view. An operculum is round, thick, flat to concave, multispiral, whitish to pale yellow, opaque corneous with smooth edge.

**Figure 35.**
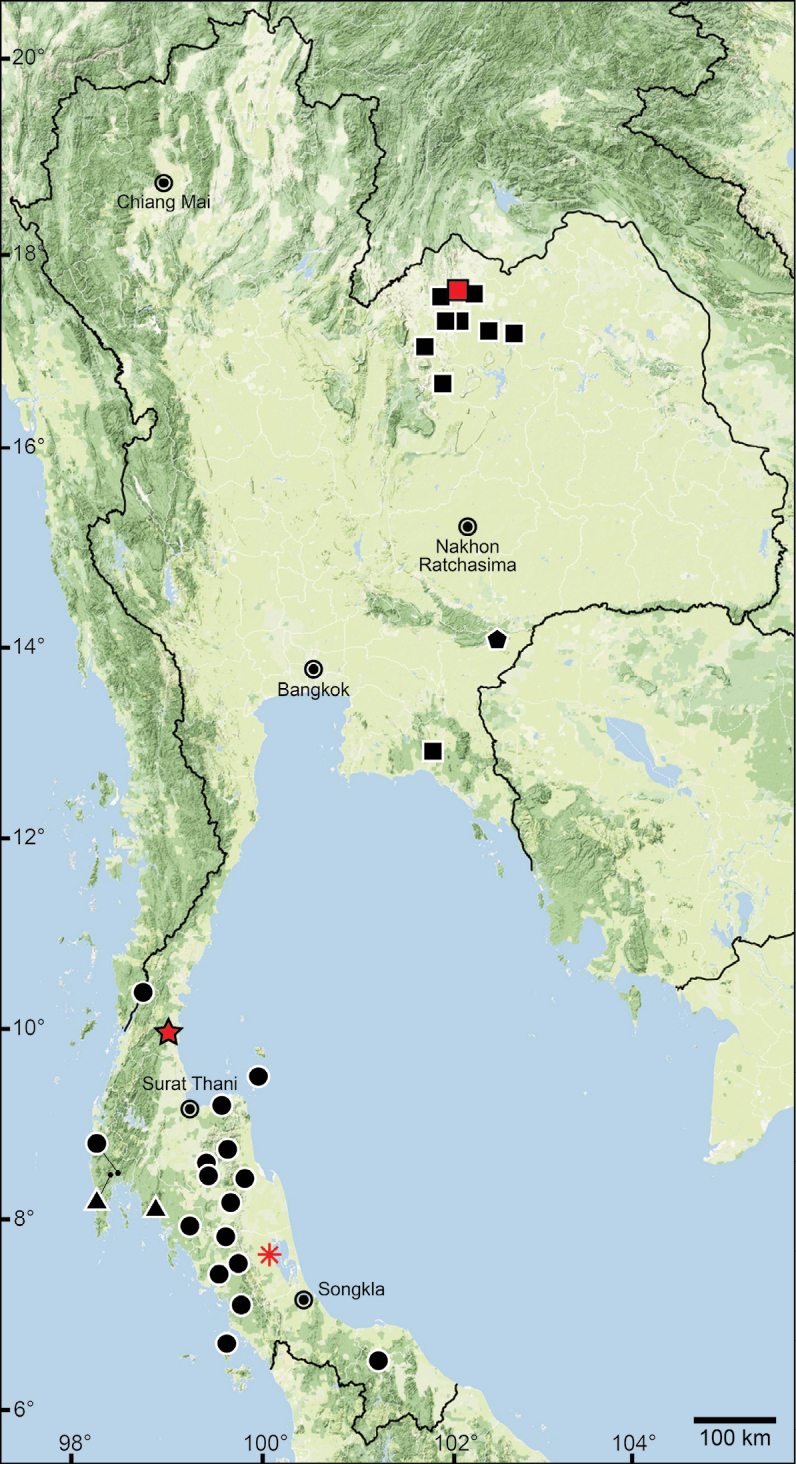
Distribution map of the *Pupinaaureola* species group: *Pupinaaureola* (circle), *Pupinapaviei* (pentagon), *Pupinatchehelensis* (triangle), *Pupinadorriisanensis* ssp. nov. (square), *Pupinalatisulci* sp. nov. (asterisk), and *Pupinastoliczkai* sp. nov. (star). Each red symbol indicates the type locality of its respective taxon.

**Figure 36.**
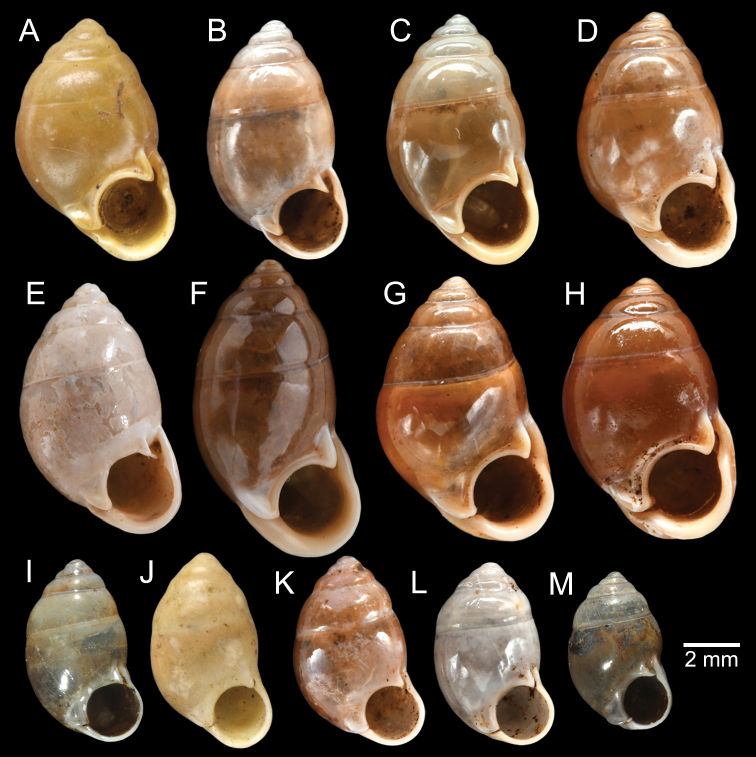
Shells of *Pupinaaureola* species group from mainland Southeast Asia **A–F***Pupinaaureola***A** possible syntype NHMUK 1988.12.4.101 and specimens **B**CUMZ 12124 **C**CUMZ 12126 **D**CUMZ 12130 **E**CUMZ 12133, and **F**CUMZ 12121 **G, H***Pupinastoliczkai* sp. nov. **G** holotype CUMZ 12147/1 and **H** paratype NHMUK 20210336 **I, J***Pupinadorridorri***I** paralectotype MNHN-IM-2000-35836 and **J** specimen NHMUK ex. A.J. Piele Colln. Acc. No. 2242 **K, L***Pupinadorriisanensis* spp. nov. **K** holotype CUMZ 12140/1 and **L** specimen CUMZ 12137 **M***Pupinabrachysoma*, lectotype MNHN-IM-2000-9652. Photo: P. Maestrati, MNHN (**I, M**)

**Figure 37.**
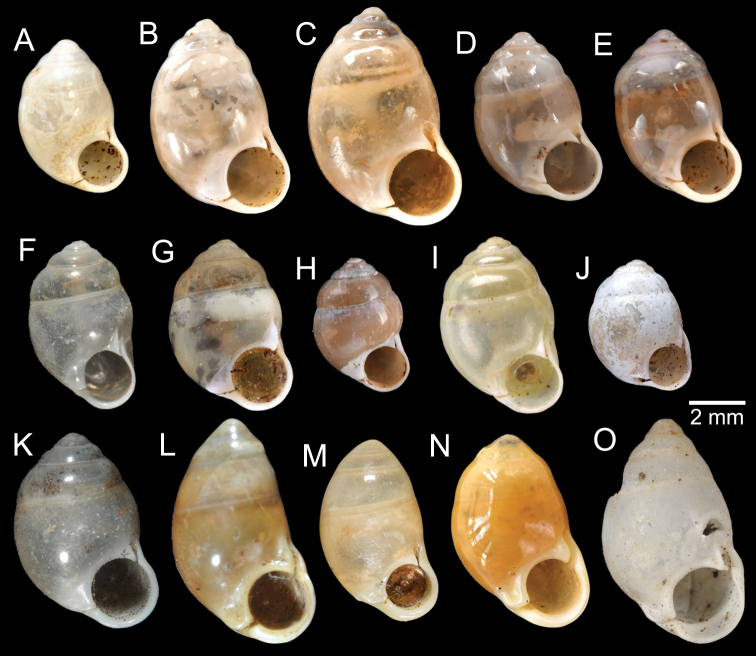
Shells of *Pupinaaureola* species group from mainland Southeast Asia. **A–C***Pupinatchehelensis*: specimens **A**SMF 109947/6 **B**CUMZ 12135, and **C**CUMZ 12136 **D, E***Pupinalatisulci* sp. nov. **D** holotype CUMZ 12146/1 and **E** paratype CUMZ 12146/2 **F–I***Pupinapaviei***F** paralectotype MNHN-IM-2000-35837 **G** paralectotype RBINS 525404, and specimens **H**CUMZ 12134 and **I**NHMUK ex. Dautzenberg coll. **J***Pupinatongupensis*, syntype NHMUK 1906.4.4.38 **K***Pupinalowi*, syntype MNHN-IM-2000-35846 **L***Pupinaanceyi*, syntype MNHN-IM-2000-35833 **M***Pupinalaffonti*, syntype MNHN-IM-2000-9656 **N***Pupinasolidula*, lectotype SMF 109915/1 **O***Pupinadouvillei*, holotype MNHN-IM-2000-35532. Photo: M. Caballer, P. Maestrati, MNHN (**F, K–O**), F. Trus, RBINS (**G**), J. Ablett, H. Taylor, NHM (**J**).

This species group from mainland Southeast Asia contains 13 species and one subspecies, including three nominal species, two new species (*P.latisulci* sp. nov. and *P.stoliczkai* sp. nov.), and one new subspecies (*P.dorriisanensis* ssp. nov.) from Thailand. The distribution of the *P.aureola* species group in Thailand is provided in Fig. 35. A synoptic view of all species within the *P.aureola* species group from mainland Southeast Asia is given in Figs 36, 37 to provide the comparative size.

##### ﻿ Pupina aureola

Taxon classificationAnimaliaArchitaenioglossaPupinidae

Stoliczka, 1872

F895E596-0025-550F-A394-CC1243A1DE43

**Figure 38.**
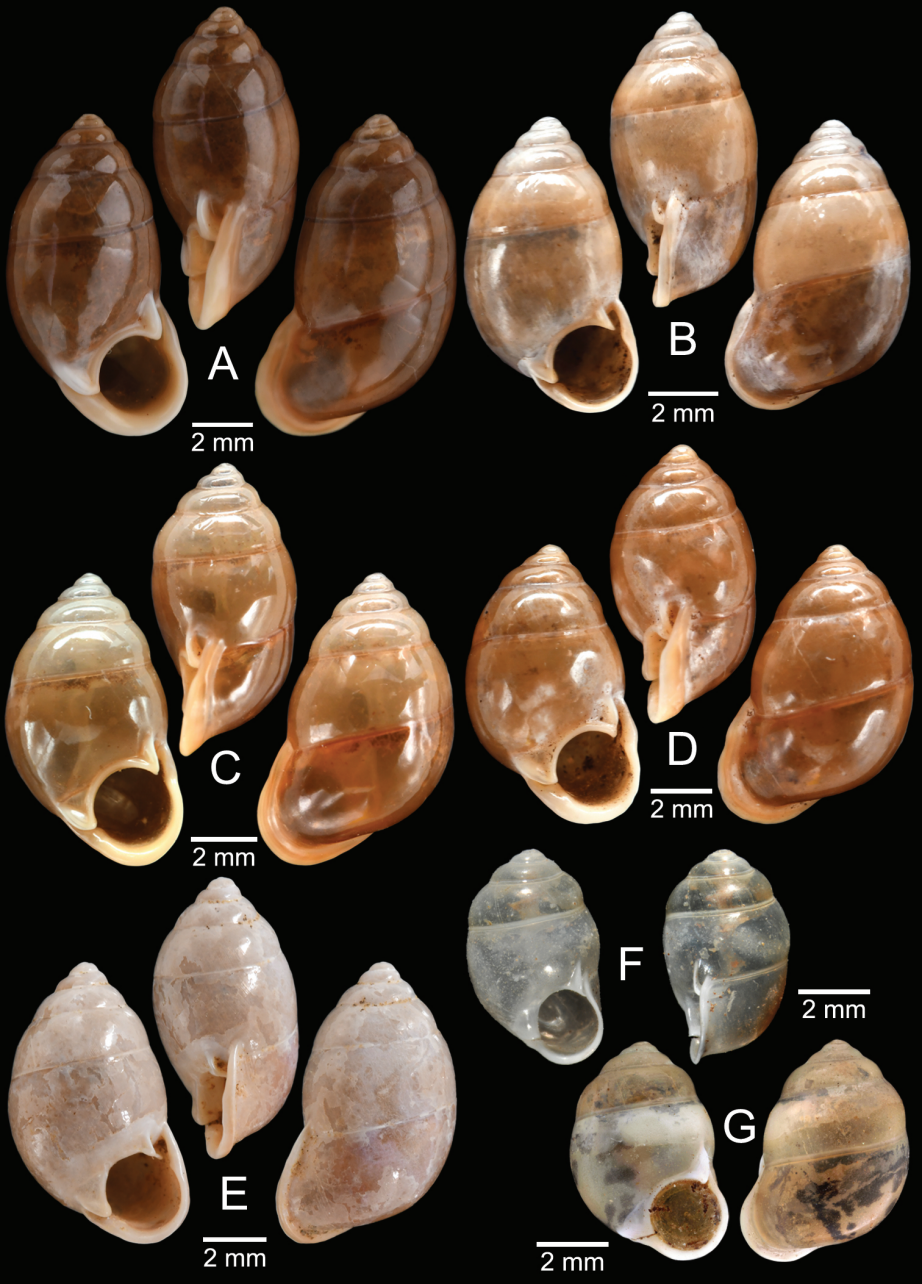
**A–E***Pupinaaureola*: specimens **A**CUMZ 12121 from Tham Thong Panara Temple, Nakhon Sri Thammarat **B**CUMZ 12124 from Talot Cave, Nakhon Sri Thammarat **C**CUMZ 12126 from Khao Huai Haeng Temple, Trang **D**CUMZ 12130 from Sra Morakot, Krabi, and **E**CUMZ 12133 from Khantiphol Cave, Satun **F, G***Pupinapaviei*: paralectotypes **F**MNHN-IM-2000-35837 from Chaîne de l’Eléphant, Kampot, Cambodge and **G**RBINS 525404 from Kampot et forêts de la chaîne de l’Eléphant, Cambodge et Kamchay. Photo: P. Maestrati, MNHN (**F**), F. Trus, RBINS (**G**).

Figs 10D , 19C , 32G, H , 34F , 36A–F , 38A–E

Pupina aureola Stoliczka, 1872: 267, pl. 10, figs 11, 12. Type locality: Penang [Penang State, Malaysia]. Nevill 1878: 299. de Morgan 1885: 414, Poulo Pinang, mont Tchora, près d’Ipoh (Kinta), [Perak State, Malaysia]. von Möllendorff 1891: 345. Sykes 1903: 197, Jalor [Yala Province, Thailand]. van Benthem Jutting 1949: 57, Gunong Pulai, Johore [Johor State, Malaysia]. van Benthem Jutting 1960: 13, limestone hill near kampong Tebing Tinggi, N. of Kangar, Perlis [Malaysia]. Chan 1998a: 4, Ipoh, Perak. Maassen 2001: 40, 41. BEDO 2017: 88. Sutcharit et al. 2018: fig. 5–13f.
Pupina (Tylotoechus) aureola —Kobelt 1902: 307. Laidlaw 1928: 34. Hemmen and Hemmen 2001: 39.
Pupina arula perakensis [non Möllendorff]—Foon et al. 2017: 40, fig. 15c, Ipoh, Perak.
Pupina sp.—Sutcharit et al. 2018: fig. 5–11b.

###### Type material examined.

***Possible syntype***NHMUK 1988.12.4.101 (Figs 34F, 36A) from Pinang.

###### Other material examined.

CUMZ 12112 (2 shells and 6 specimens in ethanol) from Phra Kayang Cave, Kra Buri District, Ranong Province, 4 Apr. 1998. CUMZ 12113 (3 specimens in ethanol) from Na Mueang Waterfall, Ko Samui District, Surat Thani Province, 4 Mar. 2007. CUMZ 12114 (5 specimens in ethanol) from Na Mueang Waterfall, Ko Samui District, Surat Thani Province, 3 Dec. 2015. CUMZ 12115 (4 specimens in ethanol) from Pra Puttabhat Sri Suratth Temple, Kanchanadit District, Surat Thani Province, 6 Dec. 2016. CUMZ 12116 (7 specimens in ethanol; Fig. 19C) from Khiri Rat Phatthana Temple, Wiang Sa District, Surat Thani Province, 4 July 2017. CUMZ 12117 (4 shells and 42 specimens in ethanol; Fig. 32G) from Lod Cave, Nopphitam District, Nakhon Si Thammarat District, 11 Mar. 2017. CUMZ 12118 (1 shell) from Kaeo Surakan Cave, Lan Saka District, Nakhon Si Thammarat Province, 11 Mar. 2017. CUMZ 12119 (6 specimens in ethanol) from Tham Thong Panara Temple, Tham Phannara District, Nakhon Sri Thammarat Province, 4 Apr. 2003. CUMZ 12120 (36 shells and 1 specimen in ethanol) from Tham Thong Panara Temple, Tham Phannara District, Nakhon Sri Thammarat Province, 11 Oct. 2006. CUMZ 12121 (> 100 specimens in ethanol; Figs 32H, 36F, 38A) from Tham Thong Panara Temple, Tham Phannara District, Nakhon Sri Thammarat Province, 11 June 2012. CUMZ 12122 (15 specimens in ethanol) from Tham Thong Panara Temple, Tham Phannara District, Nakhon Sri Thammarat Province, 15 Jan. 2014. CUMZ 12123 (12 shells) from Tham Thong Panara Temple, Tham Phannara District, Nakhon Sri Thammarat Province, 4 July 2017. CUMZ 12124 (3 shells and 1 specimen in ethanol; Figs 36B, 38B) from Talot Cave, Thung Song District, Nakhon Sri Thammarat Province, Thailand, 5 July 2017. CUMZ 12125 (1 shell and 1 specimen in ethanol) from Nam Phut Cave, Mueang Phang Nga District, Phang Nga Province, 6 Aug. 2015. CUMZ 12126 (9 shells; Figs 36C, 38C) from Khao Huai Haeng Temple, Huai Yot District, Trang Province, 6 Oct. 2006. CUMZ 12127 (5 specimens in ethanol) from Ban Khao Poon, Huai Yot District, Trang Province, 6 Oct. 2006. CUMZ 12128 (1 shell) from Trang Botanical Garden, Yan Ta Khao District, Trang Province, 6 Aug. 1999. CUMZ 12129 (4 specimens in ethanol) from Khao Pu Chao Bureau of Monks, Na Yong District, Trang Province, 8 July 2017. CUMZ 12130 (8 shells; Figs 10D, 36D, 38D) from Sra Morakot, Khlong Thom District, Krabi Province, 15 Jan. 2009. CUMZ 12131 (2 specimens in ethanol) from Sra Morakot, Khlong Thom District, Krabi Province, 17 May 2012. CUMZ 12132 (15 specimens in ethanol) from Toe Bu Cliff Viewpoint, Mueang Satun District, Satun Province, 7 Apr. 2008. CUMZ 12133 (7 shells; Figs 36E, 38E) from Khantiphol Cave, Thung Wa District, Satun Province, 13 Jan. 2009.

###### Diagnosis.

Shell ovate to fusiform; last whorl ca. 70–75% of shell height. Apertural lip thickened to highly thickened but not expanded. Parietal tooth thickened, fin-shaped or tooth-like, always located next to but not covering posterior canal; columellar tooth fin-shaped, thickened, located next to anterior canal. Posterior canal slightly bulging outward.

###### Differential diagnosis.

*P.aureola* is most similar to *P.stoliczkai* sp. nov. in shell shape and having both fin-shaped and highly thickened parietal and columellar teeth located next to their respective canals; the posterior canal slightly bulges outward. However, *P.aureola* does not have a furrow between inner and outer peristomes.

###### Distribution.

Malaysia and southern Thailand (Maassen 2001).

###### Remarks.

This species has high variation in shell shape from ovate to fusiform, and the parietal tooth varies from fin-shaped to tooth-like. Despite those shell variations, we assign these shell morphs to *P.aureola* due to the uniform position of a parietal tooth that is always located next to the posterior canal, and a columellar tooth that is always fin-shaped and not extending over the apertural lip.

By comparing with the possible type specimen, the specimen of *P.arulaperakensis* figured in Foon et al. (2017: fig. 15c) from Gunung Datok Plot, Ipoh, Perak should belong to *P.aureola* (Foon, pers. comm.).

##### ﻿ Pupina paviei

Taxon classificationAnimaliaArchitaenioglossaPupinidae

Morlet, 1883

63AD0F53-C026-5EA7-9AD9-60686EF65918

**Figure 39.**
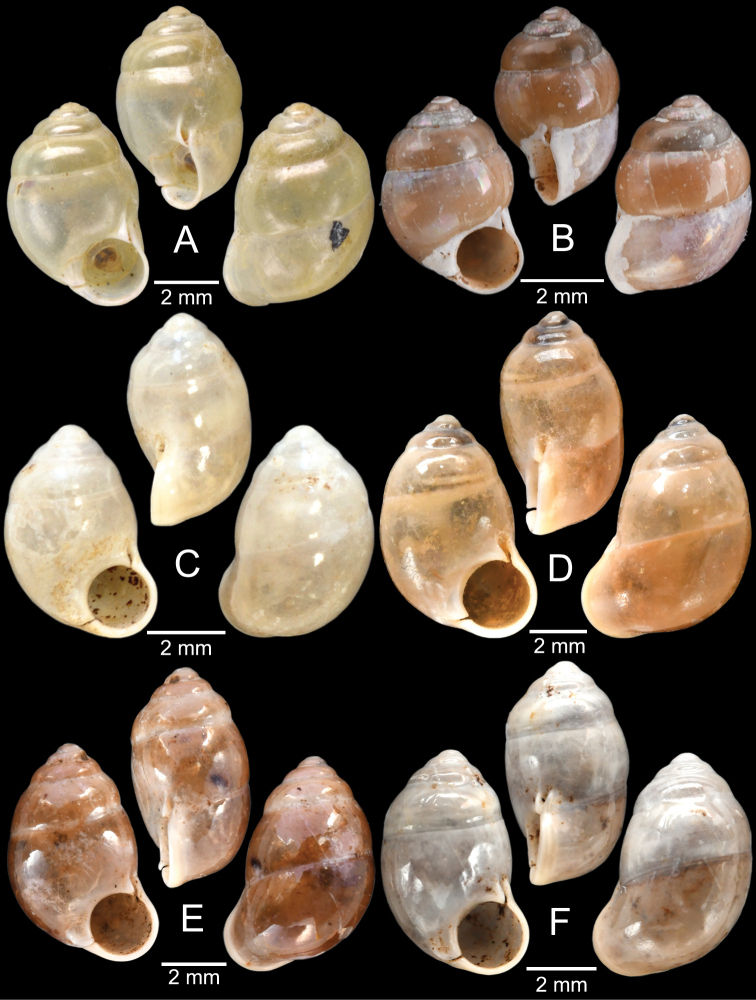
**A, B***Pupinapaviei*: specimens **A**NHMUK ex. Dautzenberg coll. from Kampot, Cambodge and **B**CUMZ 12134 from Lalu, Sa Kaeo **C, D***Pupinatchehelensis*: specimens **C**SMF 109947/6 from Bukit Pondong, Perak and **D**CUMZ 12136 from limestone mountain, Phang Nga **E, F***Pupinadorriisanensis* ssp. nov. **E** holotype CUMZ 12140/1 and **F** specimen CUMZ 12137 from Khao Wang Pha, Nong Bua Lam Phu.

Figs 37F–I , 38F, G , 39A, B

Pupina paviei Morlet, 1883: 107, 108, pl. 4, fig. 4. Type locality: La chaîne de l’Éléphant et les forêts non inondées qui la bordent, particulièrement, près des rapides de Kamchay et aux environs de Kampot [The Elephant Range and the non-flooded forests that border it, particularly near the Kamchay rapids and around Kampot; currently Preah Monivong Bokor National Park, Kampot Province, Cambodia]. Morlet 1889: 152. Fischer 1891: 107. Fischer and Dautzenberg 1904: 431. Morlet 1904: 370, 371, pl. 20, fig. 13, 13a. Fischer-Piette 1950: 153. Fischer 1973: 48. BEDO 2017: 92.
Pupina (Tylotoechus) paviei —Kobelt 1902: 319.

###### Type material examined.

Paralectotypes MNHN-IM-2000-35837 (4 shells; Figs 37F, 38F) from Chaîne de l’Eléphant, Kampot, Cambodge. Paralectotypes RBINS 525404 (76 shells; Figs 37G, 38G) from Kampot et forêts de la chaîne de l’Eléphant, Cambodge et Kamchay.

###### Material examined.

NHMUK ex. Dautzenberg coll. (1 shell; Figs 37I, 39A) from Kampot, Cambodge. CUMZ 12134 (129 shells; Figs 37H, 39B) from Lalu, Ta Phraya District, Sa Kaeo Province, 24 Nov. 2006.

###### Diagnosis.

Shell globose to ovate; last whorl ca. three quarters of shell height. Apertural lip slightly thickened but not expanded. Parietal tooth triangular, not thickened to slightly thickened, covering posterior canal but not extending beyond apertural lip; columellar tooth fin-shaped, slightly thickened, located next to slit-like anterior canal.

###### Differential diagnosis.

*Pupinapaviei* is similar to *P.tongupensis* in a globose shell shape, but differs in having a triangular parietal tooth that is either not thickened or slightly thickened, and a fin-shaped, slightly thickened columellar tooth that is located next to a slit-like anterior canal.

###### Distribution.

Cambodia (Morlet 1883) and Sa Kaeo Province, eastern Thailand.

###### Remarks.

As the original description did not explicitly state that the description of this species was based on a single specimen (nor could this be inferred), the designation of a holotype by Fischer-Piette (1950) in fact constitutes a lectotype designation (ICZN 1999: Art. 74.6).

##### ﻿ Pupina tchehelensis

Taxon classificationAnimaliaArchitaenioglossaPupinidae

Morgan, 1885

E7087A8F-77A4-506B-91AC-E427407680A8

Figs 18D , 37A–C , 39C, D

Pupina tchehelensis Morgan, 1885: 414, 415, pl. 7, fig. 4. Type locality: mont Tchéhèl [possibly the hill in the vicinity of Ipoh, Perak, Malaysia]. von Möllendorff 1891: 346, Bukit Pondong. Maassen 2001: 41. BEDO 2017: 94.
Pupina artata [non Benson]— von Möllendorff 1887 [1886]: 314. von Möllendorff 1891: 345, 346.
Pupina (Tylotoechus) tchehelensis —Kobelt 1902: 323. Laidlaw 1928: 34.
Pupina tchechelensis [sic]— van Benthem Jutting 1949: 57, Sungei Siput, Perak.

###### Material examined.

SMF 109947/6 (6 shells; Figs 37A, 39C) from Bukit Pondong, Perak. CUMZ 12135 (1 shell; Fig. 37B) from Tham Suea Temple, Mueang Krabi District, Krabi Province, 6 Oct. 2006. CUMZ 12136 (7 shells; Figs 18D, 37C, 39D) from limestone mountain, Phang Nga Province, 1 May 1999.

###### Diagnosis.

Shell ovate; last whorl ca. 70–75% of shell height. Apertural lip slightly thickened but not expanded. Parietal tooth sharp, tooth-like, thickened; columellar tooth fin-shaped, slightly thickened, located next to slit-like anterior canal. Posterior canal gradually widening like a keyhole.

###### Differential diagnosis.

*Pupinatchehelensis* is most similar to *P.lowi* and *P.brachysoma* in having a sharp, tooth-like, thickened parietal tooth, a fin-shaped, thickened, columellar tooth that is located next to a slit-like anterior canal, and a posterior canal that is gradually widening. However, *P.tchehelensis* is different from *P.lowi* by having a more ovate shell shape, and differs from *P.brachysoma* in that the apertural lip is not expanded.

###### Distribution.

Malaysia (Maassen 2001) and southern Thailand.

###### Remarks.

Both similar species *P.tchehelensis* and *P.lowi* were originally described by de Morgan (1885) from the same vicinity within Perak, peninsular Malaysia: de Morgan (1885) stated that *P.lowi* is “much larger than *P.tchehelensis*, and this species is distinguished by the shape of its whorls which are much more flattened.” As the type materials of *P.tchehelensis* have not yet been discovered, and *P.tchehelensis* specimens have a slightly higher shell than *P.lowi*, we do not synonymise *P.tchehelensis* with *P.lowi*. Specimens from Thailand have a larger shell than those from Perak, Malaysia (Fig. 37A–C).

##### ﻿ Pupina dorri isanensis

Taxon classificationAnimaliaArchitaenioglossaPupinidae

Jirapatrasilp ssp. nov.

1D5B716E-C5CB-5397-A165-9BC9C8255F0B

Figs 36K, L , 39E, F

###### Type material.

***Holotype***CUMZ 12140/1 (Figs 36K, 39E), 31 Aug. 2020, coll. C. Sutcharit, P. Jirapatrasilp, A. Pholyotha. Measurement: shell height 6.6 mm, shell width 4.6 mm and 5½ whorls. ***Paratypes***CUMZ 12140/2 (22 shells) and NHMUK 20210337 (3 shells), same data as holotype.

###### Type locality.

Pa Pha Ya Temple, Suwannakhuha District, Nong Bua Lam Phu Province, Thailand, 17°37'38.8"N, 102°10'13.7"E, 250 m amsl.

###### Other material examined.

CUMZ 12137 (1 shell; Figs 36L, 39F) from Khao Wang Pha, Na Wang District, Nong Bua Lam Phu Province, 15 Oct. 2007. CUMZ 12138 (1 shell) from Pa Jor Cave, Na Wang District, Nong Bua Lam Phu Province, 15 Oct. 2007. CUMZ 12139 (9 shells) from Tham Suwannakhuha Temple, Suwannakhuha District, Nong Bua Lam Phu Province, 31 Aug. 2020. CUMZ 12141 (2 shells) from Namtok Thao To Forest Park, Mueang Nong Bua Lam Phu District, Nong Bua Lam Phu Province, 31 Aug. 2020. CUMZ 12142 (3 shells and 1 specimen in ethanol) from Phu Pha Lom, Mueang Loei District, Loei Province, 1 Sept. 2020. CUMZ 12143 (1 specimen in ethanol) from Hin Pha Ngam Park, Nong Hin District, Loei Province, 2 Sept. 2020. CUMZ 12144 (1 shell) from Phraya Nakharaj Cave, Phu Pha Man District, Khon Kaen Province, 21 July 2020. CUMZ 12145 (1 specimen in ethanol) from Phu Thong Thep Nimit Temple, Nong Saeng District, Udon Thani Province, 30 Aug. 2020. CUMZ 12170 (1 shell) from Khao Wong Cave, Kaeng Hang Maeo District, Chanthaburi Province, 15 Sept. 2009.

###### Diagnosis.

Shell ovate-fusiform; last whorl ca. 70% of shell height. Apertural lip thickened but not expanded. Parietal tooth triangular, thickened, covering posterior canal, approaching but not extending beyond the outer margin of apertural lip; columellar tooth fin-shaped, thickened, located next to slit-like anterior canal.

###### Differential diagnosis.

This new subspecies is slightly different from the nominotypical subspecies in having the apertural lip, and parietal and columellar teeth more thickened.

###### Description.

Shell height 6.0–6.6 mm; shell width 4.2–4.6 mm. Shell ovate-fusiform, solid, semi-transparent, grey to pale brown, devoid of prominent sculpture on glazed smooth surface. Apex obtuse. Growth lines on shell surface inconspicuous. Whorls 5½, last whorl large (ca. 70% of shell height). Spire angle ca. 75–80°, slightly extended. Sutures slightly impressed, but shallow. Aperture circular; lip thickened (ca. 0.2 mm wide and 0.3–0.4 mm thick) with paler colour, not expanded. Parietal callus not sharply defined and somewhat thickened with paler colour. Peristome interrupted by two canals; posterior canal slit-like ca. 0.7 mm long; anterior canal slit-like continuing horizontally ca. 0.8–0.9 mm. Parietal tooth triangular, thickened (ca. 0.7 mm long, 0.5 mm at its widest and 0.3 mm thick), covering posterior canal, approaching but not extending beyond the outer margin of apertural lip. Columellar tooth thickened (ca. 1.0 mm long, 0.3 mm at its widest and 0.3 mm thick), fin-shaped. Umbilicus closed. Operculum round, thin, flat, multispiral, whitish to pale yellow, opaque corneous with smooth edge.

###### Etymology.

The specific epithet refers to the Thai name “Isan” for the northeastern region of Thailand, where this new subspecies is mainly distributed.

###### Distribution.

Northeastern and eastern Thailand.

###### Remarks.

Although the collecting localities of this new subspecies are ca. 600 km from the known occurrence of the nominotypical subspecies, DNA data and morphometric analyses are required to demonstrate whether these Thai specimens are distinct from the Vietnamese specimens and should be elevated to specific status.

##### ﻿ Pupina latisulci

Taxon classificationAnimaliaArchitaenioglossaPupinidae

Jirapatrasilp sp. nov.

4FD854A6-0368-5DC9-B522-90C0D391CB90

https://zoobank.org/F59DBFAB-6DD5-4E44-A06F-5C18EB83C03A

**Figure 40.**
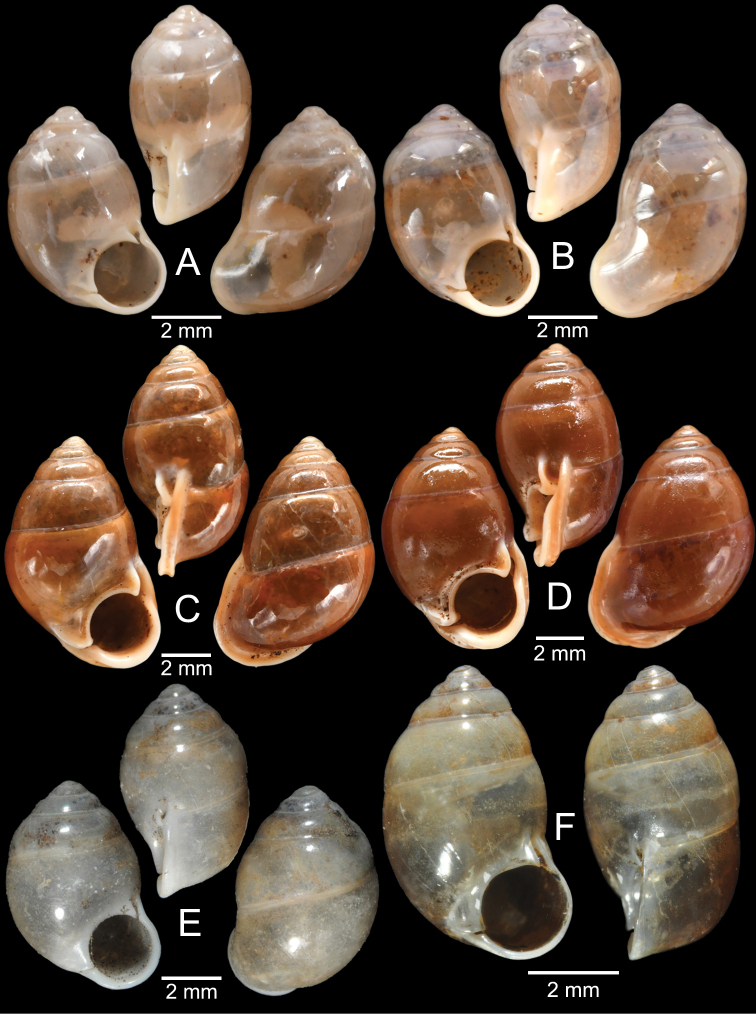
**A, B***Pupinalatisulci* sp. nov. **A** holotype CUMZ 12146/1 and **B** paratype CUMZ 12146/2 from Khao Ok Talu, Phatthalung **C, D***Pupinastoliczkai* sp. nov. **C** holotype CUMZ 12147/1 and **D** paratype NHMUK 20210336 from Wat Ratburana School, Chumpon **E***Pupinalowi*, syntype MNHN-IM-2000-35846 from Lahat, Perak **F***Pupinadorridorri*, paralectotype MNHN-IM-2000-35836 from Haiphong, Vietnam. Photo: P. Maestrati, MNHN (**E, F**).

Figs 37D, E , 40A, B

###### Type material.

***Holotype***CUMZ 12146/1 (Figs 37D, 40A), 9 Apr. 2000, coll. C. Sutcharit, P. Tongkerd, S. Panha. Measurement: shell height 6.0 mm, shell width 4.6 mm and 5¾ whorls. ***Paratypes***CUMZ 12146/2–8 (7 shells; Figs 37E, 40B) and NHMUK 20210338 (2 shells), same data as holotype.

###### Type locality.

Khao Ok Talu, Mueang Phatthalung District, Phatthalung Province, Thailand, 7°37'32.2"N, 100°05'28.5"E, 120 m amsl.

###### Diagnosis.

Shell ovate; last whorl ca. three quarters of shell height. Apertural lip thickened but not expanded. Parietal tooth sharp, tooth-like; columellar tooth sharp, triangular shaped. Both anterior and posterior canals widening like keyholes bordered by its respective tooth and a small bulge of the outer lip.

###### Differential diagnosis.

*Pupinalatisulci* sp. nov. can be distinguished from all other species in the *P.aureola* species group from mainland Southeast Asia by having both anterior and posterior canals widening like keyholes that are bordered by its respective tooth and a small bulge of the outer lip.

###### Description.

Shell height 4.0–4.5 mm; shell width 5.9–6.3 mm. Shell ovate, solid, semi-transparent, whitish to pale brown, devoid of prominent sculpture on glazed smooth surface. Apex obtuse. Growth lines on shell surface inconspicuous. Whorls 5¾, last whorl large (ca. three quarters of shell height). Spire angle ca. 90°, slightly extended. Sutures slightly impressed, but shallow. Aperture circular; lip thickened (ca. 0.1–0.2 mm wide and 0.1–0.2 mm thick) with paler colour, not expanded. Parietal callus not sharply defined and somewhat thickened with paler colour. Peristome interrupted by two canals; posterior canal ca. 0.6 mm long, 0.4 mm at its widest, continuing obliquely to form a narrow groove that widens upward like a keyhole; bordered by parietal tooth and more thickened lip appearing as a small bulge. Anterior canal slit-like continuing horizontally ca. 0.7–0.8 mm, widening towards outer margin like a keyhole, bordered by columellar tooth and more thickened lip. Parietal tooth sharp, thickened (ca. 0.6 mm long, 0.4 mm at its widest and 0.2 mm thick), tooth-like. Columellar tooth thickened (ca. 0.6 mm long, 0.9 mm at its widest and 0.2 mm thick), sharp, triangular shaped. Umbilicus closed. Operculum unknown.

###### Etymology.

The specific epithet *latisulci* is derived from the Latin word *latus* meaning wide and *sulci* [plural form of *sulcus*] meaning furrow or groove, which describes the widening of both anterior and posterior canals in the new species.

###### Distribution.

This new species is found from Phatthalung Province, southern Thailand.

##### ﻿ Pupina stoliczkai

Taxon classificationAnimaliaArchitaenioglossaPupinidae

Jirapatrasilp sp. nov.

9745E944-1F0E-5C99-B50D-8EC76072331F

https://zoobank.org/80A5D354-B516-4F67-9F08-817A29FBBEFB

Figs 36G, H , 40C, D

###### Type material.

***Holotype***CUMZ 12147/1 (Figs 36G, 40C), 10 Sept. 2016, coll. R. Srisonchai, A. Pholyotha, T. Seesamut. Measurement: shell height 9.4 mm, shell width 6.3 mm and 6½ whorls. ***Paratypes***CUMZ 12147/2 (1 specimen in ethanol) and NHMUK 20210336 (1 shell; Figs 36H, 40D), same data as holotype.

###### Type locality.

Wat Ratburana School, Lang Suan District, Chumpon Province, Thailand, 9°56'18.0"N, 99°02'25.5"E, 20 m amsl.

###### Diagnosis.

Shell ovate-fusiform; last whorl ca. 70% of shell height. Apertural lip highly thickened and slightly expanded; with a furrow between inner and outer peristomes; inner peristome thickened, cord-like. Both parietal and columellar teeth fin-shaped, very thickened, always located next to and not covering its respective canal. Posterior canal slightly bulging outward.

###### Differential diagnosis.

*P.stoliczkai* sp. nov. is most similar to *P.aureola* in shell shape in having both fin-shaped and highly thickened parietal and columellar teeth located next to their respective canals, and the posterior canal slightly bulging outward. However, *P.stoliczkai* sp. nov. has a furrow between inner and outer peristomes, with inner peristome thickened and cord-like.

###### Description.

Shell height 6.3–6.4 mm; shell width 9.0–9.5 mm. Shell ovate-fusiform, solid, semi-transparent, reddish brown, devoid of prominent sculpture on glazed smooth surface. Apex obtuse. Growth lines on shell surface inconspicuous. Whorls 6–6½, last whorl large (ca. 70% of shell height). Spire angle ca. 80–90°; slightly extended. Sutures slightly impressed, but shallow. Aperture circular; lip highly thickened (ca. 0.4–0.5 mm wide and 0.6–0.7 mm thick) with paler colour, slightly expanded. Aperture with a furrow between inner and outer peristomes, with inner peristome thickened, cord-like. Parietal callus sharply defined and thickened with paler colour. Peristome interrupted by two canals; posterior canal ca. 1.4 mm long and 0.7 mm at its widest, slightly bulging outward, continuing obliquely and widening vertically upward when observed from lateral view. Anterior canal curved and continuing obliquely upward ca. 2.0 mm. Parietal tooth fin-shaped, highly thickened (ca. 1.5 mm long, 0.5 mm at its widest and 0.3 mm thick), always located next to and not covering posterior canal. Columellar tooth fin-shaped, highly thickened (ca. 1.9 mm long, 0.5 mm at its widest and 0.3 mm thick), located next to anterior canal. Umbilicus closed. Operculum round, thick, flat, multispiral, whitish to pale yellow, opaque corneous with smooth edge.

###### Etymology.

The specific epithet is dedicated to F. Stoliczka, a Czech palaeontologist and zoologist, who described *P.aureola*, to which this new species is associated with.

###### Distribution.

This new species is found only from the type locality.

#### Species of group III (*P.aureola* species group) from other parts of mainland Southeast Asia not recorded for Thailand

##### ﻿ Pupina lowi

Taxon classificationAnimaliaArchitaenioglossaPupinidae

Morgan, 1885

FCE50968-C667-5EA3-8B4B-D515E13746FF

Figs 37K , 40E

Pupina lowi Morgan, 1885: 414, pl. 7, fig. 3a–d. Type locality: Lahat, Kinta [Perak State, Malaysia]. von Möllendorff 1891: 345. Sykes 1903: 197, Gunong Inas, Perak. van Benthem Jutting 1949: 57, Larut Mills, Perak. van Benthem Jutting 1960: 13, limestone hill Kaki Bukit, near kampong Wang Tangga, Perlis [Malaysia]. Maassen 2001: 41.
Pupina (Tylotoechus) lowi —Kobelt 1902: 317. Laidlaw 1928: 34.
Pupina artata [non Benson]—Berry 1963: pl. 6, fig. 36. Foon et al. 2017: 40, fig. 15b, Ipoh, Perak.

###### Type material examined.

***Syntype***MNHN-IM-2000-35846 (1 shell; Figs 37K, 40E) from Lahat, Perak.

###### Diagnosis.

Shell globose; last whorl ca. three quarters of shell height. Apertural lip slightly thickened but not expanded. Parietal tooth sharp, tooth-like, thickened; columellar tooth fin-shaped, thickened, located next to slit-like anterior canal. Posterior canal gradually widening like keyhole.

###### Differential diagnosis.

*Pupinalowi* is most similar to *P.tchehelensis* and *P.brachysoma* in having a sharp, tooth-like, thickened parietal tooth, a fin-shaped, thickened columellar tooth that is located next to a slit-like anterior canal, and a posterior canal that is gradually widening. However, *P.lowi* is different from *P.tchehelensis* by having a more globose shell shape, and different from *P.brachysoma* in that an apertural lip is not expanded.

###### Distribution.

Perak and Perlis States, Malaysia (Maassen 2001).

###### Remarks.

By comparing with the type specimen, the specimens of *P.artata* figured in Berry (1963: pl. 6, fig. 36) and Foon et al. (2017: fig. 15b) from Bat Cave Hill Plot 2, Ipoh, Perak should belong to *P.lowi* (Foon, pers. comm.). The specimen of *P.lowi* figured in BEDO (2017: 91) should constitute a different species as it is different from the syntype figured here in having a smaller, sharper parietal tooth revealing a wide posterior canal and an earlobe-shaped columellar tooth covering the anterior canal. Thus, that specimen should belong to the *P.arula* species group instead (see above).

##### ﻿ Pupina dorri dorri

Taxon classificationAnimaliaArchitaenioglossaPupinidae

Dautzenberg, 1894

2D6DC63D-8E98-5592-AF5D-16BDA480EAF0

**Figure 41.**
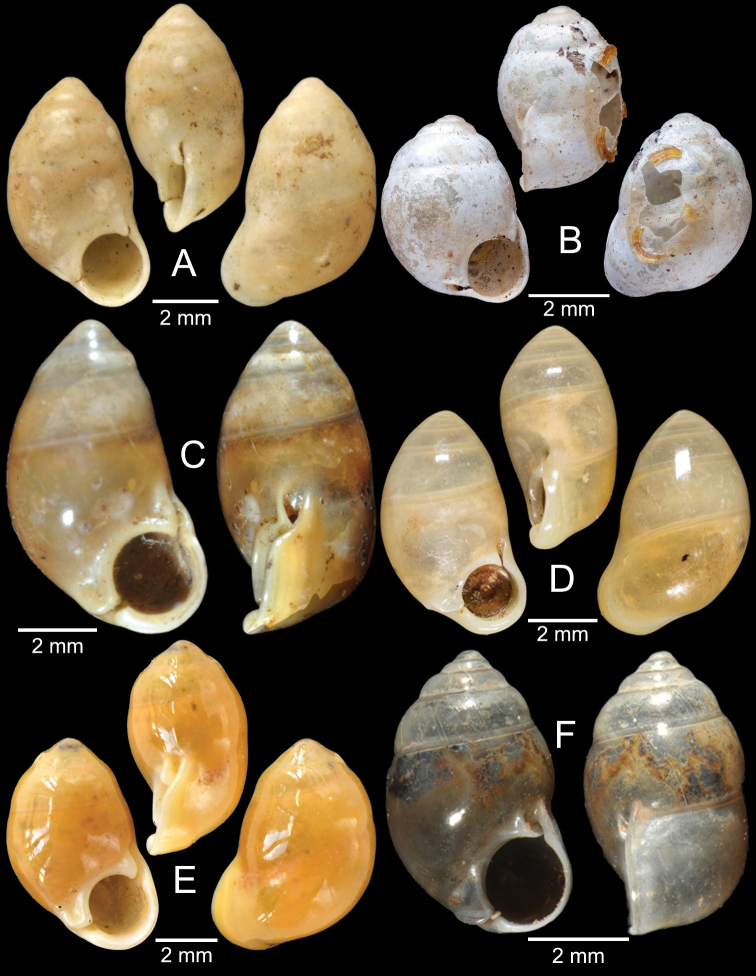
**A***Pupinadorridorri*, specimen NHMUK ex. A.J. Piele Colln. Acc. No. 2242 from Haiphong, Vietnam **B***Pupinatongupensis*, syntype NHMUK 1906.4.4.38 from Tongoop Pass, Arakan Hills, east side **C***Pupinaanceyi*, syntype MNHN-IM-2000-35833 from Lang-Son and That-Khé **D***Pupinalaffonti*, syntype MNHN-IM-2000-9656 from Poulo-Condor **E***Pupinasolidula*, lectotype SMF 109915/1 from Lang Son, Tonkin **F***Pupinabrachysoma*, syntype MNHN-IM-2000-9652 from Haut Tonkin. Photo: J. Ablett, H. Taylor, NHM (**B**), P. Maestrati, M. Caballer, MNHN (**C–F**).

Figs 36I, J , 40F , 41A

Pupina flava [non Möllendorff]—Morlet 1887: 261. Fischer 1891: 107.
Pupina dorri Dautzenberg, 1894 [1893]: 164, 165, pl. 8, fig. 3, 3a–c. Type locality: montagnes des environs d’Haïphong [Haiphong, Vietnam]. Fischer 1898: 333. Fischer and Dautzenberg 1904: 431, iles du golfe du Tonkin. Dautzenberg and Fischer 1905: 171. Fischer-Piette 1950: 160. Fischer 1963: 33. Do et al. 2015: 126, fig. 5f, Son La Province, Vietnam.
Pupina (Tylotoechus) dorri —Kobelt 1902: 311.

###### Type material examined.

***Lectotype***MNHN-IM-2000-35835 from Haiphong. Paralectotypes MNHN-IM-2000-35836 (7 shells; Figs 36I, 40F) from Haiphong, Vietnam.

###### Other material examined.

NHMUK ex. A.J. Piele Colln. Acc. No. 2242 (3 shells; Figs 36J, 41A) from Haiphong, Vietnam.

###### Diagnosis.

Shell ovate-fusiform; last whorl ca. 70–75% of shell height. Apertural lip slightly thickened but not expanded. Parietal tooth triangular, slightly thickened, covering posterior canal, approaching but not extending beyond the outer margin of apertural lip; columellar tooth fin-shaped, slightly thickened, located next to slit-like anterior canal.

###### Differential diagnosis.

*Pupinadorri* can be distinguished from all other species in the *P.aureola* species group from mainland Southeast Asia by having a triangular, slightly thickened parietal tooth that is covering a posterior canal, and the parietal tooth approaching but not extending beyond the outer margin of apertural lip.

###### Distribution.

Northern Vietnam (Do et al. 2015).

###### Remarks.

As the original description did not explicitly state that the description of this species was based on a single specimen (nor could this be inferred), the designation of a holotype by Fischer-Piette (1950) in fact constitutes a lectotype designation (ICZN 1999: Art. 74.6).

##### ﻿ Pupina tongupensis

Taxon classificationAnimaliaArchitaenioglossaPupinidae

Godwin-Austen, 1897

F1B80F03-87F0-5001-828F-66E93760AFB1

Figs 37J , 41B

Pupina tongupensis Godwin-Austen, 1897: 41, pl. 69, fig. 5, 5a. Type locality: Tongoop Pass, Arakan Hills, east side [probably refers to Toungup Road and the area on Arakan Hills, the path which connects Toungup, Rakhine State to Padaung, Pyay District, Bago Region, Myanmar].
Pupina (Tylotoechus) tongupensis —Kobelt 1902: 323. Gude 1921: 197, 198.

###### Type material examined.

***Syntypes***NHMUK 1906.4.4.38 (2 shells; Figs 37J, 41B) from Tongoop Pass, Arakan Hills, east side.

###### Diagnosis.

Shell globose; last whorl ca. three quarters of shell height. Apertural lip very slightly thickened, not expanded. Both parietal and columellar teeth thin, sharp, tooth-like; columellar tooth next to slit-like but widening anterior canal.

###### Differential diagnosis.

*Pupinatongupensis* is similar to *P.paviei* in a globose shell shape, but differs in having thin, sharp, tooth-like parietal and columellar teeth, and a slit-like but widening anterior canal

###### Distribution.

Known only from the type locality (Gude 1921).

##### ﻿ Pupina anceyi

Taxon classificationAnimaliaArchitaenioglossaPupinidae

Bavay & Dautzenberg, 1899

13611EDA-E3EF-5FED-9CB8-177C60F0F328

Figs 37L , 41C

Pupina anceyi Bavay & Dautzenberg, 1899: 53, 54, pl. 3, fig. 5, 5a. Type locality: Entre Lang-Son [Lang Son Province, Vietnam] et That-Khé [That Khe, Lang Son Province, Vietnam]. Fischer and Dautzenberg 1904: 431. Fischer-Piette 1950: 167. Do et al. 2015: 126, fig. 5e, Son La Province, Vietnam.
Pupina (Tylotoechus) anceyi —Kobelt 1902: 306.
Eupupina anceyi —Dautzenberg and Fischer 1908: 207, Mo-Xat [west of Quang Uyen, Cao Bang Province, Vietnam].

###### Type material examined.

***Lectotype***MNHN-IM-2000-35833 (Figs 37L, 41C) from Lang-Son and That-Khé.

###### Diagnosis.

Shell fusiform; last whorl ca. 65% of shell height. Suture very shallow. Apertural lip highly thickened but not expanded. Parietal tooth triangular, thickened, covering posterior canal, approaching but not extending beyond the outer margin of apertural lip; columellar tooth fin-shaped, thickened, located next to slit-like anterior canal.

###### Differential diagnosis.

*Pupinaanceyi* is similar to *P.laffonti* in having a fusiform shell shape with very shallow suture and a fin-shaped, thickened, columellar tooth that is located next to a slit-like anterior canal, but differs in having a triangular, thickened, parietal tooth covering a posterior canal, and the parietal tooth approaching but not extending beyond the outer margin of apertural lip.

###### Distribution.

Northern Vietnam (Do et al. 2015).

###### Remarks.

As the original description did not explicitly state that the description of this species was based on a single specimen (nor could this be inferred), the designation of a holotype by Fischer-Piette (1950) in fact constitutes a lectotype designation (ICZN 1999: Art. 74.6).

##### ﻿ Pupina laffonti

Taxon classificationAnimaliaArchitaenioglossaPupinidae

Ancey, 1899

C4386ABD-B879-58C8-8016-D4A92906CD33

Figs 37M , 41D

Pupina laffonti Ancey in Bavay & Dautzenberg, 1899: 51–53, pl. 3, fig. 4, 4a. Type locality: Ile de Poulo Condor [Con Dao Island, Vietnam]. Fischer and Dautzenberg 1904: 431. Fischer-Piette 1950: 167. Wood and Gallichan 2008: 57, pl. 25, figs 4, v.

###### Type material examined.

***Lectotype***MNHN-IM-2000-9656 (Figs 37M, 41D) from Poulo-Condor. Paralectotypes NMW.1955.158.24152 figured in Wood and Gallichan (2008: pl. 25, figs 4, v).

###### Diagnosis.

Shell fusiform; last whorl ca. 70% of shell height. Suture very shallow. Apertural lip highly thickened but not expanded. Parietal tooth sharp, tooth-like, thickened; columellar tooth fin-shaped, thickened, located next to slit-like anterior canal. Posterior canal gradually widening like keyhole.

###### Differential diagnosis.

*Pupinalaffonti* is similar to *P.anceyi* in having a fusiform shell shape with very shallow suture, and a fin-shaped, thickened columellar tooth, located next to slit-like anterior canal, but differs in having a sharp, tooth-like, thickened parietal tooth, and a posterior canal that is gradually widening like keyhole.

###### Distribution.

Known only from the type locality (Fischer and Dautzenberg 1904).

###### Remarks.

As the original description did not explicitly state that the description of this species was based on a single specimen (nor could this be inferred), the designation of a holotype by Fischer-Piette (1950) in fact constitutes a lectotype designation (ICZN 1999: Art. 74.6).

##### ﻿ Pupina solidula

Taxon classificationAnimaliaArchitaenioglossaPupinidae

Möllendorff, 1901

4B467198-692D-57E7-893E-02F519E43DA6

Figs 37N , 41E

Pupina (Tylotechus) solidula Möllendorff, 1901: 81. Type locality: Lang-son [Lang Son Province, Vietnam], Mansongebirge [Mou Son Mountain, northern Vietnam]. Zilch 1957: 45, pl. 2, fig. 14.
Pupina solidula —Fischer and Dautzenberg 1904: 432, Lang-Son; Monts Mauson, Tonkin; ile Ba-Moun, golfe du Tonkin [Bah Mun Island].

###### Type material examined.

***Lectotype***SMF 109915/1 (Figs 37N, 41E) from Lang Son, Tonkin.

###### Diagnosis.

Shell yellow, ovate-fusiform; last whorl ca. three quarters of shell height. Suture very shallow. Apertural lip highly thickened but not expanded. Parietal tooth fin-shaped, thickened, not covering posterior canal; columellar tooth fin-shaped, thickened, located next to slit-like anterior canal.

###### Differential diagnosis.

*Pupinasolidula* can be distinguished from all other species in the *P.aureola* species group from mainland Southeast Asia by having a glossy, yellow shell with very shallow suture.

###### Distribution.

Northeast Vietnam (Fischer and Dautzenberg 1904).

##### ﻿ Pupina brachysoma

Taxon classificationAnimaliaArchitaenioglossaPupinidae

Ancey, 1904

B1B4E121-EFDF-5693-B99A-96A381BC23B1

Figs 36M , 41F

Pupina brachysoma Ancey in Bavay & Dautzenberg, 1904 [1903]: 230, 231, pl. 10, figs 15, 16. Type locality: Haut-Tonkin [northern Vietnam]. Fischer-Piette 1950: 171. Wood and Gallichan 2008: 31, pl. 25, figs 5, vi.

###### Type material examined.

***Lectotype***MNHN-IM-2000-9652 (Figs 36M, 41F) from Haut Tonkin. Paralectotypes NMW.1955.158.24153 figured in Wood and Gallichan (2008: pl. 25, figs 5, vi).

###### Diagnosis.

Shell ovate-fusiform; last whorl ca. three quarters of shell height. Apertural lip somewhat thickened, slightly expanded. Parietal tooth sharp, tooth-like, thickened; columellar tooth fin-shaped, slightly thickened, located next to slit-like anterior canal. Posterior canal widened.

###### Differential diagnosis.

*Pupinabrachysoma* is most similar to *P.tchehelensis* and *P.lowi* in having a sharp, tooth-like, thickened parietal tooth, a fin-shaped, thickened columellar tooth that is located next to a slit-like anterior canal, and a posterior canal that is gradually widening. However, *P.brachysoma* is different from both *P.tchehelensis* and *P.lowi* by a more ovate-fusiform shell shape, and a less thickened but slightly expanded apertural lip. *Pupinabrachysoma* is also similar to *P.dorridorri* in shell shape, but differs in having a gradually widening posterior canal.

###### Distribution.

Known only from the type locality (Bavay and Dautzenberg 1904).

###### Remarks.

As the original description did not explicitly state that the description of this species was based on a single specimen (nor could this be inferred), the designation of a holotype by Fischer-Piette (1950) in fact constitutes a lectotype designation (ICZN 1999: Art. 74.6).

##### ﻿ Pupina douvillei

Taxon classificationAnimaliaArchitaenioglossaPupinidae

Dautzenberg & Fischer, 1906

8D0B9C0B-E143-512C-B594-5B2C1970A4F8

Fig. 37O

Pupina douvillei Dautzenberg & Fischer, 1906 [1905]: 440, pl. 10, figs 10–12. Type locality: Ha-Giang, Tonkin [Vietnam]. Fischer 1963: 33.

###### Type material examined.

***Holotype***MNHN-IM-2000-35532 (Fig. 37O) from Ha-Giang, Tonkin.

###### Diagnosis.

Shell ovate-fusiform; last whorl ca. three quarters of shell height. Apertural lip thickened but not expanded. Parietal tooth fin-shaped, thickened, located next to wide posterior canal; columellar tooth fin-shaped, thickened, located next to slit-like anterior canal.

###### Differential diagnosis.

*Pupinadouvillei* can be distinguished from all other species in the *P.aureola* species group from mainland Southeast Asia by having a high spired shell and a fin-shaped, thickened parietal tooth that is located next to a wide posterior canal.

###### Distribution.

Known only from the type locality (Fischer 1963).

###### Remarks.

As *P.douvillei* was described based on a single specimen as explicitly stated in the original description, that specimen is the holotype fixed by monotypy (ICZN 1999: Art. 73.1.2).

#### Species from other parts of mainland Southeast Asia with uncertain affiliation

##### ﻿ Pupina porcellana

Taxon classificationAnimaliaArchitaenioglossaPupinidae

Rochebrune, 1881

3E52B91F-5D6E-5D7B-AD46-16E091D98933

Pupina porcellana Rochebrune, 1881: 62. Type locality: Montagnes de Chaudoe, Cambodge [Chau Doc, An Giang Province, Vietnam]. Fischer 1891: 108. Fischer and Dautzenberg 1904: 431. BEDO 2017: 93.

###### Remarks.

This species has an uncertain affiliation as there is no figure in the original description or in other later works. The type series were searched for in March 2022 and could not be located in the MNHN by B. Páll-Gergely or P. Bouchet, and were deemed presumably lost (B. Páll-Gergely and P. Bouchet, pers. comm.).

## ﻿Discussion

This is the first comprehensive study focusing on the family Pupinidae in Thailand since the checklists of Thai land snails by Hemmen and Hemmen (2001) and BEDO (2017). This study reports a total of 30 Thai nominal species with two subspecies from seven pupinid genera, an increase from 12 species from four genera in Hemmen and Hemmen (2001), and from 25 species with one subspecies from five genera in BEDO (2017). The updated information in this study includes the recent discovery of *Pseudopomatiascaligosus* from northern Thailand (Páll-Gergely and Hunyadi 2018b) with the discovery of two new *Pseudopomatias* species and three new records (*Coptocheilussumatranus*, *Pupinellamansuyi*, and *Rhaphaulustonkinensis*). Five species and one subspecies of *Pupina* are newly described herein after the discovery of new *Pupina* species from Thailand more than a century ago. BEDO (2017) reported three *Pupina* species, *P.excisa*, *P.lowi*, and *P.porcellana*, which were not discovered in our survey. Comparing our faunal list to the record of land snails from West Malaysia, Maassen (2001) reported a total of 15 species from five pupinid genera, wherein *Pseudopomatias* and *Pupinella* were not reported. Other related pupinid genera, i.e., *Streptaulus* (which is related to *Rhaphaulus*) and *Vargapupa* (related to *Pseudopomatias*), were not discovered from Thailand in this study, suggesting that these genera are rare and restricted to limited geographic ranges (Páll-Gergely et al. 2014, 2015, 2017; Páll-Gergely and Grego 2019). More thorough investigations, especially along the country’s border, combined with other sampling methods (e.g., litter sieving) may uncover more species or even genera in the family Pupinidae.

## Supplementary Material

XML Treatment for Pupinidae

XML Treatment for Pupinellinae

XML Treatment for Coptocheilus

XML Treatment for Coptocheilus sectilabris

XML Treatment for Coptocheilus sumatranus

XML Treatment for Pollicaria

XML Treatment for Pollicaria mouhoti mouhoti

XML Treatment for Pollicaria mouhoti monochroma

XML Treatment for Pollicaria myersii

XML Treatment for Pseudopomatias

XML Treatment for Pseudopomatias caligosus

XML Treatment for Pseudopomatias doiangkhangensis

XML Treatment for Pseudopomatias pallgergelyi

XML Treatment for Pupinella

XML Treatment for Pupinella mansuyi

XML Treatment for Pupinella illustris

XML Treatment for Pupinella sonlaensis

XML Treatment for Pupinella thaitranbaii

XML Treatment for Rhaphaulus

XML Treatment for Rhaphaulus lorraini

XML Treatment for Rhaphaulus perakensis

XML Treatment for Rhaphaulus ascendens

XML Treatment for Rhaphaulus tonkinensis

XML Treatment for Rhaphaulus chrysalis

XML Treatment for Tortulosa

XML Treatment for Tortulosa tortuosa

XML Treatment for Pupininae

XML Treatment for Pupina

XML Treatment for Pupina artata

XML Treatment for Pupina pallens

XML Treatment for Pupina limitanea

XML Treatment for Pupina bensoni

XML Treatment for Pupina hungerfordiana

XML Treatment for Pupina billeti

XML Treatment for Pupina verneaui

XML Treatment for Pupina peguensis

XML Treatment for Pupina crosseana

XML Treatment for Pupina siamensis

XML Treatment for Pupina bilabiata

XML Treatment for Pupina godwinausteni

XML Treatment for Pupina arula

XML Treatment for Pupina mouhoti

XML Treatment for Pupina vescoi

XML Treatment for Pupina exclamationis

XML Treatment for Pupina perakensis

XML Treatment for Pupina excisa

XML Treatment for Pupina aureola

XML Treatment for Pupina paviei

XML Treatment for Pupina tchehelensis

XML Treatment for Pupina dorri isanensis

XML Treatment for Pupina latisulci

XML Treatment for Pupina stoliczkai

XML Treatment for Pupina lowi

XML Treatment for Pupina dorri dorri

XML Treatment for Pupina tongupensis

XML Treatment for Pupina anceyi

XML Treatment for Pupina laffonti

XML Treatment for Pupina solidula

XML Treatment for Pupina brachysoma

XML Treatment for Pupina douvillei

XML Treatment for Pupina porcellana
